# Mining Chemical Activity Status from High-Throughput Screening Assays

**DOI:** 10.1371/journal.pone.0144426

**Published:** 2015-12-14

**Authors:** Othman Soufan, Wail Ba-alawi, Moataz Afeef, Magbubah Essack, Valentin Rodionov, Panos Kalnis, Vladimir B. Bajic

**Affiliations:** 1 King Abdullah University of Science and Technology (KAUST), Computational Bioscience Research Center (CBRC), Thuwal 23955–6900, Saudi Arabia; 2 King Abdullah University of Science and Technology (KAUST), KAUST Catalysis Center (KCC), Thuwal 23955–6900, Saudi Arabia; 3 King Abdullah University of Science and Technology (KAUST), Infocloud Group, Computer, Electrical and Mathematical Sciences and Engineering Division (CEMSE), Thuwal 23955–6900, Saudi Arabia; University of East Anglia, UNITED KINGDOM

## Abstract

High-throughput screening (HTS) experiments provide a valuable resource that reports biological activity of numerous chemical compounds relative to their molecular targets. Building computational models that accurately predict such activity status (active vs. inactive) in specific assays is a challenging task given the large volume of data and frequently small proportion of active compounds relative to the inactive ones. We developed a method, DRAMOTE, to predict activity status of chemical compounds in HTP activity assays. For a class of HTP assays, our method achieves considerably better results than the current state-of-the-art-solutions. We achieved this by modification of a minority oversampling technique. To demonstrate that DRAMOTE is performing better than the other methods, we performed a comprehensive comparison analysis with several other methods and evaluated them on data from 11 PubChem assays through 1,350 experiments that involved approximately 500,000 interactions between chemicals and their target proteins. As an example of potential use, we applied DRAMOTE to develop robust models for predicting FDA approved drugs that have high probability to interact with the thyroid stimulating hormone receptor (TSHR) in humans. Our findings are further partially and indirectly supported by 3D docking results and literature information. The results based on approximately 500,000 interactions suggest that DRAMOTE has performed the best and that it can be used for developing robust virtual screening models. The datasets and implementation of all solutions are available as a MATLAB toolbox online at www.cbrc.kaust.edu.sa/dramote and can be found on Figshare.

## Introduction

Experimental screening of chemical compounds for their biological activity has partial coverage and leaves millions of chemical compounds untested [1]. Such experiments are usually pursued through high-throughput screening (HTS) assays in which chemical molecules (e.g. drugs) are tested against specific biological targets (e.g. protein) [2]. With existence of emerging and growing public repositories (e.g. PubChem database [3]) that provide access to biological activity information from HTS experiments, there is an opportunity to develop computational methods to predict the biological activities of millions of chemical compounds that remain untested [3, 4]. For example, data mining techniques may help narrow down promising candidate chemicals aimed at interaction with specific molecular targets before they are experimentally evaluated [5–7]. This, in principle, may help in speeding up the drug discovery process. Developing accurate prediction models for *in silico* HTS is however challenging. For datasets such as those obtained from HTS assays, achieving high prediction accuracy may be misleading since this may be accompanied by unacceptable false positive rate [8] as high accuracy does not always imply small proportion of false predictions. The fact that should be considered is that HTS experimental data is usually characterized by a great disproportion of active and inactive chemical compounds out of thousands screened [9]. This class imbalance may affect accuracy and precision of resultant predictors of activity status in individual assays [10]. If the imbalance ratio (IR) between the inactive and active compound classes can be adjusted, the performance may improve [10–12].

In this study we examine robust solutions that can be used for *in silico* screening of compound activity status in individual HTS assays that are characterized by great class imbalance. For such cases, several data mining techniques have been developed to model chemical-target interactions [13–16]. These techniques differ from virtual screening based on ligand-protein docking [17], as they do not require any prior knowledge about the 3D surface representation of the target and its cognate interactor. Also, once trained, data mining models are usually faster than ligand-protein docking models in predicting biological activity status of a given chemical compound [18].Several web tools for predicting chemical-protein interactions have also been developed [19–22].Decision trees are used by Han *et al*. [23] to predict the activity of a chemical compound based on the standard set of PubChem features that define chemical fingerprints [24]. The study demonstrated that the great imbalance between data classes limits classification accuracy. Different studies [25, 26] were focused on finding solution to this problem. Cost-sensitive classifiers were explored by Schierz *et al*. [25] to assign a prior importance weight to the minority class for training, whereas an optimization procedure for selecting informative samples, specifically aimed at enhancing performance of support vector machines (SVMs) was also explored [26].

Although good progress has been achieved for building predictive models for HTS data, there are still many issues in current methods that need to be investigated further.

First, many studies have developed prediction models for HTS data without considering precision or other precision relevant scores like F_1_Score in optimizing the performance of these models. Recently, some studies [27–29] explored applying random under-sampling or synthetic over-sampling techniques to some assays (BioAssays) from the PubChem database. These studies did not focus on or report the precision of predictions and their impact over the number of false positives, which are highly relevant [9, 11, 12, 30]. In the case of *in silico* screening of chemical activity status, the increased precision will reduce the number of falsely predicted candidate compounds thus reducing the cost of the potential follow up laboratory experiments [8].

Second, generating and selecting a good subset of features is an important step in developing a well-performing prediction model, and may help in the cases of data with large class imbalance [31, 32]. Few efforts, however, have been dedicated for finding strong discriminating features for HTS data [26, 33, 34].

To tackle the above-mentioned problems, in this study we examine robust solutions to be used for *in silico* screening of compound activity status in individual HTS assays. For this purpose, we run experiments using various state-of-the-art methods and compare their effect on prediction of chemical activity status using different performance metrics. Also, we developed a variant method, DRAMOTE, based on ideas from active learning, which favors selection of precision-informative training samples. We describe the data by a rich set of features that includes PubChem fingerprint features. The set of feature we generated is, to the best of our knowledge, the most comprehensive feature set used for problems of this type. This set of features was further subjected to a feature selection method to propose a set of features that may result in an improved prediction performance in comparison to the PubChem fingerprint features alone. The results of 1,350 *in silico* experiments that involved close to 500,000 interactions, suggest that DRAMOTE is the most efficient variant of data preprocessing in the case of great class imbalance based on the datasets from PubChem we used. DRAMOTE, which favors selection of interactions that enhances the overall precision of a learning model, improves F_1_Score on average by over 41%, relative to other methods. Finally, we illustrate the usefulness of our DRAMOTE method through a case study of screening all FDA approved drugs in the DrugBank database [35] against the thyroid stimulating hormone receptor (TSHR) in humans and suggest top 10 candidates that potentially interact with TSHR. Our findings are further partially and indirectly supported by 3D docking results and literature information.

## Materials and Methods

### Datasets

#### PubChem BioAssay Database

For this study we selected nine datasets from the PubChem BioAssay database where targets are proteins except for one dataset where the target is cell-based. Although we have a special interest in protein targets, we choose a case that is cell-based to illustrate the generality of our method. It is worth noting that all the datasets we chose are based on the confirmatory assays and we avoided selection of primary assays based on recommendation of [25]. The datasets are based on the PubChem's BioAssay protocol, where assays can be referenced by a unique AID identifier. A single BioAssay reports experimental activity results for a set of chemical compounds over a specific biological target, which in most cases is a protein. So, a BioAssay dataset contains a list of chemical compounds with assigned labels, where label ‘+1’ indicates that the compound shows activity with the examined target, while ‘-1’ relates to inactive compounds. Table 1 provides a summary of the datasets used in the study. Eight of these datasets AID: 596, AID: 618, AID: 644, AID: 886, AID: 899, AID: 938, AID: 743042 and AID: 743288, were chosen to demonstrate different imbalance ratios (IR) between the active and inactive compound classes. The ninth one, called BenchSet, is a benchmark dataset that is obtained by merging three BioAssays, AID: 773, AID: 1006 and AID: 1379, as described previously by Li *et al*. [26]. In total, these datasets are composed of 11 BioAssays that represent 487,557 inactive and active interactions and offer a wide variety of class imbalance ratios ranging from 0.26% (i.e. high IR) to 48% (i.e. small IR), where IR is represented as ratio of the number of minority active cases to the number of majority inactive cases. For reporting performance over these datasets, 5-fold cross-validation setup is followed in all computational experiments. Given the large size of our experimental datasets (as shown in Table 1), 5-fold cross-validation for evaluation is a proper choice for computing a representative (i.e. non-biased) estimate [36, 37]. In order to avoid any potential bias, testing data is never used in the training process.

**Table 1:** Summary of experimental datasets including reference IDs in PubChem Database.

Dataset	Target Name (Target)	Type of interacting compounds	Minority Class Size	Majority Class Size	IR Ratio
BenchSet (AID: 773, AID: 1006 and AID: 1379)	Luciferase [Photuris pennsylvanica](Protein)	Inhibitors	487	184,154	1:377
AID 596	Microtubule-associated protein tau [Homo sapiens] (Protein)	Binders	1,391	66,726	1:48
AID 618	Matrix metalloproteinase 1, partial [Homo sapiens] (Protein)	Inhibitors	537	86,197	1:160
AID 644	Rho-associated protein kinase 2 [Homo sapiens] (Protein)	Inhibitors	67	139	1:2
AID 886	Chain B, The Structure Of Wild-Type Human Hadh2 (Protein)	Inhibitors	2,463	64,616	1:26
AID 899	Cytochrome P450 2C19 precursor [Homo sapiens] (Protein)	Inhibitors and Substrates	1,901	6,443	1:3
AID 938	Thyroid stimulating hormone receptor [Homo sapiens] (Protein)	Agonist Activators	1,794	60,806	1:34
AID 743042	Androgen receptor [Homo sapiens] (Protein)	Antagonist Activators	674	6,939	1:10
AID 743288	Hek293 cell line (Cell)	Binders	95	2,128	1:22
Total Interactions			**487,557**		

#### DrugBank database

The DrugBank database data (accessed on August, 2014) was downloaded from the website: http://www.drugbank.ca/ [35]. The initial database had about 6,800 drug entries including 1,491 FDA-approved drugs. We considered only the FDA-approved drug list to screen the model we developed for thyroid stimulating hormone receptor (TSHR).

### Generation of features

Generating and selecting a good subset of features is an important step in developing a well-performing classification model, and may also help in the cases of large class imbalance [31, 32]. A variety of feature sets of varying complexity have been compiled for virtual screening and prediction of biological activity [25, 38]. In this study, we used the combined set of fingerprint features from two major cheminformatics toolkits, RDKit [39] and OpenBabel [40], as well as features from PubChem fingerprints [24]. OpenBabel [40] was specifically used to generate different SMARTS patterns and 3D spectrophore descriptors. In addition, several basic chemical descriptors, such as the molecular weight, number of H-acceptors and donors, and Log-P, were calculated. The finally generated set of features contained 2,940 features. A detailed description of all the features used in the study, as well as the ones we selected, is provided in S1 Text. This, to the best of our knowledge, is the largest set of features compiled for use in prediction of chemical activity status from HTS assays.

### Feature selection (FS)

A large set of compiled features, as described in the previous section, leads to generating information of different level of redundancy, as well as introduces features that may not be relevant to the types of biological activity of chemicals as observed in particular assays. A good FS method should be able to remove a lot of such redundant or irrelevant information [41]. FS methods, in general, can be categorized into: the filters, the wrappers, and those based on the embedded FS models [32, 42]. In this study, the wrapper FS model of DWFS tool [43], which selects features so as to maximize the performance of a classifier, is applied. The default setup of DWFS tool was used for FS experiments. As an illustration, an analysis of the effect of FS on the classification performance for one of the datasets can be found in S2 Text.

### Classifiers

Six widely used classifiers are applied as a basis for comparing different solutions of the class imbalance problem for activity testing in PubChem assays. These include support vector machines [44, 45] (SVM) with linear and radial basis function (RBF) kernels, K-nearest neighbors (KNN; K = 3) [46], Linear Discriminant Analysis (LDA) [47], Naïve Bayes Classifier (NBC) [48] and Random Forests (RF) [49]. For SVM, LIBSVM [50] implementation was used for building the different SVM models. The default cost parameter as well as RBF kernel widths were used.

### Performance evaluation

Performance of all methods referred to in the results section is obtained form a 5-fold cross-validation. The testing fold was never used in the training phase. Since we performed 5-fold cross-validation, with six classifiers and five class imbalance solutions, we performed 150 (5 folds × 6 classifiers × 5 solutions = 150) experiments for each dataset and 1,350 in total for all nine datasets. We report the average performance over the 5-folds of every dataset, as well as the standard deviation. In addition, we perform significance analysis between the methods using one-way analysis of variance (ANOVA). In cases where there is a significant difference between the methods, we further apply the well-known pair-wise Tukey mean-mean multiple comparison (MCC) to determine which pairs are significantly different, while simultaneously examining all methods [see S1 Table]. Giving the characteristics of this problem and the nature of having highly imbalanced classes, we provide results over many performance metrics to gain a generic view of the performances of different solutions. Let TP be the number of true positives, FP the number of false positives, TN the number of true negatives and FN the number of false negatives. The results in this study are reported based on Eqs (1–7).

sensitivity=TP/(TP+FN) (1)

specificity=TN/(TN+FP (2)

precision=TP/(TP+FP) (3)

$$GMean=\sqrt{sensitivity\times specificity}$$ (4)

$$F_{1}Score=2\times \frac{precision\times sensitivity}{precision+sensitivity}$$ (5)

$$F_{0.5}Score=1.25\times \frac{precision\times sensitivity}{0.25\times precision+sensitivity}$$ (6)

ROCAUC=Areaunderthereceiveroperatingcharacteristiccurve (7)

The predictions of a classifier for a HTS dataset should result in high precision in order for the set of predicted active compounds to contain as few FP predictions as possible. The number of FPs is a crucial factor in measuring the reliability of predictions as minimizing it leads to increased chances of successful follow up experiments.

F_1_Score [9] is a summary metric that computes the weighted average of precision and sensitivity. It is also known as balanced F-Score since it balances both precision and sensitivity equally. F_0.5_Score [11, 51, 52] is another summary metric that weights precision twice as much as sensitivity. Given that intention to use the classifier for computational screening of millions of compounds, sensitivity is of less importance than precision. A conservative sensitivity rate with higher precision will still lead to large number of accurate new findings when screening a large number of candidates in the context of HTS. Thus, we give preference to precision and F_0.5_Score as more indicative performance measures in such scenario. We consider also in the results section, discussion over sensitivity and F_1_Score. For other metrics like specificity and ROC AUC scores are reported in details in S2 Table.

### Methods

#### Data preprocessing for class imbalance case

HTS experiments are usually characterized by only a small number of active chemical compounds obtained after screening a big compound set. This nature of imbalanced distributions of the active and inactive compound classes may lead to a degraded classification performance that should be addressed. The class imbalance problem is one of the challenging tasks that received a lot of attention [53–55]. There exists a wide variety of state-of-the-art solutions of the class imbalance problem, which can be categorized abstractly into algorithmic and data-based ones [9]. In our study we consider the following approaches: majority random under-sampling (RU), synthetic minority oversampling technique (SMOTE) [56], granular SVMs for under-sampling (GSVM-RU) [57, 58], majority weighted minority over-sampling technique (MWMOTE) [59] and our precision-aware proposed method DRAMOTE. Further details about the existing methods are provided in S3 Text.

#### DRAMOTE: our proposed solution

There are certain limitations with the existing solutions for data preprocessing in the case of class imbalance. Methods like RU and SMOTE apply sampling procedures to data without considering the effect of sampling on the classification performance. These methods are independent of any feedback from the classifier and may affect the performance only to a certain limit. In other words, these methods do not provide a mechanism to have a control over precision or other performance metrics. Other algorithms like GSVM-RU, take into account the performance of the classifier, but are limited to a specific classifier, e.g. GSVM-RU is limited to SVM and cannot be applied to other classifiers. MWMOTE needs more parameters for selecting an informative set of minority samples and is limited to optimize the performance over nearest neighbor type of classifiers. We propose here a novel method motivated by ideas from active learning (AL) (for more details about AL see S3 Text). The method is based on establishing a feedback loop with the classifier to highlight points contributing most to its precision (other performance metrics can be used).

**Fig 1:**
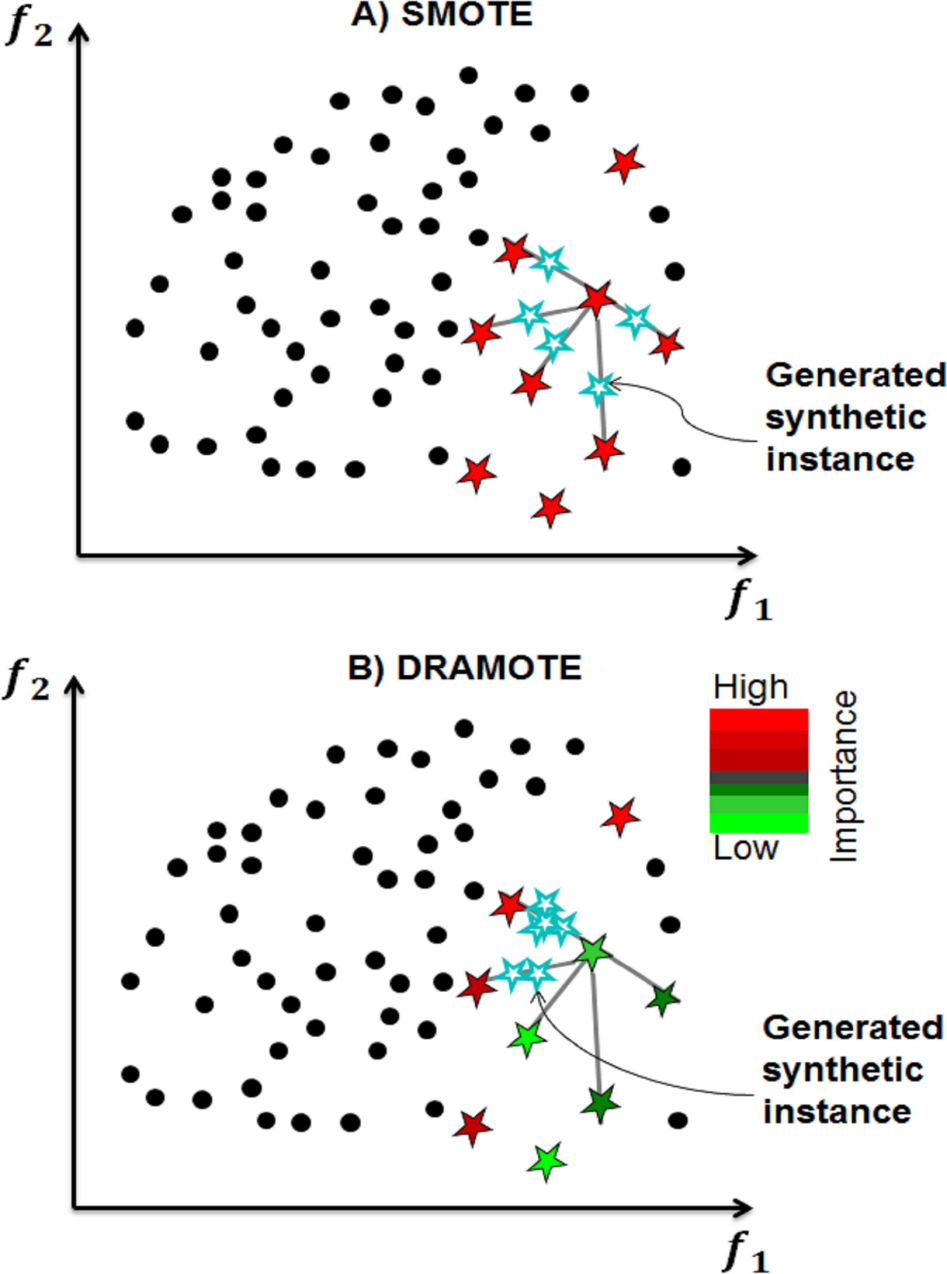
Illustration of generating synthetic instances. A) SMOTE generates the light blue samples by interpolation between a randomly chosen minority sample and k-nearest neighbors. B) DRAMOTE generates the light blue samples by choosing a minority sample based on its importance (i.e. contribution to precision) and the direction towards a safe region. A minority sample (red colored) that is very close to the majority negatives circles will be probably misclassified as a negative one and hence, it should get more support compared to the green colored minority samples. Once a minority sample is chosen, another point needs to be chosen for interpolation. The direction of interpolation can be controlled by choosing a nearest neighbor which is not overlapping with the negative class. This, in turn, helps in providing support for the red colored point while not harming the classifier performance in its surrounding region.

Fig 1 gives a simplified illustration of DRAMOTE, where minority samples are colored based on how informative they are towards minimizing the false positives and this can be compared with SMOTE which does not differentiate between usefulness levels of minority samples. Another major difference between DRAMOTE and SMOTE is choosing the direction for synthetically generating the new samples as illustrated by the blue points that highlight this difference in parts A and B of the Fig 1. Further details about DRAMOTE including mathematical equations and pseudocode details are provided in S3 Text.

## Results and Discussion

### Performance Comparison

We made a number of experiments to evaluate performance of the methods we used. The results are provided in Table 2 over the analyzed BioAssays. Table 2 shows the 5-fold cross-validation comparison results between the different class imbalance solutions. The summary scores in Table 2 are based on averaging the performance over six types of classifiers for each dataset. Another summary results with statistical significance analysis including *p*-values can be found in S1 Table and the detailed results including other performance metrics can be found in S2 Table.

**Table 2:** Comparison of the data preprocessing methods. Larger standard deviation values are the result of averaging over different types of classifiers in this summary table.

Dataset	Method	Sensitivity %	Precision %	F_1_ Score %	F_0.5_ Score %
**BenchSet**	RU	85.67 (±2.5)	1.07 (±0.29)	2.93 (±0.56)	1.33 (±0.35)
	GSVM-RU	68.53 (±6)	2.73 (±2.05)	5.13 (±3.7)	3.36 (±2.49)
	SMOTE	62.79 (±15.32)	10.44 (±16.11)	**12.44 (±13.64)**	10.87 (±15.17)
	MWMOTE	69.49 (±13.18)	4.9 (±6.7)	7.87 (±9.08)	5.75 (±7.52)
	DRAMOTE	58.14 (±19.2)	**13.35 (±22.66)**	11.62 (±11.42)	**11.68 (±16.57)**
	[26]	**88.46**	5	NA^a^	NA^a^
**AID 596**	RU	75.9 (±3.04)	5.3 (±1.17)	9.89 (±2.07)	6.51 (±1.41)
	GSVM-RU	**82.78 (±7.93)**	4.56 (±2.78)	8.46 (±4.78)	5.59 (±3.34)
	SMOTE	64.02 (±13.8)	10.9 (±8.95)	16.38 (±9.28)	12.47 (±9.16)
	MWMOTE	62.1 (±14.3)	10.8 (±9.2)	16.11 (±9.34)	12.32 (±9.37)
	DRAMOTE	42.9 (±13.52)	**18.41 (±17.81)**	**19.43 (±9.63)**	**18 (±13.61)**
**AID 618**	RU	72.54 (±3.41)	1.38 (±0.31)	2.7 (±0.59)	1.71 (±0.38)
	GSVM-RU	**52.42 (±11.76)**	2.64 (±1.48)	4.89 (±2.59)	3.24 (±1.79)
	SMOTE	43.01 (±17.87)	10.07 (±12.36)	10.93 (±8.36)	10.01 (±10.42)
	MWMOTE	42.34 (±18.53)	10.31 (±12.72)	**11.38 (±8.12)**	10.24 (±10.49)
	DRAMOTE	29.69 (±15.26)	**12.78 (±15.61)**	9.73 (±6.09)	**10.58 (±10.46)**
**AID 644**	RU	50.29 (±4.46)	35.08 (±2.56)	40.32 (±3.1)	37.3 (±2.49)
	GSVM-RU	**71.28 (±12.76)**	36.02 (±2.51)	**46.62 (±3.23)**	39.84 (±2.48)
	SMOTE	47.3 (±14.1)	41.78 (±7.23)	40.95 (±3.21)	41.65 (±3.72)
	MWMOTE	47.37 (±12.37)	42.22 (±6.68)	41.99 (±3.24)	**42.4 (±4.42)**
	DRAMOTE	40.09 (±8.51)	**43.14 (±9.88)**	38.84 (±1.64)	41.49 (±5.79)
**AID 886**	RU	**99.54 (±0.31)**	67.65 (±2.55)	80.52 (±1.75)	72.27 (±2.31)
	GSVM-RU	99.25 (±0.97)	54.51 (±26.52)	65.87 (±29.63)	58.53 (±27.76)
	SMOTE	96.94 (±4.11)	75.2 (±4.92)	**84.43 (±2.51)**	**78.65 (±3.99)**
	MWMOTE	97.03 (±3.27)	74.32 (±4.81)	83.98 (±2.75)	77.9 (±4.06)
	DRAMOTE	94.38 (±8.1)	**75.69 (±6.05)**	83.55 (±3.72)	78.56 (±4.17)
**AID 899**	RU	77.65 (±3.43)	45.96 (±7.07)	57.33 (±5.46)	49.89 (±6.7)
	GSVM-RU	**97.29 (±3.22)**	25.82 (±2.6)	40.69 (±2.84)	30.25 (±2.76)
	SMOTE	70.44 (±8.14)	53.52 (±14.02)	**59.07 (±6.9)**	**55.32 (±11.22)**
	MWMOTE	70.5 (±8.48)	52.61 (±13.66)	58.55 (±6.55)	54.55 (±10.83)
	DRAMOTE	64.51 (±8.01)	**53.61 (±14.43)**	56.73 (±5.38)	54.47 (±10.69)
**AID 938**	RU	**99.42 (±0.41)**	66.17 (±2)	79.4 (±1.45)	37.3 (±2.49)
	GSVM-RU	99.16 (±0.5)	45.85 (±17.01)	56.79 (±17.22)	49.64 (±24.09)
	SMOTE	91.86 (±0.9)	80.05 (±1.8)	84 (±1.34)	81.94 (±11.11)
	MWMOTE	94.49 (±8.2)	70.7 (±8)	80.74 (±1.9)	74.41 (±6.24)
	DRAMOTE	91.39 (±4)	**81.02 (±2.03)**	**84.32 (±3)**	**82.66 (±10.73)**
**AID 743042**	RU	71.34 (±7.44)	17.22 (±2.83)	27.66 (±4)	20.28 (±3.21)
	GSVM-RU	**93.21 (±7.7)**	11.11 (±0.65)	19.81 (±0.9)	13.47 (±0.74)
	SMOTE	33.38 (±16.32)	36.99 (±21.61)	27.71 (±8.52)	29.84 (±10.97)
	MWMOTE	35.52 (±14.9)	36.54 (±18.4)	30.56 (±7.01)	32.18 (±9.78)
	DRAMOTE	35.38 (±14.13)	**38.69 (±20.85)**	**30.76 (±6.04)**	**33.03 (±10.23)**
**AID 743288**	RU	68.09 (±5.53)	8.38 (±1.07)	14.89 (±1.77)	10.16 (±1.27)
	GSVM-RU	**86.33 (±6.49)**	5.76 (±0.4)	10.78 (±0.68)	7.08 (±0.48)
	SMOTE	25.74 (±18.34)	26.99 (±23.95)	24.56 (±6.5)	24.05 (±10.15)
	MWMOTE	23.8 (±17.4)	33.02 (±21.18)	23.75 (±9.67)	25.78 (±10.32)
	DRAMOTE	27.88 (±14.66)	**34.13 (±20.58)**	**27.03 (±6.97)**	**29.02 (±10.42)**

^a^ NA indicates that a particular measure was not reported in the referenced work

In Table 2, we consider examining more closely sensitivity, precision, F_1_Score and F_0.5_Score[51] for evaluating classification results on HTS types of data. These performance metrics shall better reflect the impact over the data with imbalanced classes [11], but other performance metrics like specificity, GMean, and ROC-AUC are also included in S2 Table.

To see where a particular solution stands among all the remaining ones, we also ranked the performance of each of the methods for every classifier based on the F_1_Score. We then averaged the rank position for each of the methods. The method with the lowest score is the best performing. We provide in Table 3 the rank position and averaged rank position for each of the methods. Table 3 clearly demonstrates that overall DRAMOTE and SMOTE were the best performing method in terms of F_1_Score.

**Table 3:** Ranking of methods based on F_1_Score for every classifier.

Classifier	RU	GSVM-RU	SMOTE	MWMOTE	DRAMOTE
SVM-L	3	5	1	4	2
SVM-R	4	5	2	3	1
KNN	3	5	2	4	1
LDA	4	5	2	3	1
NBC	1	4	3	5	2
RF	4	5	1	3	2
Average	3.17	4.83	**1.83**	3.67	**1.50**

SMOTE, MWMOTE and DRAMOTE are all methods that generate synthetic data with exactly the same number of new over-sampling points. However, DRAMOTE gives preference to generating points contributing more to the precision of a particular classifier. Results of Table 2 confirm this in all nine datasets, based on the fact that DRAMOTE achieves the highest precision with an improvement of a factor of 2.4 relative to precision of every other method, on average. In three out of nine cases SMOTE achieves the best in terms of F_1_Score and in four cases the second best. For four out of nine datasets, DRAMOTE (compared to other solutions) achieved the highest F_1_Score, while appeared the best in terms of F_0.5_Score for six out of the nine datasets.

Compared to GSVM-RU that was reported as an effective method for PubChem BioAssays [26], DRAMOTE shows a significant improvement in precision for five out of nine datasets, while sacrifices sensitivity significantly as compared to GSVM-RU in only three cases [see S1 Table].

### Compounds Interacting with Thyroid Stimulating Hormone Receptor (TSHR)

This section describes a case study for prediction of activity status of FDA drugs with TSHR protein. TSHR is a key protein in the control of thyroid function and belongs to the superfamily of G-protein-coupled receptors (GPCRs) [60]. Thyroid stimulating hormone (TSH) is the main factor responsible for regulating both differentiated function and growth of thyroid follicular epithelial cells [61]. Specifically, BioAssay AID 938 in PubChem database is an assay for finding agonists of the TSHR, which is based on stimulation of cAMP production that causes the cyclic nucleotide gated ion channel (CNG) to open to control for compounds signaling through endogenous receptors and other targets of HEK 293 cells.

The biochemical relevance of the 10 top ranked predictions by DRAMOTE was further indirectly supported by *in silico* docking results and literature. For this case study, we use the previous results to select a proper solution to preprocess the data and then, build a system based on ensemble of all examined classifiers. Following is the discussion of the results related to this carefully tuned system.

#### Computational prediction and support

The application of DRAMOTE to the TSHR dataset (AID 938) resulted in precision of 81.02% and sensitivity of 91.39%. After building an ensemble of all six trained classifiers, the performance improved by maintaining similar level of precision (~81%) but with a sensitivity of 98.84% (i.e. more than 7% increase in sensitivity).

We investigated the potential interaction of approved drugs from the DrugBank database [35] over TSHR. The ensemble of classifiers trained using BioAssay (AID 938) as its training set, is used to computationally screen approved drugs extracted from DrugBank. We report the top 10 predictions as candidate drugs with strong potential to be interact with TSHR. Table 4 provides a brief description of each drug and highlights their ranking score based on the ensemble system. The drugs, also, docked to TSHR are shown with their corresponding names and structures in S1 Fig.

**Table 4:** Top 10 ranked predictions by DRAMOTE for BioAssay 938 with TSHR protein target.

Rank	DrugBank ID	Drug Name	Description	Ensemble System Score
1	DB00904	Ondansetron	Treatment of nausea and vomiting caused by cytotoxic chemotherapy drugs	0.98
2	DB00962	Zaleplon	Sedative/hypnotic, mainly used for insomnia	0.97
3	DB01349	Tasosartan	Treat patients with essential hypertension	0.966
4	DB00405	Dexbrompheniramine	Treat allergic conditions such as hay fever or urticaria	0.96
5	DB01261	Sitagliptin	Control of type 2 diabetes mellitus	0.958
6	DB06439	Tyloxapol	Non-ionic detergent often used as a surfactant	0.957
7	DB00889	Granisetron	Antiemetic and antinauseant for cancer chemotherapy patients	0.954
8	DB01342	Forasartan	Used alone or with other antihypertensive agents to treat hypertension	0.953
9	DB00748	Carbinoxamine	First generation antihistamine that competes with free histamine for binding at HA-receptor sites	0.95
10	DB06267	Udenafil	Treat erectile dysfunction	0.945

**Fig 2:**
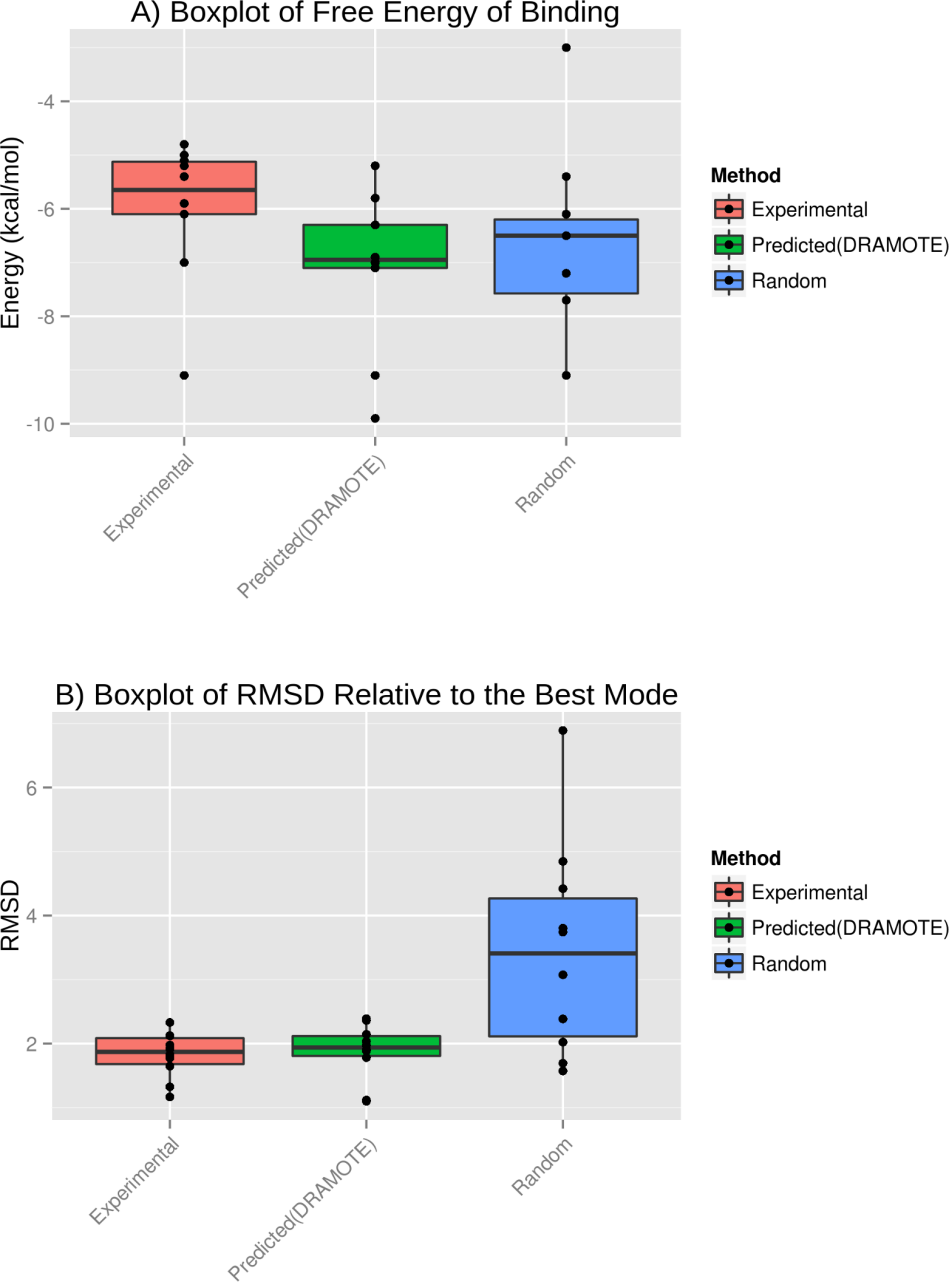
Boxplot over free energy of binding and RMSD values for experimental, random and DRAMOTE docking results. The random set is based on choosing 10 random drugs from approved drugs list in DrugBank database. The experimental set includes the top 10 drugs as listed in the original BioAssay AID 938 of PubChem database.

Docking simulations can indirectly support the previous top findings in our data-driven approach. While docking simulations are prone to false positives, the presence of consistent levels in binding values between our findings and the top experimentally ranked interactions reported in AID 938 gives more confidence about our suggested candidates having active interaction status with TSHR. Fig 2 illustrates comparison of the docking scores of the top 10 predictions suggested by DRAMOTE, against two other sets of docking experiments we used as references for evaluation. The two sets of docking experiments include the actual top 10 experimental interactions as ranked and reported in PubChem database for AID 938 and another set (Random set) of 10 randomly selected drugs from the approved list of DrugBank [38]. In Fig 2, the listed energies correspond to the lowest predicted binding energy and they are given in kcal/mol as calculated by AutoDock Vina [62]. Part B of Fig 2 provides the root mean squared distance (RMSD) values of the best poses of each drug compound docked to an activation site in TSHR. While difference is not apparent with regard to the free energy values between all the three sets of docking experiments, the RMSD levels achieved by docking predictions using DRAMOTE are very similar to the levels achieved by the experimentally validated ones as compared to the Random set. Detailed docking procedure and scores are provided in S4 Text.

A literature review of our top predictions points out that **Tasosartan** (third ranked prediction) and **Forasartan** (eighth ranked prediction) are both angiotensin II receptor antagonist. These drugs are used to treat hypertension [63] and are known to block the renin-angiotensin system thereby protecting the kidney from damage caused by increased kidney blood pressure [64]. Several studies have demonstrated a positive correlation between high blood pressure and the concentration of thyroid stimulating hormone [65–67]. Literature review for remaining top ranked drugs can be found in S5 Text. These findings strengthen our proposition that the proposed top 10 predictions could be candidate drugs for interacting with TSHR. In order to show that DRAMOTE can be used for drugs for different diseases other than those related to TSHR, an additional top ranked list is included in S6 Text for 17beta-Hydroxysteroid Dehydrogenase Type 10 (17β-HSD10) as the protein target in AID 886 assay. The expression level of this protein is elevated in the brains of Alzheimer’s disease patients [68]. Thus, the predicted/suggested drugs (S6 Text) could serve as potential drugs for Alzheimer’s disease aimed to inhibit expression of 17β-HSD10 since AID 886 assay is testing inhibition of 17β-HSD10.

## Conclusions

In this study, we extensively compare several state-of-the-art methods that handle class imbalance problem based on advanced sampling techniques. The results based on approximately 500,000 interactions suggest that DRAMOTE can be used for developing robust virtual screening models to recognize candidate chemical compounds for potential activity with specific molecular targets in specific assays. Moreover, we applied DRAMOTE to screen for drugs likely to interact with the TSHR as a case study and we presented the top 10 drugs that potentially interact with TSHR along with indirect supporting evidence of their validity from literature and simulated 3D docking.

## Supporting Information

S1 Fig Docking output results for Carbinoxamine, Granisetron, Ondansetron, Zalepon, Sitagliptin, Forasartan, Tasosartan, Udenafil, Tyloxapol with TSHR. The orange color highlights the top docking results of a drug binding to the chosen activation site.
(TIFF)

S1 Table Extended comparison of existing and proposed methods including an analysis of significance of difference between the reported performance metrics. (DOCX)

S2 Table Detailed comparison results for each dataset. Mean and variance of 5-fold cross-validation performance scores are displayed for each method and for each used classifiers.
(DOCX)

S1 Text Summary description of features generated for chemical compounds. The file also includes most of the features we selected after applying variable selection over the originals set of generated features.
(DOCX)

S2 Text Effect of feature selection results on classification performance. (DOCX)

S3 Text Details about the existing state-of-the-art solutions used in the study and their input parameters. The file includes also all information about DRAMOTE and its procedure.
(DOCX)

S4 Text Detailed docking scores including the set of random selected drugs and description of the docking procedure. (DOCX)

S5 Text Extended literature review of the top predicted FDA drugs for the TSHR in humans. (DOCX)

S6 Text A list of the top ranked prediction by DRAMOTE for potential drugs interacting with 17β-HSD10 in humans. (DOCX)
