# Supplementary figures and images for: Mining Chemical Activity Status from High-Throughput Screening Assays
Source: PLoS One. 2015 Dec 14;10(12):e0144426. doi: 10.1371/journal.pone.0144426 (PMC4682830; doi:10.1371/journal.pone.0144426)

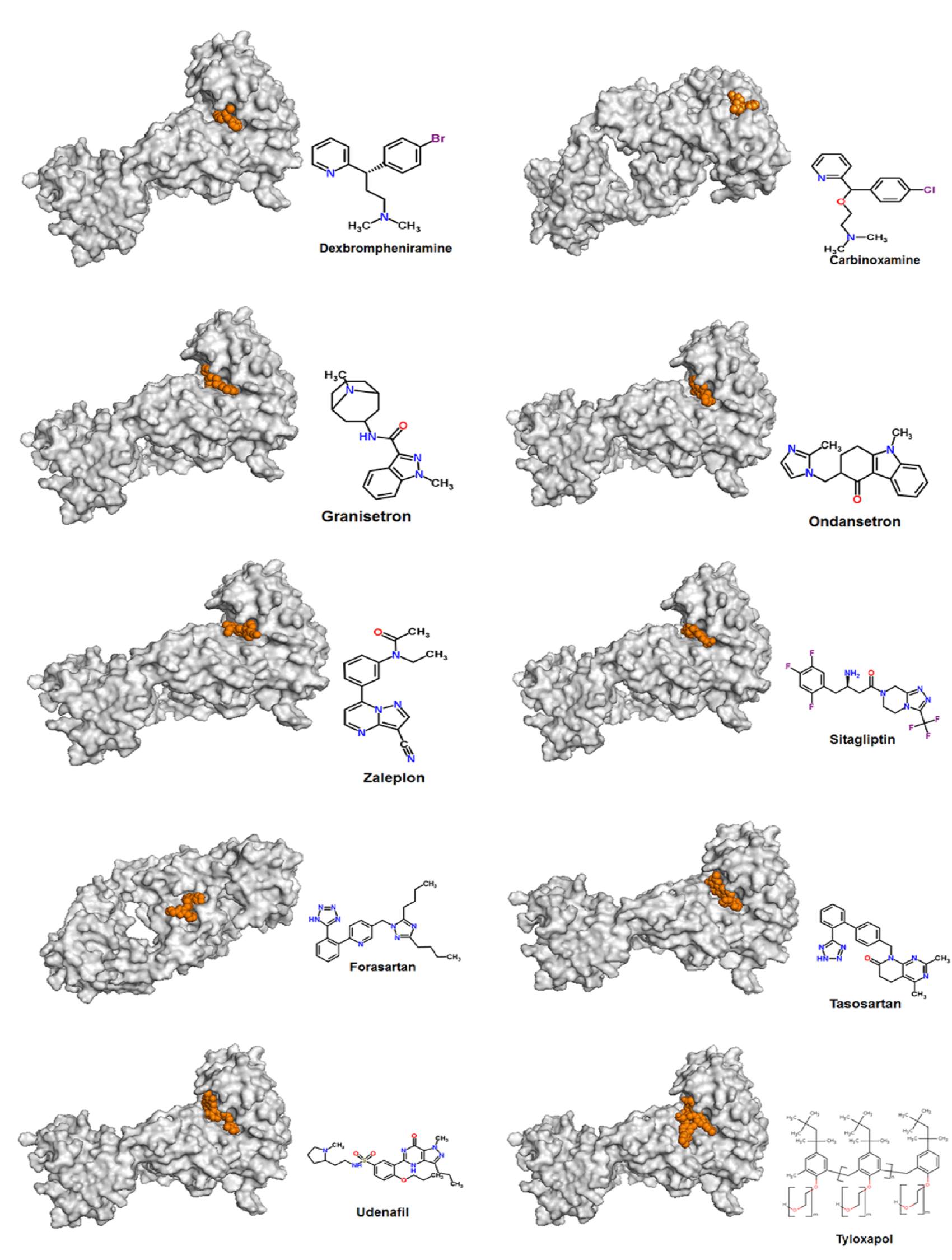

Supplement: S1 Fig — The orange color highlights the top docking results of a drug binding to the chosen activation site. (TIFF) [file pone.0144426.s001.tiff]
