# Supplementary material for: Mining Chemical Activity Status from High-Throughput Screening Assays
Source: PLoS One. 2015 Dec 14;10(12):e0144426. doi: 10.1371/journal.pone.0144426 (PMC4682830; doi:10.1371/journal.pone.0144426)
Supplement: S1 Table — (DOCX) [file pone.0144426.s002.docx]

# Mining chemical activity status in high-throughput screening assays

*Othman Soufan^1^, Wail Ba-alawi^1^, Moataz Afeef^1^, Magbubah Essack^1^****,*** *Valentin Rodionov^2^,* *Panos Kalnis^3^ and Vladimir B. Bajic^1,*^*

^1^King Abdullah University of Science and Technology (KAUST), Computational Bioscience Research Center (CBRC), Thuwal 23955-6900, Saudi Arabia. ^2^King Abdullah University of Science and Technology (KAUST), KAUST Catalysis Center (KCC), Thuwal 23955-6900, Saudi Arabia. ^3^King Abdullah University of Science and Technology (KAUST), Infocloud Group, Computer, Electrical and Mathematical Sciences and Engineering Division (CEMSE), Thuwal 23955-6900, Saudi Arabia.

# Supporting Information Table 1

## **An extended comparison of existing and proposed methods including an analysis of significance of difference between the reported performance metrics.**

Table 1: Comparison of existing and proposed methods: analyzing the effect of significance of performance metrics

| **Dataset** | **Method** | **Sensitivity %** | **Precision %** |
| --- | --- | --- | --- |
| **BenchSet** | RU (1) | **85.67 (±2.5)** | 1.07 (±0.29) |
|  | GSVM-RU (2) | 68.53 (±6) | 2.73 (±2.05) |
|  | SMOTE (3) | 62.79 (±15.32) | 10.44 (±16.11) |
|  | MWMOTE (4) | 69.49 (±13.18) | 4.9 (±6.7) |
|  | DRAMOTE (5) | 58.14 (±19.2) | **13.35 (±22.66)** |
|  | [[16](#_ENREF_16)] | 88.46 | 5 |
|  | P-value | 0.0244 | 0.5464 |
|  | Tukey MMC | Methods (1) & (5) | ____ |
|  |  |  |  |
| **AID 596** | RU (1) | 75.9 (±3.04) | 5.3 (±1.17) |
|  | GSVM-RU (2) | **82.78 (±7.93)** | 4.56 (±2.78) |
|  | SMOTE (3) | 64.02 (±13.8) | 10.9 (±8.95) |
|  | MWMOTE (4) | 62.1 (±14.3) | 10.8 (±9.2) |
|  | DRAMOTE (5) | 42.9 (±13.52) | **18.41 (±17.81)** |
|  | P-Value | 6.8325e-05 | 0.2196 |
|  | Tukey MMC | Methods (1) & (5) Methods (2) & (5) Methods (3) & (5) | ____ |
|  |  |  |  |
| **AID 618** | RU (1) | 72.54 (±3.41) | 1.38 (±0.31) |
|  | GSVM-RU (2) | **52.42 (±11.76)** | 2.64 (±1.48) |
|  | SMOTE (3) | 43.01 (±17.87) | 10.07 (±12.36) |
|  | MWMOTE (4) | 42.34 (±18.53) | 10.31 (±12.72) |
|  | DRAMOTE (5) | 29.69 (±15.26) | **12.78 (±15.61)** |
|  | P-Value | 3.5600e-04 | 0.3738 |
|  | Tukey MMC | Methods (1) & (3) Methods (1) & (4) Methods (1) & (5) | ____ |
|  |  |  |  |
| **AID 644** | RU (1) | 50.29 (±4.46) | 35.08 (±2.56) |
|  | GSVM-RU (2) | **71.28 (±12.76)** | 36.02 (±2.51) |
|  | SMOTE (3) | 47.3 (±14.1) | 41.78 (±7.23) |
|  | MWMOTE (4) | 47.37 (±12.37) | 42.22 (±6.68) |
|  | DRAMOTE (5) | 40.09 (±8.51) | **43.14 (±9.88)** |
|  | P-Value | 0.0036 | 0.2058 |
|  | Tukey MMC | Methods (2) & (3) Methods (2) & (4) Methods (2) & (5) | ____ |
|  |  |  |  |
| **AID 886** | RU (1) | **99.54 (±0.31)** | 67.65 (±2.55) |
|  | GSVM-RU (2) | 99.25 (±0.97) | 54.51 (±26.52) |
|  | SMOTE (3) | 96.94 (±4.11) | 75.2 (±4.92) |
|  | MWMOTE (4) | 97.03 (±3.27) | 74.32 (±4.81) |
|  | DRAMOTE (5) | 94.38 (±8.1) | **75.69 (±6.05)** |
|  | P-Value | 0.3455 | 0.0242 |
|  | Tukey MMC | ____ | Methods (2) & (3) Methods (2) & (5) |
|  |  |  |  |
| **AID 899** | RU (1) | 77.65 (±3.43) | 45.96 (±7.07) |
|  | GSVM-RU (2) | **97.29 (±3.22)** | 25.82 (±2.6) |
|  | SMOTE (3) | 70.44 (±8.14) | 53.52 (±14.02) |
|  | MWMOTE (4) | 70.5 (±8.48) | 52.61 (±13.66) |
|  | DRAMOTE (5) | 64.51 (±8.01) | **53.61 (±14.43)** |
|  | P-Value | 1.7539e-07 | 0.0017 |
|  | Tukey MMC | Methods (1) & (2) Methods (1) & (6)  Methods (2) & (3)  Methods (2) & (4)  Methods (2) & (5) | Methods (2) & (3)  Methods (2) & (4)  Methods (2) & (5) |
|  |  |  |  |
| **AID 938** | RU (1) | **99.42 (±0.41)** | 66.17 (±2) |
|  | GSVM-RU (2) | 99.16 (±0.5) | 45.85 (±17.01) |
|  | SMOTE (3) | 91.86 (±0.9) | 80.05 (±1.8) |
|  | MWMOTE (4) | 94.49 (±8.2) | 70.7 (±8) |
|  | DRAMOTE (5) | 91.39 (±4) | **81.02 (±2.03)** |
|  | P-Value | 0.3008 | 1.2445e-09 |
|  | Tukey MMC | ____ | Methods (1) & (2) Methods (1) & (3)  Methods (1) & (4)  Methods (1) & (5)  Methods (2) & (3)  Methods (2) & (4)  Methods (2) & (5) |
|  |  |  |  |
| **AID 743042** | RU (1) | 71.34 (±7.44) | 17.22 (±2.83) |
|  | GSVM-RU (2) | **93.21 (±7.7)** | 11.11 (±0.65) |
|  | SMOTE (3) | 33.38 (±16.32) | 36.99 (±21.61) |
|  | MWMOTE (4) | 35.52 (±14.9) | 36.54 (±18.4) |
|  | DRAMOTE (5) | 35.38 (±14.13) | **38.69 (±20.85)** |
|  | P-Value | 2.7818e-12 | 5.0946e-06 |
|  | Tukey MMC | Methods (1) & (2)  Methods (1) & (3)  Methods (1) & (4)  Methods (1) & (5)  Methods (2) & (3)  Methods (3) & (4)  Methods (3) & (5) | Methods (1) & (4)  Methods (1) & (5)  Methods (2) & (3)  Methods (2) & (4)  Methods (2) & (5) |
|  |  |  |  |
| **AID 743288** | RU (1) | 68.09 (±5.53) | 8.38 (±1.07) |
|  | GSVM-RU (2) | **86.33 (±6.49)** | 5.76 (±0.4) |
|  | SMOTE (3) | 25.74 (±18.34) | 26.99 (±23.95) |
|  | MWMOTE (4) | 23.8 (±17.4) | 33.02 (±21.18) |
|  | DRAMOTE (5) | 27.88 (±14.66) | **34.13 (±20.58)** |
|  | P-Value | 8.6842e-15 | 5.8945e-04 |
|  | Tukey MMC | Methods (1) & (3)  Methods (1) & (4)  Methods (1) & (5)  Methods (2) & (3)  Methods (3) & (4)  Methods (3) & (5) | Methods (1) & (4)  Methods (1) & (5)  Methods (2) & (4)  Methods (2) & (5) |

*p*-Values are for testing whether there is a significant difference between the methods (a.k.a. groups) and in cases where such significance is present, we use Tukey mean-mean multiple comparison to carry a pair-wise significance test. This allows seeing which methods are achieving sensitivity or precision scores significantly different from each other and we indicate this by listing a number representing the method e.g. (2) for GSVM-RU.
