# Supplementary material for: Mining Chemical Activity Status from High-Throughput Screening Assays
Source: PLoS One. 2015 Dec 14;10(12):e0144426. doi: 10.1371/journal.pone.0144426 (PMC4682830; doi:10.1371/journal.pone.0144426)
Supplement: S2 Table — Mean and variance of 5-fold cross-validation performance scores are displayed for each method and for each used classifiers. (DOCX) [file pone.0144426.s003.docx]

# Mining chemical activity status in high-throughput screening assays

*Othman Soufan^1^, Wail Ba-alawi^1^, Moataz Afeef^1^, Magbubah Essack^1^****,*** *Valentin Rodionov^2^,* *Panos Kalnis^3^ and Vladimir B. Bajic^1,*^*

^1^King Abdullah University of Science and Technology (KAUST), Computational Bioscience Research Center (CBRC), Thuwal 23955-6900, Saudi Arabia. ^2^King Abdullah University of Science and Technology (KAUST), KAUST Catalysis Center (KCC), Thuwal 23955-6900, Saudi Arabia. ^3^King Abdullah University of Science and Technology (KAUST), Infocloud Group, Computer, Electrical and Mathematical Sciences and Engineering Division (CEMSE), Thuwal 23955-6900, Saudi Arabia.

# Supporting Information Table 2

**Detailed comparison results for each dataset. Mean and variance of 5-fold cross-validation performance scores are displayed for each method and for each used classifiers.**

|  | **BenchSet – No preprocessing** | | | |  |  |  |
| --- | --- | --- | --- | --- | --- | --- | --- |
| Classifier | Sensitivity | Specificity | Precision | G-Mean | | F1-Measure | ROC-AUC |
| SVM-L-5-folds-mean | 0.17% | 100.00% | 0.00% | 1.87% | | 0.00% | 11.22% |
| SVM-RBF-5-folds-mean | 0.00% | 100.00% | 0.00% | 0.00% | | 0.00% | 9.37% |
| KNN(3k)-5-folds-mean | 14.79% | 99.95% | 45.96% | 38.37% | | 22.20% | 70.04% |
| LDA-5-folds-mean | 27.21% | 99.67% | 18.11% | 51.98% | | 21.69% | 92.74% |
| NBC-5-folds-mean | 81.90% | 75.17% | 0.87% | 78.45% | | 1.73% | 85.53% |
| RF-5-folds-mean | 4.06% | 100.00% | 93.33% | 19.41% | | 7.70% | 85.07% |
| SVM-L-5-folds-std | 0.39% | 0.00% | 0.00% | 4.17% | | 0.00% | 7.69% |
| SVM-RBF-5-folds-std | 0.00% | 0.00% | 0.00% | 0.00% | | 0.00% | 1.44% |
| KNN(3k)-5-folds-std | 2.02% | 0.02% | 9.69% | 2.73% | | 2.96% | 1.58% |
| LDA-5-folds-std | 3.69% | 0.04% | 1.91% | 3.51% | | 2.24% | 1.07% |
| NBC-5-folds-std | 3.38% | 0.40% | 0.12% | 1.50% | | 0.23% | 0.80% |
| RF-5-folds-std | 2.24% | 0.00% | 14.91% | 6.08% | | 4.14% | 2.56% |

|  | **BenchSet – Random Undersampling** | | | |  |  |  |
| --- | --- | --- | --- | --- | --- | --- | --- |
| Classifier | Sensitivity | Specificity | Precision | G-Mean | | F1-Measure | ROC-AUC |
| SVM-L-5-folds-mean | 81.85% | 80.44% | 1.11% | 81.10% | | 2.18% | 89.18% |
| SVM-RBF-5-folds-mean | 85.27% | 77.59% | 1.01% | 81.32% | | 2.00% | 90.87% |
| KNN(3k)-5-folds-mean | 86.49% | 69.50% | 0.75% | 77.52% | | 1.49% | 84.63% |
| LDA-5-folds-mean | 89.60% | 81.37% | 1.27% | 85.37% | | 2.50% | 92.85% |
| NBC-5-folds-mean | 85.81% | 70.78% | 0.78% | 77.92% | | 1.54% | 84.46% |
| RF-5-folds-mean | 85.02% | 84.87% | 1.49% | 84.93% | | 2.93% | 93.16% |
| SVM-L-5-folds-std | 4.75% | 1.22% | 0.14% | 1.86% | | 0.27% | 0.84% |
| SVM-RBF-5-folds-std | 2.51% | 2.18% | 0.15% | 1.21% | | 0.30% | 1.30% |
| KNN(3k)-5-folds-std | 2.06% | 0.95% | 0.09% | 0.45% | | 0.17% | 1.13% |
| LDA-5-folds-std | 2.78% | 0.98% | 0.15% | 1.14% | | 0.30% | 0.64% |
| NBC-5-folds-std | 3.18% | 0.72% | 0.11% | 1.18% | | 0.21% | 1.02% |
| RF-5-folds-std | 3.19% | 1.06% | 0.27% | 1.54% | | 0.52% | 0.47% |

|  | **BenchSet – SMOTE(400%)** | | |  |  |  |  |
| --- | --- | --- | --- | --- | --- | --- | --- |
| Classifier | Sensitivity | Specificity | Precision | | G-Mean | F1-Measure | ROC-AUC |
| SVM-L-5-folds-mean | 64.30% | 96.42% | 4.60% | | 78.72% | 8.57% | 93.63% |
| SVM-RBF-5-folds-mean | 57.63% | 98.39% | 8.73% | | 75.25% | 15.13% | 94.43% |
| KNN(3k)-5-folds-mean | 74.44% | 86.88% | 1.49% | | 80.39% | 2.91% | 85.24% |
| LDA-5-folds-mean | 67.08% | 95.79% | 4.06% | | 80.12% | 7.65% | 93.73% |
| NBC-5-folds-mean | 78.03% | 77.48% | 0.92% | | 77.74% | 1.82% | 83.95% |
| RF-5-folds-mean | 35.26% | 99.88% | 42.84% | | 59.24% | 38.55% | 93.77% |
| SVM-L-5-folds-std | 2.96% | 0.29% | 0.68% | | 1.81% | 1.17% | 0.68% |
| SVM-RBF-5-folds-std | 4.59% | 0.13% | 0.93% | | 2.99% | 1.42% | 0.80% |
| KNN(3k)-5-folds-std | 4.33% | 0.39% | 0.11% | | 2.21% | 0.20% | 2.00% |
| LDA-5-folds-std | 5.05% | 0.09% | 0.29% | | 2.94% | 0.49% | 0.57% |
| NBC-5-folds-std | 3.91% | 0.84% | 0.16% | | 2.05% | 0.31% | 1.94% |
| RF-5-folds-std | 4.42% | 0.01% | 3.18% | | 3.85% | 3.38% | 1.00% |

|  | **BenchSet – MWMOTE** | | |  |  |  |  |
| --- | --- | --- | --- | --- | --- | --- | --- |
| Classifier | Sensitivity | Specificity | Precision | | G-Mean | F1-Measure | ROC-AUC |
| SVM-L-5-folds-mean | 65.46% | 93.95% | 2.82% | | 78.35% | 5.41% | 8.35% |
| SVM-RBF-5-folds-mean | 75.32% | 94.54% | 3.55% | | 84.36% | 6.77% | 5.15% |
| KNN(3k)-5-folds-mean | 71.41% | 87.47% | 1.49% | | 79.02% | 2.93% | 83.78% |
| LDA-5-folds-mean | 80.38% | 90.83% | 2.29% | | 85.44% | 4.45% | 93.97% |
| NBC-5-folds-mean | 79.34% | 74.67% | 0.83% | | 76.97% | 1.64% | 82.87% |
| RF-5-folds-mean | 45.02% | 99.46% | 18.43% | | 66.86% | 26.04% | 94.18% |
| SVM-L-5-folds-std | 6.24% | 0.55% | 0.50% | | 3.54% | 0.91% | 1.25% |
| SVM-RBF-5-folds-std | 3.98% | 0.31% | 0.34% | | 2.17% | 0.62% | 0.68% |
| KNN(3k)-5-folds-std | 2.57% | 0.09% | 0.13% | | 1.42% | 0.25% | 1.34% |
| LDA-5-folds-std | 2.10% | 0.61% | 0.30% | | 0.99% | 0.56% | 0.32% |
| NBC-5-folds-std | 2.05% | 1.04% | 0.11% | | 1.38% | 0.22% | 1.43% |
| RF-5-folds-std | 3.94% | 0.07% | 3.94% | | 2.99% | 4.42% | 0.31% |

|  | **BenchSet – GSVM-RU** | |  |  |  |  |  |
| --- | --- | --- | --- | --- | --- | --- | --- |
| Classifier | Sensitivity | Specificity | | Precision | G-Mean | F1-Measure | ROC-AUC |
| SVM-L-5-folds-mean | 62.02% | 97.32% | | 5.97% | 77.67% | 10.86% | 94.05% |
| SVM-RBF-5-folds-mean | 63.99% | 89.85% | | 1.70% | 75.69% | 3.30% | 86.46% |
| KNN(3k)-5-folds-mean | 71.95% | 72.53% | | 0.70% | 72.14% | 1.38% | 79.80% |
| LDA-5-folds-mean | 67.42% | 95.61% | | 3.97% | 80.24% | 7.49% | 93.18% |
| NBC-5-folds-mean | 78.64% | 76.10% | | 0.87% | 77.36% | 1.72% | 85.85% |
| RF-5-folds-mean | 67.18% | 94.48% | | 3.17% | 79.60% | 6.04% | 89.10% |
| SVM-L-5-folds-std | 3.24% | 0.55% | | 1.21% | 1.87% | 2.00% | 0.41% |
| SVM-RBF-5-folds-std | 7.69% | 2.10% | | 0.39% | 3.96% | 0.73% | 2.75% |
| KNN(3k)-5-folds-std | 6.73% | 2.64% | | 0.09% | 2.95% | 0.17% | 2.75% |
| LDA-5-folds-std | 5.00% | 0.59% | | 0.58% | 2.78% | 1.02% | 0.61% |
| NBC-5-folds-std | 2.15% | 0.72% | | 0.08% | 1.29% | 0.17% | 0.79% |
| RF-5-folds-std | 5.95% | 0.79% | | 0.41% | 3.38% | 0.75% | 2.32% |

|  | **BenchSet – DRAMOTE** | | |  |  |  |  |
| --- | --- | --- | --- | --- | --- | --- | --- |
| Classifier | Sensitivity | Specificity | Precision | | G-Mean | F1-Measure | ROC-AUC |
| SVM-L-5-folds-mean | 57.40% | 96.70% | 4.43% | | 74.47% | 8.22% | 92.76% |
| SVM-RBF-5-folds-mean | 56.61% | 98.67% | 10.23% | | 74.71% | 17.28% | 94.38% |
| KNN(3k)-5-folds-mean | 78.16% | 83.24% | 1.22% | | 80.62% | 2.41% | 85.39% |
| LDA-5-folds-mean | 68.65% | 95.55% | 3.95% | | 80.97% | 7.46% | 93.41% |
| NBC-5-folds-mean | 65.61% | 83.89% | 1.15% | | 73.87% | 2.26% | 82.12% |
| RF-5-folds-mean | 22.44% | 99.96% | 59.10% | | 47.16% | 32.07% | 92.36% |
| SVM-L-5-folds-std | 3.78% | 0.18% | 0.52% | | 2.42% | 0.90% | 1.19% |
| SVM-RBF-5-folds-std | 3.21% | 0.15% | 1.28% | | 2.15% | 1.83% | 0.76% |
| KNN(3k)-5-folds-std | 4.77% | 0.47% | 0.09% | | 2.34% | 0.17% | 2.31% |
| LDA-5-folds-std | 3.57% | 0.25% | 0.40% | | 2.12% | 0.70% | 0.82% |
| NBC-5-folds-std | 9.68% | 5.17% | 0.32% | | 4.07% | 0.61% | 3.06% |
| RF-5-folds-std | 4.49% | 0.02% | 9.72% | | 4.92% | 4.83% | 1.97% |

|  | **AID 596 – No preprocessing** | |  |  |  |  |  |
| --- | --- | --- | --- | --- | --- | --- | --- |
| Classifier | Sensitivity | Specificity | | Precision | G-Mean | F1-Measure | ROC-AUC |
| SVM-L-5-folds-mean | 0.08% | 100.00% | | 0.00% | 1.24% | 0.00% | 19.49% |
| SVM-RBF-5-folds-mean | 0.22% | 100.00% | | 0.00% | 3.67% | 0.00% | 17.80% |
| KNN(3k)-5-folds-mean | 13.37% | 99.61% | | 41.33% | 36.43% | 20.14% | 65.53% |
| LDA-5-folds-mean | 26.86% | 98.20% | | 23.56% | 51.32% | 25.03% | 86.11% |
| NBC-5-folds-mean | 52.46% | 85.46% | | 6.85% | 66.86% | 12.10% | 76.80% |
| RF-5-folds-mean | 9.93% | 99.94% | | 77.32% | 31.31% | 17.53% | 83.85% |
| SVM-L-5-folds-std | 0.17% | 0.01% | | 0.00% | 2.76% | 0.00% | 2.03% |
| SVM-RBF-5-folds-std | 0.21% | 0.00% | | 0.00% | 3.35% | 0.00% | 1.70% |
| KNN(3k)-5-folds-std | 1.76% | 0.07% | | 2.42% | 2.37% | 1.93% | 1.12% |
| LDA-5-folds-std | 2.53% | 0.28% | | 3.45% | 2.43% | 2.86% | 1.05% |
| NBC-5-folds-std | 5.21% | 1.75% | | 0.53% | 2.79% | 0.79% | 1.48% |
| RF-5-folds-std | 2.54% | 0.04% | | 13.85% | 3.88% | 4.18% | 1.53% |

|  | **AID 596 – Random Undersampling** | | | |  |  |  |
| --- | --- | --- | --- | --- | --- | --- | --- |
| Classifier | Sensitivity | Specificity | Precision | G-Mean | | F1-Measure | ROC-AUC |
| SVM-L-5-folds-mean | 77.07% | 72.87% | 5.47% | 74.94% | | 10.21% | 81.67% |
| SVM-RBF-5-folds-mean | 74.99% | 73.85% | 5.51% | 74.41% | | 10.27% | 82.20% |
| KNN(3k)-5-folds-mean | 70.37% | 61.27% | 3.57% | 65.65% | | 6.79% | 71.89% |
| LDA-5-folds-mean | 78.69% | 76.21% | 6.32% | 77.43% | | 11.69% | 85.50% |
| NBC-5-folds-mean | 76.01% | 65.51% | 4.32% | 70.52% | | 8.18% | 75.34% |
| RF-5-folds-mean | 78.25% | 77.58% | 6.62% | 77.91% | | 12.21% | 85.71% |
| SVM-L-5-folds-std | 1.47% | 0.78% | 0.42% | 0.77% | | 0.73% | 1.08% |
| SVM-RBF-5-folds-std | 2.50% | 1.51% | 0.35% | 1.19% | | 0.60% | 1.07% |
| KNN(3k)-5-folds-std | 1.94% | 1.70% | 0.30% | 1.04% | | 0.53% | 1.24% |
| LDA-5-folds-std | 2.98% | 1.00% | 0.64% | 1.57% | | 1.12% | 1.25% |
| NBC-5-folds-std | 2.15% | 4.05% | 0.50% | 2.01% | | 0.89% | 1.59% |
| RF-5-folds-std | 1.42% | 2.68% | 0.33% | 1.33% | | 0.59% | 1.06% |

|  | **AID 596 – SMOTE(200%)** | | |  |  |  |  |
| --- | --- | --- | --- | --- | --- | --- | --- |
| Classifier | Sensitivity | Specificity | Precision | | G-Mean | F1-Measure | ROC-AUC |
| SVM-L-5-folds-mean | 69.20% | 86.31% | 9.33% | | 77.27% | 16.43% | 85.46% |
| SVM-RBF-5-folds-mean | 59.36% | 90.37% | 11.15% | | 73.19% | 18.76% | 84.63% |
| KNN(3k)-5-folds-mean | 73.45% | 62.31% | 3.81% | | 67.64% | 7.24% | 73.45% |
| LDA-5-folds-mean | 69.50% | 86.49% | 9.50% | | 77.52% | 16.70% | 86.55% |
| NBC-5-folds-mean | 74.56% | 59.53% | 3.62% | | 66.60% | 6.90% | 73.96% |
| RF-5-folds-mean | 38.06% | 98.01% | 28.00% | | 61.04% | 32.25% | 86.84% |
| SVM-L-5-folds-std | 2.62% | 0.44% | 0.76% | | 1.43% | 1.21% | 1.29% |
| SVM-RBF-5-folds-std | 4.51% | 0.82% | 0.87% | | 2.54% | 1.25% | 1.39% |
| KNN(3k)-5-folds-std | 1.97% | 0.74% | 0.25% | | 0.72% | 0.44% | 1.04% |
| LDA-5-folds-std | 3.01% | 0.83% | 1.01% | | 1.74% | 1.61% | 1.19% |
| NBC-5-folds-std | 2.77% | 2.43% | 0.35% | | 1.44% | 0.64% | 1.68% |
| RF-5-folds-std | 1.83% | 1.61% | 0.23% | | 0.96% | 0.42% | 1.11% |

|  | **AID 596 – MWMOTE** | |  |  |  |  |  |
| --- | --- | --- | --- | --- | --- | --- | --- |
| Classifier | Sensitivity | Specificity | | Precision | G-Mean | F1-Measure | ROC-AUC |
| SVM-L-5-folds-mean | 67.56% | 85.07% | | 8.42% | 75.78% | 14.96% | 16.08% |
| SVM-RBF-5-folds-mean | 58.32% | 90.14% | | 10.73% | 72.48% | 18.11% | 15.61% |
| KNN(3k)-5-folds-mean | 58.67% | 76.63% | | 4.86% | 67.05% | 8.98% | 71.97% |
| LDA-5-folds-mean | 69.16% | 85.91% | | 9.09% | 77.07% | 16.06% | 85.93% |
| NBC-5-folds-mean | 80.70% | 47.50% | | 3.04% | 61.86% | 5.86% | 73.18% |
| RF-5-folds-mean | 38.21% | 98.06% | | 28.66% | 61.19% | 32.70% | 86.86% |
| SVM-L-5-folds-std | 3.99% | 0.48% | | 0.57% | 2.09% | 0.93% | 0.98% |
| SVM-RBF-5-folds-std | 3.22% | 0.63% | | 0.57% | 1.76% | 0.72% | 1.26% |
| KNN(3k)-5-folds-std | 1.22% | 0.74% | | 0.40% | 0.76% | 0.69% | 1.03% |
| LDA-5-folds-std | 2.88% | 0.69% | | 0.83% | 1.56% | 1.32% | 1.27% |
| NBC-5-folds-std | 3.64% | 3.39% | | 0.32% | 2.41% | 0.60% | 2.44% |
| RF-5-folds-std | 2.41% | 2.24% | | 0.21% | 1.60% | 0.40% | 1.62% |

|  | **AID 596 – GSVM-RU** | |  |  |  |  |  |
| --- | --- | --- | --- | --- | --- | --- | --- |
| Classifier | Sensitivity | Specificity | | Precision | G-Mean | F1-Measure | ROC-AUC |
| SVM-L-5-folds-mean | 72.79% | 85.57% | | 9.33% | 78.89% | 16.53% | 86.32% |
| SVM-RBF-5-folds-mean | 95.20% | 19.26% | | 2.35% | 42.24% | 4.58% | 78.78% |
| KNN(3k)-5-folds-mean | 80.55% | 39.07% | | 2.62% | 56.07% | 5.07% | 66.39% |
| LDA-5-folds-mean | 78.04% | 77.15% | | 6.50% | 77.54% | 12.00% | 85.35% |
| NBC-5-folds-mean | 81.71% | 45.39% | | 3.01% | 60.58% | 5.80% | 73.15% |
| RF-5-folds-mean | 88.39% | 50.97% | | 3.54% | 67.09% | 6.80% | 82.97% |
| SVM-L-5-folds-std | 3.31% | 1.51% | | 0.60% | 1.22% | 0.91% | 0.59% |
| SVM-RBF-5-folds-std | 2.80% | 6.08% | | 0.20% | 6.88% | 0.38% | 2.64% |
| KNN(3k)-5-folds-std | 2.75% | 1.84% | | 0.14% | 0.94% | 0.26% | 1.46% |
| LDA-5-folds-std | 3.93% | 2.10% | | 0.33% | 0.91% | 0.55% | 0.80% |
| NBC-5-folds-std | 3.72% | 8.89% | | 0.50% | 4.63% | 0.92% | 1.86% |
| RF-5-folds-std | 2.47% | 2.16% | | 0.22% | 1.09% | 0.42% | 1.23% |

|  | **AID 596 – DRAMOTE** | |  |  |  |  |  |
| --- | --- | --- | --- | --- | --- | --- | --- |
| Classifier | Sensitivity | Specificity | | Precision | G-Mean | F1-Measure | ROC-AUC |
| SVM-L-5-folds-mean | 54.32% | 92.17% | | 12.45% | 70.74% | 20.22% | 84.14% |
| SVM-RBF-5-folds-mean | 29.28% | 97.94% | | 22.41% | 53.44% | 25.35% | 82.46% |
| KNN(3k)-5-folds-mean | 45.07% | 85.87% | | 6.13% | 62.11% | 10.77% | 71.33% |
| LDA-5-folds-mean | 48.65% | 94.01% | | 14.22% | 67.61% | 21.96% | 84.94% |
| NBC-5-folds-mean | 56.53% | 64.56% | | 3.15% | 60.35% | 5.97% | 64.00% |
| RF-5-folds-mean | 23.55% | 99.56% | | 52.07% | 48.29% | 32.30% | 86.28% |
| SVM-L-5-folds-std | 1.96% | 0.82% | | 1.61% | 1.23% | 2.16% | 1.17% |
| SVM-RBF-5-folds-std | 4.04% | 0.20% | | 2.37% | 3.71% | 2.91% | 2.24% |
| KNN(3k)-5-folds-std | 5.25% | 1.73% | | 0.87% | 3.40% | 1.37% | 2.26% |
| LDA-5-folds-std | 2.18% | 0.60% | | 1.18% | 1.33% | 1.34% | 0.86% |
| NBC-5-folds-std | 3.63% | 3.32% | | 0.27% | 1.84% | 0.49% | 1.79% |
| RF-5-folds-std | 2.40% | 2.20% | | 0.18% | 1.22% | 0.32% | 1.19% |

|  | **AID 618 – No preprocessing** | |  |  |  |  |
| --- | --- | --- | --- | --- | --- | --- |
| Classifier | Sensitivity | Specificity | | Precision | G-Mean | F1-Measure |
| SVM-L-5-folds-mean | 0 | 1 | | 0 | 0 | 0 |
| SVM-RBF-5-folds-mean | 0 | 1 | | 0 | 0 | 0 |
| KNN(3k)-5-folds-mean | 0.113147 | 0.998863 | | 0.369158 | 0.330797 | 0.171913 |
| LDA-5-folds-mean | 0.16642 | 0.994395 | | 0.154043 | 0.40273 | 0.159841 |
| NBC-5-folds-mean | 0.72473 | 0.631679 | | 0.01205 | 0.675627 | 0.023696 |
| RF-5-folds-mean | 0.047057 | 0.999884 | | 0.735714 | 0.212736 | 0.087643 |
| SVM-L-5-folds-std | 0 | 0 | | 0 | 0 | 0 |
| SVM-RBF-5-folds-std | 0 | 0 | | 0 | 0 | 0 |
| KNN(3k)-5-folds-std | 0.045811 | 0.000066 | | 0.078008 | 0.067003 | 0.060063 |
| LDA-5-folds-std | 0.052408 | 0.000732 | | 0.039846 | 0.064282 | 0.045655 |
| NBC-5-folds-std | 0.066392 | 0.018096 | | 0.001448 | 0.022005 | 0.002784 |
| RF-5-folds-std | 0.021578883 | 9.17E-05 | | 0.211355629 | 0.047363754 | 0.037909695 |

|  | **AID 618 – Random Undersampling** | | | |  |  |  |
| --- | --- | --- | --- | --- | --- | --- | --- |
| Classifier | Sensitivity | Specificity | Precision | G-Mean | | F1-Measure | ROC-AUC |
| SVM-L-5-folds-mean | 72.77% | 65.87% | 1.31% | 69.22% | | 2.57% | 74.62% |
| SVM-RBF-5-folds-mean | 68.36% | 70.45% | 1.42% | 69.39% | | 2.79% | 77.16% |
| KNN(3k)-5-folds-mean | 70.75% | 62.08% | 1.15% | 66.23% | | 2.26% | 72.46% |
| LDA-5-folds-mean | 75.87% | 70.17% | 1.55% | 72.93% | | 3.04% | 78.80% |
| NBC-5-folds-mean | 77.17% | 51.79% | 0.98% | 63.13% | | 1.94% | 72.01% |
| RF-5-folds-mean | 70.34% | 76.70% | 1.85% | 73.43% | | 3.61% | 81.06% |
| SVM-L-5-folds-std | 2.51% | 1.50% | 0.15% | 1.24% | | 0.30% | 1.72% |
| SVM-RBF-5-folds-std | 0.46% | 2.93% | 0.19% | 1.66% | | 0.37% | 1.84% |
| KNN(3k)-5-folds-std | 3.72% | 2.15% | 0.18% | 1.18% | | 0.34% | 2.00% |
| LDA-5-folds-std | 5.02% | 1.13% | 0.19% | 2.43% | | 0.36% | 1.46% |
| NBC-5-folds-std | 5.70% | 2.43% | 0.11% | 1.32% | | 0.22% | 3.43% |
| RF-5-folds-std | 7.41% | 1.68% | 0.67% | 3.22% | | 1.15% | 4.24% |

|  | **AID 618 – SMOTE(400%)** | | |  |  |  |  |
| --- | --- | --- | --- | --- | --- | --- | --- |
| Classifier | Sensitivity | Specificity | Precision | | G-Mean | F1-Measure | ROC-AUC |
| SVM-L-5-folds-mean | 41.41% | 94.72% | 4.65% | | 62.56% | 8.33% | 80.54% |
| SVM-RBF-5-folds-mean | 31.21% | 98.84% | 14.20% | | 55.17% | 19.23% | 81.83% |
| KNN(3k)-5-folds-mean | 56.80% | 84.36% | 2.21% | | 69.11% | 4.25% | 74.90% |
| LDA-5-folds-mean | 42.45% | 94.78% | 4.83% | | 63.36% | 8.65% | 81.01% |
| NBC-5-folds-mean | 68.25% | 61.38% | 1.08% | | 64.59% | 2.12% | 69.82% |
| RF-5-folds-mean | 17.96% | 99.79% | 33.46% | | 41.63% | 23.01% | 82.86% |
| SVM-L-5-folds-std | 4.23% | 0.47% | 0.61% | | 3.12% | 0.94% | 1.57% |
| SVM-RBF-5-folds-std | 7.78% | 0.22% | 2.42% | | 7.12% | 2.90% | 1.25% |
| KNN(3k)-5-folds-std | 6.61% | 0.95% | 0.40% | | 3.92% | 0.74% | 3.43% |
| LDA-5-folds-std | 4.35% | 0.46% | 0.82% | | 3.16% | 1.28% | 2.37% |
| NBC-5-folds-std | 7.68% | 2.12% | 0.10% | | 2.53% | 0.19% | 2.63% |
| RF-5-folds-std | 9.99% | 1.47% | 0.59% | | 6.17% | 1.00% | 3.25% |

|  | **AID 618 – MWMOTE** | |  |  |  |  |  |
| --- | --- | --- | --- | --- | --- | --- | --- |
| Classifier | Sensitivity | Specificity | | Precision | G-Mean | F1-Measure | ROC-AUC |
| SVM-L-5-folds-mean | 42.51% | 93.96% | | 4.19% | 63.12% | 7.62% | 19.87% |
| SVM-RBF-5-folds-mean | 32.68% | 98.63% | | 12.73% | 56.34% | 18.04% | 17.11% |
| KNN(3k)-5-folds-mean | 44.47% | 93.75% | | 4.31% | 64.30% | 7.83% | 72.21% |
| LDA-5-folds-mean | 41.15% | 94.63% | | 4.59% | 62.35% | 8.23% | 81.43% |
| NBC-5-folds-mean | 74.72% | 55.06% | | 1.02% | 64.03% | 2.00% | 70.70% |
| RF-5-folds-mean | 18.48% | 99.79% | | 35.03% | 42.52% | 24.05% | 83.83% |
| SVM-L-5-folds-std | 4.46% | 0.43% | | 0.61% | 3.32% | 1.02% | 1.62% |
| SVM-RBF-5-folds-std | 8.87% | 0.27% | | 1.43% | 7.71% | 2.00% | 2.22% |
| KNN(3k)-5-folds-std | 8.50% | 0.62% | | 1.39% | 6.64% | 2.37% | 4.49% |
| LDA-5-folds-std | 3.80% | 0.47% | | 0.93% | 2.84% | 1.51% | 1.97% |
| NBC-5-folds-std | 8.11% | 1.61% | | 0.10% | 2.74% | 0.19% | 2.80% |
| RF-5-folds-std | 10.55% | 1.11% | | 0.59% | 6.67% | 1.02% | 3.46% |

|  | **AID 618 – GSVM-RU** | |  |  |  |  |  |
| --- | --- | --- | --- | --- | --- | --- | --- |
| Classifier | Sensitivity | Specificity | | Precision | G-Mean | F1-Measure | ROC-AUC |
| SVM-L-5-folds-mean | 43.24% | 94.57% | | 4.85% | 63.61% | 8.63% | 82.17% |
| SVM-RBF-5-folds-mean | 40.85% | 89.79% | | 2.57% | 60.16% | 4.79% | 74.40% |
| KNN(3k)-5-folds-mean | 58.51% | 67.43% | | 1.11% | 62.71% | 2.17% | 68.66% |
| LDA-5-folds-mean | 47.65% | 89.64% | | 2.83% | 65.21% | 5.33% | 78.01% |
| NBC-5-folds-mean | 72.73% | 52.92% | | 0.95% | 61.94% | 1.87% | 70.47% |
| RF-5-folds-mean | 51.56% | 91.07% | | 3.52% | 68.32% | 6.56% | 77.16% |
| SVM-L-5-folds-std | 9.65% | 1.82% | | 0.87% | 6.16% | 1.32% | 1.35% |
| SVM-RBF-5-folds-std | 9.16% | 4.04% | | 0.63% | 5.36% | 1.09% | 1.86% |
| KNN(3k)-5-folds-std | 6.28% | 2.59% | | 0.16% | 3.29% | 0.32% | 4.26% |
| LDA-5-folds-std | 6.20% | 2.23% | | 0.52% | 3.24% | 0.90% | 2.64% |
| NBC-5-folds-std | 6.29% | 2.78% | | 0.11% | 1.96% | 0.21% | 2.62% |
| RF-5-folds-std | 8.18% | 1.92% | | 0.63% | 4.77% | 1.08% | 3.24% |

|  | **AID 618 – DRAMOTE** | |  |  |  |  |  |
| --- | --- | --- | --- | --- | --- | --- | --- |
| Classifier | Sensitivity | Specificity | | Precision | G-Mean | F1-Measure | ROC-AUC |
| SVM-L-5-folds-mean | 30.48% | 94.98% | | 3.58% | 53.51% | 6.39% | 76.19% |
| SVM-RBF-5-folds-mean | 11.35% | 99.76% | | 22.61% | 33.31% | 14.92% | 78.86% |
| KNN(3k)-5-folds-mean | 33.67% | 95.12% | | 4.12% | 56.26% | 7.30% | 72.63% |
| LDA-5-folds-mean | 40.23% | 94.99% | | 4.74% | 61.68% | 8.45% | 80.38% |
| NBC-5-folds-mean | 49.85% | 74.19% | | 1.22% | 60.42% | 2.37% | 65.36% |
| RF-5-folds-mean | 12.55% | 99.89% | | 40.41% | 35.00% | 18.97% | 82.24% |
| SVM-L-5-folds-std | 6.95% | 0.52% | | 0.52% | 6.20% | 0.94% | 1.50% |
| SVM-RBF-5-folds-std | 3.29% | 0.05% | | 7.03% | 5.29% | 3.93% | 2.20% |
| KNN(3k)-5-folds-std | 7.81% | 0.65% | | 1.09% | 6.68% | 1.77% | 3.55% |
| LDA-5-folds-std | 5.75% | 0.52% | | 0.65% | 4.34% | 1.00% | 2.64% |
| NBC-5-folds-std | 8.64% | 6.13% | | 0.27% | 3.02% | 0.51% | 1.71% |
| RF-5-folds-std | 4.10% | 0.02% | | 6.48% | 5.93% | 5.36% | 1.83% |

|  | **AID 644 – No preprocessing** | |  |  |  |  |  |
| --- | --- | --- | --- | --- | --- | --- | --- |
| Classifier | Sensitivity | Specificity | | Precision | G-Mean | F1-Measure | ROC-AUC |
| SVM-L-5-folds-mean | 38.48% | 70.45% | | 38.20% | 51.17% | 37.81% | 57.10% |
| SVM-RBF-5-folds-mean | 0.00% | 100.00% | | 0.00% | 0.00% | 0.00% | 62.53% |
| KNN(3k)-5-folds-mean | 37.20% | 74.45% | | 41.00% | 52.15% | 37.87% | 54.83% |
| LDA-5-folds-mean | 35.55% | 75.39% | | 39.45% | 50.90% | 36.87% | 58.19% |
| NBC-5-folds-mean | 39.30% | 78.07% | | 44.12% | 52.94% | 38.85% | 59.38% |
| RF-5-folds-mean | 20.24% | 92.79% | | 54.95% | 41.08% | 28.59% | 60.18% |
| SVM-L-5-folds-std | 14.27% | 7.94% | | 12.88% | 10.45% | 12.64% | 9.67% |
| SVM-RBF-5-folds-std | 0.00% | 0.00% | | 0.00% | 0.00% | 0.00% | 7.09% |
| KNN(3k)-5-folds-std | 9.56% | 5.91% | | 9.90% | 6.53% | 6.39% | 7.87% |
| LDA-5-folds-std | 16.76% | 3.41% | | 11.07% | 12.38% | 13.43% | 11.27% |
| NBC-5-folds-std | 23.06% | 8.26% | | 9.47% | 16.40% | 16.60% | 12.21% |
| RF-5-folds-std | 13.65% | 4.35% | | 21.17% | 15.82% | 17.32% | 11.25% |

|  | **AID 644 – Random Undersampling** | | | |  |  |  |
| --- | --- | --- | --- | --- | --- | --- | --- |
| Classifier | Sensitivity | Specificity | Precision | G-Mean | | F1-Measure | ROC-AUC |
| SVM-L-5-folds-mean | 54.65% | 54.26% | 37.01% | 54.12% | | 43.70% | 54.80% |
| SVM-RBF-5-folds-mean | 53.01% | 47.97% | 33.26% | 48.73% | | 39.56% | 54.76% |
| KNN(3k)-5-folds-mean | 49.20% | 49.21% | 32.17% | 49.08% | | 38.33% | 50.11% |
| LDA-5-folds-mean | 51.58% | 50.88% | 34.24% | 50.78% | | 40.54% | 53.95% |
| NBC-5-folds-mean | 41.98% | 66.04% | 34.68% | 50.11% | | 35.90% | 56.64% |
| RF-5-folds-mean | 51.29% | 61.16% | 39.13% | 55.75% | | 43.89% | 59.89% |
| SVM-L-5-folds-std | 10.85% | 14.19% | 8.85% | 10.66% | | 8.09% | 11.74% |
| SVM-RBF-5-folds-std | 13.71% | 15.68% | 9.03% | 4.19% | | 6.06% | 7.03% |
| KNN(3k)-5-folds-std | 4.47% | 7.76% | 9.81% | 5.02% | | 7.60% | 7.32% |
| LDA-5-folds-std | 13.09% | 16.04% | 11.73% | 12.75% | | 10.38% | 14.81% |
| NBC-5-folds-std | 21.42% | 9.72% | 5.65% | 13.17% | | 12.65% | 10.26% |
| RF-5-folds-std | 4.68% | 8.77% | 3.37% | 3.14% | | 3.31% | 4.20% |

|  | **AID 644 – SMOTE(200%)** | | |  |  |  |  |
| --- | --- | --- | --- | --- | --- | --- | --- |
| Classifier | Sensitivity | Specificity | Precision | | G-Mean | F1-Measure | ROC-AUC |
| SVM-L-5-folds-mean | 38.85% | 69.80% | 37.85% | | 50.65% | 37.35% | 57.77% |
| SVM-RBF-5-folds-mean | 66.23% | 39.02% | 34.48% | | 47.38% | 42.83% | 58.53% |
| KNN(3k)-5-folds-mean | 61.88% | 45.37% | 35.74% | | 52.43% | 44.25% | 56.99% |
| LDA-5-folds-mean | 43.87% | 72.53% | 42.82% | | 56.11% | 42.99% | 58.45% |
| NBC-5-folds-mean | 43.80% | 77.65% | 46.50% | | 55.52% | 41.75% | 61.70% |
| RF-5-folds-mean | 29.20% | 87.41% | 53.31% | | 49.64% | 36.54% | 64.41% |
| SVM-L-5-folds-std | 15.92% | 10.45% | 11.78% | | 10.53% | 12.53% | 8.68% |
| SVM-RBF-5-folds-std | 21.22% | 20.20% | 8.94% | | 6.64% | 5.73% | 9.09% |
| KNN(3k)-5-folds-std | 9.79% | 11.23% | 11.16% | | 6.49% | 8.87% | 3.68% |
| LDA-5-folds-std | 12.09% | 3.35% | 9.87% | | 8.73% | 9.76% | 10.23% |
| NBC-5-folds-std | 23.95% | 9.27% | 10.65% | | 16.12% | 16.50% | 10.02% |
| RF-5-folds-std | 5.23% | 8.37% | 6.35% | | 3.84% | 4.32% | 4.10% |

|  | **AID 644 – MWMOTE** | |  |  |  |  |  |
| --- | --- | --- | --- | --- | --- | --- | --- |
| Classifier | Sensitivity | Specificity | | Precision | G-Mean | F1-Measure | ROC-AUC |
| SVM-L-5-folds-mean | 38.48% | 70.45% | | 38.20% | 51.17% | 37.81% | 42.90% |
| SVM-RBF-5-folds-mean | 67.83% | 40.23% | | 35.21% | 51.29% | 45.16% | 39.93% |
| KNN(3k)-5-folds-mean | 55.10% | 54.29% | | 36.73% | 53.79% | 42.86% | 54.66% |
| LDA-5-folds-mean | 42.97% | 73.56% | | 42.75% | 55.26% | 41.78% | 59.12% |
| NBC-5-folds-mean | 46.08% | 77.50% | | 50.25% | 58.59% | 45.62% | 64.24% |
| RF-5-folds-mean | 33.73% | 83.94% | | 50.17% | 52.37% | 38.71% | 63.37% |
| SVM-L-5-folds-std | 14.27% | 7.94% | | 12.88% | 10.45% | 12.64% | 9.67% |
| SVM-RBF-5-folds-std | 14.28% | 11.44% | | 8.43% | 7.22% | 6.58% | 8.87% |
| KNN(3k)-5-folds-std | 14.98% | 11.30% | | 9.06% | 6.41% | 7.19% | 7.09% |
| LDA-5-folds-std | 18.37% | 4.99% | | 13.40% | 11.59% | 12.74% | 12.25% |
| NBC-5-folds-std | 19.30% | 10.29% | | 16.72% | 12.57% | 13.11% | 11.92% |
| RF-5-folds-std | 3.93% | 5.29% | | 6.28% | 2.54% | 3.50% | 2.40% |

|  | **AID 644 – GSVM-RU** | |  |  |  |  |  |
| --- | --- | --- | --- | --- | --- | --- | --- |
| Classifier | Sensitivity | Specificity | | Precision | G-Mean | F1-Measure | ROC-AUC |
| SVM-L-5-folds-mean | 60.54% | 46.18% | | 35.13% | 52.64% | 44.02% | 56.55% |
| SVM-RBF-5-folds-mean | 90.25% | 14.25% | | 33.82% | 34.07% | 48.51% | 58.24% |
| KNN(3k)-5-folds-mean | 71.08% | 30.68% | | 33.04% | 45.64% | 44.26% | 48.88% |
| LDA-5-folds-mean | 67.25% | 43.44% | | 36.39% | 53.88% | 46.86% | 57.94% |
| NBC-5-folds-mean | 56.74% | 56.96% | | 38.21% | 55.22% | 44.03% | 59.78% |
| RF-5-folds-mean | 81.83% | 38.91% | | 39.50% | 55.41% | 52.05% | 64.76% |
| SVM-L-5-folds-std | 13.03% | 5.96% | | 10.94% | 7.32% | 11.18% | 9.60% |
| SVM-RBF-5-folds-std | 5.89% | 10.12% | | 8.86% | 11.36% | 8.84% | 8.36% |
| KNN(3k)-5-folds-std | 13.28% | 10.16% | | 8.07% | 5.68% | 7.07% | 5.60% |
| LDA-5-folds-std | 14.28% | 6.31% | | 13.05% | 8.85% | 13.91% | 13.31% |
| NBC-5-folds-std | 22.29% | 11.62% | | 15.74% | 11.49% | 14.23% | 10.50% |
| RF-5-folds-std | 12.72% | 13.31% | | 11.08% | 6.67% | 8.26% | 9.27% |

|  | **AID 644 – DRAMOTE** | |  |  |  |  |  |
| --- | --- | --- | --- | --- | --- | --- | --- |
| Classifier | Sensitivity | Specificity | | Precision | G-Mean | F1-Measure | ROC-AUC |
| SVM-L-5-folds-mean | 38.48% | 70.45% | | 38.20% | 51.17% | 37.81% | 57.10% |
| SVM-RBF-5-folds-mean | 76.43% | 34.25% | | 35.98% | 50.89% | 48.28% | 60.07% |
| KNN(3k)-5-folds-mean | 63.16% | 37.06% | | 32.57% | 47.54% | 42.25% | 54.83% |
| LDA-5-folds-mean | 36.45% | 73.61% | | 40.00% | 51.49% | 37.40% | 57.94% |
| NBC-5-folds-mean | 38.44% | 80.80% | | 47.55% | 54.52% | 41.43% | 60.59% |
| RF-5-folds-mean | 35.27% | 86.10% | | 56.80% | 54.25% | 41.29% | 61.56% |
| SVM-L-5-folds-std | 14.27% | 7.94% | | 12.88% | 10.45% | 12.64% | 9.67% |
| SVM-RBF-5-folds-std | 6.85% | 7.54% | | 9.73% | 6.25% | 9.00% | 8.61% |
| KNN(3k)-5-folds-std | 13.62% | 10.90% | | 10.18% | 7.80% | 10.04% | 7.63% |
| LDA-5-folds-std | 10.63% | 5.70% | | 12.76% | 8.39% | 9.79% | 11.51% |
| NBC-5-folds-std | 17.88% | 4.91% | | 17.48% | 14.12% | 15.85% | 10.35% |
| RF-5-folds-std | 10.58% | 2.70% | | 15.32% | 10.01% | 12.57% | 15.18% |

|  | **AID 886 – No preprocessing** | |  |  |  |  |  |
| --- | --- | --- | --- | --- | --- | --- | --- |
| Classifier | Sensitivity | Specificity | | Precision | G-Mean | F1-Measure | ROC-AUC |
| SVM-L-5-folds-mean | 90.06% | 99.64% | | 90.41% | 94.72% | 90.22% | 99.86% |
| SVM-RBF-5-folds-mean | 92.54% | 99.47% | | 87.01% | 95.94% | 89.68% | 99.02% |
| KNN(3k)-5-folds-mean | 88.15% | 99.58% | | 88.80% | 93.69% | 88.47% | 97.90% |
| LDA-5-folds-mean | 99.55% | 98.48% | | 71.43% | 99.02% | 83.17% | 99.31% |
| NBC-5-folds-mean | 95.15% | 98.91% | | 76.87% | 97.01% | 85.02% | 97.87% |
| RF-5-folds-mean | 94.41% | 99.59% | | 89.84% | 96.96% | 92.06% | 99.82% |
| SVM-L-5-folds-std | 1.54% | 0.05% | | 1.07% | 0.80% | 0.68% | 0.02% |
| SVM-RBF-5-folds-std | 1.23% | 0.08% | | 1.68% | 0.64% | 1.15% | 0.21% |
| KNN(3k)-5-folds-std | 0.84% | 0.05% | | 1.33% | 0.45% | 0.93% | 0.43% |
| LDA-5-folds-std | 0.18% | 0.12% | | 1.64% | 0.10% | 1.11% | 0.10% |
| NBC-5-folds-std | 0.81% | 0.14% | | 2.19% | 0.41% | 1.44% | 0.49% |
| RF-5-folds-std | 1.46% | 0.77% | | 14.62% | 0.62% | 7.95% | 2.18% |

|  | **AID 886 – Random Undersampling** | | | |  |  |  |
| --- | --- | --- | --- | --- | --- | --- | --- |
| Classifier | Sensitivity | Specificity | Precision | G-Mean | | F1-Measure | ROC-AUC |
| SVM-L-5-folds-mean | 99.27% | 98.46% | 71.07% | 98.86% | | 82.82% | 99.69% |
| SVM-RBF-5-folds-mean | 99.43% | 98.12% | 66.79% | 98.77% | | 79.89% | 99.54% |
| KNN(3k)-5-folds-mean | 99.67% | 98.27% | 68.64% | 98.97% | | 81.28% | 99.33% |
| LDA-5-folds-mean | 99.92% | 97.82% | 63.53% | 98.86% | | 77.66% | 99.30% |
| NBC-5-folds-mean | 99.14% | 98.30% | 68.95% | 98.72% | | 81.33% | 99.09% |
| RF-5-folds-mean | 99.84% | 98.12% | 66.93% | 98.98% | | 80.13% | 99.72% |
| SVM-L-5-folds-std | 0.24% | 0.15% | 2.27% | 0.15% | | 1.56% | 0.05% |
| SVM-RBF-5-folds-std | 0.26% | 0.16% | 2.11% | 0.11% | | 1.46% | 0.08% |
| KNN(3k)-5-folds-std | 0.18% | 0.14% | 2.07% | 0.13% | | 1.46% | 0.09% |
| LDA-5-folds-std | 0.11% | 0.12% | 1.53% | 0.09% | | 1.15% | 0.09% |
| NBC-5-folds-std | 0.53% | 0.07% | 0.95% | 0.25% | | 0.64% | 0.09% |
| RF-5-folds-std | 0.05% | 0.93% | 3.58% | 0.58% | | 3.90% | 0.07% |

|  | **AID 886 – SMOTE(200%)** | | |  |  |  |  |
| --- | --- | --- | --- | --- | --- | --- | --- |
| Classifier | Sensitivity | Specificity | Precision | | G-Mean | F1-Measure | ROC-AUC |
| SVM-L-5-folds-mean | 96.17% | 99.10% | 80.30% | | 97.62% | 87.52% | 99.79% |
| SVM-RBF-5-folds-mean | 99.11% | 98.58% | 72.60% | | 98.84% | 83.80% | 99.72% |
| KNN(3k)-5-folds-mean | 98.86% | 98.65% | 73.51% | | 98.75% | 84.32% | 99.36% |
| LDA-5-folds-mean | 99.71% | 98.18% | 67.54% | | 98.94% | 80.52% | 99.42% |
| NBC-5-folds-mean | 88.94% | 99.09% | 79.89% | | 93.80% | 83.62% | 95.45% |
| RF-5-folds-mean | 98.86% | 98.90% | 77.39% | | 98.88% | 86.80% | 99.86% |
| SVM-L-5-folds-std | 0.49% | 0.06% | 0.98% | | 0.24% | 0.62% | 0.03% |
| SVM-RBF-5-folds-std | 0.36% | 0.06% | 1.00% | | 0.17% | 0.64% | 0.10% |
| KNN(3k)-5-folds-std | 0.18% | 0.06% | 1.05% | | 0.07% | 0.66% | 0.09% |
| LDA-5-folds-std | 0.35% | 0.11% | 1.41% | | 0.16% | 1.00% | 0.25% |
| NBC-5-folds-std | 7.79% | 0.51% | 7.62% | | 3.85% | 1.61% | 3.14% |
| RF-5-folds-std | 11.09% | 0.25% | 3.59% | | 5.68% | 3.84% | 4.94% |

|  | **AID 886 – MWMOTE** | |  |  |  |  |  |
| --- | --- | --- | --- | --- | --- | --- | --- |
| Classifier | Sensitivity | Specificity | | Precision | G-Mean | F1-Measure | ROC-AUC |
| SVM-L-5-folds-mean | 95.36% | 99.13% | | 80.75% | 97.23% | 87.44% | 0.22% |
| SVM-RBF-5-folds-mean | 98.78% | 98.53% | | 71.84% | 98.65% | 83.18% | 0.22% |
| KNN(3k)-5-folds-mean | 98.25% | 98.66% | | 73.66% | 98.45% | 84.19% | 99.24% |
| LDA-5-folds-mean | 99.75% | 98.10% | | 66.71% | 98.93% | 79.94% | 99.36% |
| NBC-5-folds-mean | 91.10% | 98.86% | | 75.97% | 94.88% | 82.57% | 96.46% |
| RF-5-folds-mean | 98.94% | 98.87% | | 77.00% | 98.91% | 86.59% | 99.86% |
| SVM-L-5-folds-std | 0.65% | 0.07% | | 1.38% | 0.36% | 1.05% | 0.02% |
| SVM-RBF-5-folds-std | 0.52% | 0.06% | | 1.03% | 0.26% | 0.76% | 0.04% |
| KNN(3k)-5-folds-std | 0.33% | 0.07% | | 1.33% | 0.17% | 0.92% | 0.16% |
| LDA-5-folds-std | 0.27% | 0.13% | | 1.66% | 0.14% | 1.20% | 0.27% |
| NBC-5-folds-std | 4.19% | 0.48% | | 6.70% | 1.97% | 2.69% | 1.73% |
| RF-5-folds-std | 8.77% | 0.11% | | 2.20% | 4.51% | 4.67% | 3.80% |

|  | **AID 886 – GSVM-RU** | |  |  |  |  |  |
| --- | --- | --- | --- | --- | --- | --- | --- |
| Classifier | Sensitivity | Specificity | | Precision | G-Mean | F1-Measure | ROC-AUC |
| SVM-L-5-folds-mean | 99.67% | 98.39% | | 70.37% | 99.03% | 82.44% | 99.66% |
| SVM-RBF-5-folds-mean | 99.71% | 1.80% | | 3.72% | 12.70% | 7.17% | 98.84% |
| KNN(3k)-5-folds-mean | 99.63% | 97.40% | | 60.73% | 98.51% | 75.06% | 99.03% |
| LDA-5-folds-mean | 99.31% | 98.28% | | 68.79% | 98.79% | 81.25% | 99.33% |
| NBC-5-folds-mean | 97.30% | 98.74% | | 74.72% | 98.02% | 84.49% | 99.24% |
| RF-5-folds-mean | 99.88% | 95.47% | | 48.75% | 97.64% | 64.79% | 99.50% |
| SVM-L-5-folds-std | 0.31% | 0.29% | | 4.08% | 0.08% | 2.63% | 0.08% |
| SVM-RBF-5-folds-std | 0.11% | 1.46% | | 0.04% | 4.73% | 0.07% | 0.36% |
| KNN(3k)-5-folds-std | 0.23% | 1.13% | | 10.03% | 0.51% | 7.92% | 0.52% |
| LDA-5-folds-std | 0.37% | 0.20% | | 2.62% | 0.14% | 1.79% | 0.23% |
| NBC-5-folds-std | 1.26% | 0.21% | | 3.11% | 0.59% | 1.88% | 0.22% |
| RF-5-folds-std | 0.11% | 2.78% | | 11.69% | 1.39% | 11.55% | 0.17% |

|  | **AID 886 – DRAMOTE** | |  |  |  |  |  |
| --- | --- | --- | --- | --- | --- | --- | --- |
| Classifier | Sensitivity | Specificity | | Precision | G-Mean | F1-Measure | ROC-AUC |
| SVM-L-5-folds-mean | 79.61% | 99.34% | | 84.63% | 86.10% | 76.13% | 99.80% |
| SVM-RBF-5-folds-mean | 98.94% | 98.57% | | 72.53% | 98.76% | 83.70% | 99.71% |
| KNN(3k)-5-folds-mean | 99.14% | 98.61% | | 73.07% | 98.88% | 84.13% | 99.26% |
| LDA-5-folds-mean | 99.71% | 98.15% | | 67.28% | 98.93% | 80.34% | 99.42% |
| NBC-5-folds-mean | 90.05% | 99.05% | | 79.25% | 94.38% | 83.83% | 95.87% |
| RF-5-folds-mean | 98.82% | 98.90% | | 77.36% | 98.86% | 86.78% | 99.86% |
| SVM-L-5-folds-std | 34.63% | 0.37% | | 7.41% | 24.63% | 25.84% | 0.02% |
| SVM-RBF-5-folds-std | 0.39% | 0.10% | | 1.38% | 0.19% | 0.90% | 0.13% |
| KNN(3k)-5-folds-std | 0.34% | 0.11% | | 1.78% | 0.18% | 1.21% | 0.20% |
| LDA-5-folds-std | 0.35% | 0.11% | | 1.43% | 0.17% | 1.03% | 0.26% |
| NBC-5-folds-std | 6.89% | 0.51% | | 7.41% | 3.34% | 1.50% | 2.92% |
| RF-5-folds-std | 0.32% | 0.11% | | 1.87% | 0.19% | 1.26% | 0.03% |

|  | **AID 899 – No preprocessing** | |  |  |  |  |  |
| --- | --- | --- | --- | --- | --- | --- | --- |
| Classifier | Sensitivity | Specificity | | Precision | G-Mean | F1-Measure | ROC-AUC |
| SVM-L-5-folds-mean | 54.91% | 92.07% | | 67.08% | 71.08% | 60.37% | 83.16% |
| SVM-RBF-5-folds-mean | 37.77% | 97.64% | | 82.35% | 60.70% | 51.77% | 84.66% |
| KNN(3k)-5-folds-mean | 53.33% | 89.09% | | 59.03% | 68.92% | 56.03% | 77.94% |
| LDA-5-folds-mean | 54.17% | 92.73% | | 68.69% | 70.86% | 60.56% | 84.62% |
| NBC-5-folds-mean | 83.18% | 53.60% | | 34.59% | 66.77% | 48.86% | 73.62% |
| RF-5-folds-mean | 51.59% | 95.76% | | 78.15% | 70.26% | 62.13% | 86.71% |
| SVM-L-5-folds-std | 3.26% | 0.67% | | 3.46% | 2.10% | 3.10% | 1.97% |
| SVM-RBF-5-folds-std | 2.60% | 0.44% | | 4.14% | 2.15% | 3.09% | 1.81% |
| KNN(3k)-5-folds-std | 2.83% | 1.10% | | 4.65% | 2.17% | 3.63% | 1.76% |
| LDA-5-folds-std | 2.95% | 0.98% | | 4.81% | 2.15% | 3.52% | 1.67% |
| NBC-5-folds-std | 3.34% | 0.73% | | 2.13% | 1.59% | 2.66% | 2.26% |
| RF-5-folds-std | 3.16% | 0.56% | | 3.68% | 2.23% | 3.23% | 1.18% |

|  | **AID 899 – Random Undersampling** | | | |  |  |  |
| --- | --- | --- | --- | --- | --- | --- | --- |
| Classifier | Sensitivity | Specificity | Precision | G-Mean | | F1-Measure | ROC-AUC |
| SVM-L-5-folds-mean | 76.85% | 74.70% | 47.28% | 75.76% | | 58.52% | 81.24% |
| SVM-RBF-5-folds-mean | 75.09% | 78.13% | 50.33% | 76.58% | | 60.26% | 84.44% |
| KNN(3k)-5-folds-mean | 74.99% | 69.18% | 41.79% | 72.01% | | 53.66% | 78.22% |
| LDA-5-folds-mean | 76.73% | 77.29% | 49.91% | 77.00% | | 60.47% | 84.29% |
| NBC-5-folds-mean | 84.25% | 51.16% | 33.72% | 65.64% | | 48.16% | 72.81% |
| RF-5-folds-mean | 77.99% | 79.38% | 52.75% | 78.68% | | 62.92% | 86.16% |
| SVM-L-5-folds-std | 2.05% | 1.58% | 2.60% | 1.46% | | 2.40% | 1.27% |
| SVM-RBF-5-folds-std | 3.24% | 1.51% | 1.98% | 1.93% | | 2.45% | 1.56% |
| KNN(3k)-5-folds-std | 3.09% | 1.29% | 2.32% | 1.36% | | 2.55% | 1.32% |
| LDA-5-folds-std | 2.28% | 1.05% | 2.53% | 1.56% | | 2.50% | 1.52% |
| NBC-5-folds-std | 2.49% | 0.83% | 1.73% | 0.64% | | 2.09% | 1.37% |
| RF-5-folds-std | 1.58% | 1.21% | 1.27% | 0.95% | | 1.10% | 0.87% |

|  | **AID 899 – SMOTE(200%)** | | |  |  |  |  |
| --- | --- | --- | --- | --- | --- | --- | --- |
| Classifier | Sensitivity | Specificity | Precision | | G-Mean | F1-Measure | ROC-AUC |
| SVM-L-5-folds-mean | 70.68% | 82.80% | 54.80% | | 76.49% | 61.71% | 83.22% |
| SVM-RBF-5-folds-mean | 71.13% | 85.95% | 59.87% | | 78.19% | 65.00% | 86.07% |
| KNN(3k)-5-folds-mean | 72.42% | 72.41% | 43.63% | | 72.41% | 54.45% | 77.08% |
| LDA-5-folds-mean | 72.06% | 82.74% | 55.22% | | 77.21% | 62.48% | 84.83% |
| NBC-5-folds-mean | 80.69% | 52.35% | 33.31% | | 64.97% | 47.15% | 70.37% |
| RF-5-folds-mean | 55.64% | 94.35% | 74.31% | | 72.44% | 63.62% | 86.56% |
| SVM-L-5-folds-std | 2.49% | 1.33% | 4.02% | | 1.77% | 3.33% | 1.38% |
| SVM-RBF-5-folds-std | 2.02% | 0.52% | 2.74% | | 1.25% | 2.30% | 1.46% |
| KNN(3k)-5-folds-std | 2.14% | 0.46% | 2.31% | | 1.12% | 2.37% | 1.44% |
| LDA-5-folds-std | 2.24% | 1.61% | 4.19% | | 1.67% | 3.30% | 1.61% |
| NBC-5-folds-std | 4.28% | 1.05% | 2.50% | | 1.69% | 3.18% | 2.21% |
| RF-5-folds-std | 2.59% | 0.46% | 3.16% | | 1.75% | 2.63% | 1.14% |

|  | **AID 899 – MWMOTE** | |  |  |  |  |  |
| --- | --- | --- | --- | --- | --- | --- | --- |
| Classifier | Sensitivity | Specificity | | Precision | G-Mean | F1-Measure | ROC-AUC |
| SVM-L-5-folds-mean | 70.91% | 80.66% | | 51.94% | 75.62% | 59.94% | 17.94% |
| SVM-RBF-5-folds-mean | 72.52% | 84.27% | | 57.60% | 78.17% | 64.19% | 14.45% |
| KNN(3k)-5-folds-mean | 68.50% | 75.53% | | 45.25% | 71.93% | 54.48% | 76.41% |
| LDA-5-folds-mean | 73.00% | 81.03% | | 53.18% | 76.90% | 61.50% | 84.51% |
| NBC-5-folds-mean | 82.10% | 51.32% | | 33.23% | 64.89% | 47.30% | 70.25% |
| RF-5-folds-mean | 55.98% | 94.36% | | 74.46% | 72.66% | 63.90% | 86.82% |
| SVM-L-5-folds-std | 2.16% | 0.68% | | 2.74% | 1.21% | 2.41% | 1.55% |
| SVM-RBF-5-folds-std | 1.54% | 0.55% | | 2.39% | 1.04% | 1.99% | 1.12% |
| KNN(3k)-5-folds-std | 2.40% | 1.24% | | 3.35% | 1.71% | 3.13% | 1.33% |
| LDA-5-folds-std | 2.59% | 1.55% | | 4.14% | 1.91% | 3.52% | 1.83% |
| NBC-5-folds-std | 3.92% | 1.41% | | 2.50% | 1.81% | 3.09% | 2.27% |
| RF-5-folds-std | 3.08% | 0.15% | | 2.53% | 2.04% | 2.86% | 1.01% |

|  | **AID 899 – GSVM-RU** | |  |  |  |  |  |
| --- | --- | --- | --- | --- | --- | --- | --- |
| Classifier | Sensitivity | Specificity | | Precision | G-Mean | F1-Measure | ROC-AUC |
| SVM-L-5-folds-mean | 97.89% | 15.59% | | 25.54% | 38.53% | 40.48% | 74.37% |
| SVM-RBF-5-folds-mean | 99.83% | 1.70% | | 23.06% | 12.76% | 37.45% | 74.90% |
| KNN(3k)-5-folds-mean | 98.63% | 10.84% | | 24.64% | 32.13% | 39.41% | 61.01% |
| LDA-5-folds-mean | 97.31% | 20.16% | | 26.53% | 43.77% | 41.66% | 75.88% |
| NBC-5-folds-mean | 90.97% | 39.01% | | 30.57% | 59.55% | 45.75% | 68.43% |
| RF-5-folds-mean | 99.11% | 10.24% | | 24.60% | 31.16% | 39.40% | 77.04% |
| SVM-L-5-folds-std | 0.87% | 5.39% | | 1.72% | 6.91% | 2.15% | 3.49% |
| SVM-RBF-5-folds-std | 0.25% | 0.69% | | 1.11% | 2.80% | 1.48% | 4.22% |
| KNN(3k)-5-folds-std | 0.31% | 4.35% | | 1.58% | 6.79% | 2.04% | 4.57% |
| LDA-5-folds-std | 0.96% | 6.67% | | 2.14% | 7.40% | 2.65% | 3.96% |
| NBC-5-folds-std | 1.31% | 2.26% | | 1.71% | 1.87% | 2.01% | 2.58% |
| RF-5-folds-std | 0.58% | 4.32% | | 1.59% | 7.25% | 2.04% | 4.51% |

|  | **AID 899 – DRAMOTE** | |  |  |  |  |  |
| --- | --- | --- | --- | --- | --- | --- | --- |
| Classifier | Sensitivity | Specificity | | Precision | G-Mean | F1-Measure | ROC-AUC |
| SVM-L-5-folds-mean | 65.66% | 79.09% | | 48.09% | 72.01% | 55.46% | 78.62% |
| SVM-RBF-5-folds-mean | 63.17% | 86.43% | | 58.05% | 73.86% | 60.39% | 82.96% |
| KNN(3k)-5-folds-mean | 63.92% | 79.00% | | 47.32% | 71.06% | 54.37% | 76.41% |
| LDA-5-folds-mean | 65.61% | 84.71% | | 55.89% | 74.54% | 60.32% | 83.39% |
| NBC-5-folds-mean | 76.93% | 57.01% | | 34.56% | 66.20% | 47.68% | 70.37% |
| RF-5-folds-mean | 51.79% | 95.64% | | 77.75% | 70.35% | 62.14% | 86.73% |
| SVM-L-5-folds-std | 4.56% | 1.62% | | 3.23% | 2.10% | 3.10% | 1.63% |
| SVM-RBF-5-folds-std | 3.18% | 2.31% | | 5.33% | 1.91% | 3.28% | 1.27% |
| KNN(3k)-5-folds-std | 2.09% | 0.96% | | 2.93% | 1.35% | 2.62% | 1.46% |
| LDA-5-folds-std | 2.13% | 1.26% | | 3.59% | 1.37% | 2.56% | 1.33% |
| NBC-5-folds-std | 3.43% | 1.72% | | 2.50% | 1.66% | 2.93% | 1.41% |
| RF-5-folds-std | 3.29% | 0.63% | | 3.68% | 2.26% | 3.22% | 1.16% |

|  | **AID 938 – No preprocessing** | |  |  |  |  |  |
| --- | --- | --- | --- | --- | --- | --- | --- |
| Classifier | Sensitivity | Specificity | | Precision | G-Mean | F1-Measure | ROC-AUC |
| SVM-L-5-folds-mean | 89.51% | 99.69% | | 89.64% | 94.46% | 89.56% | 99.87% |
| SVM-RBF-5-folds-mean | 93.72% | 99.61% | | 87.67% | 96.62% | 90.59% | 98.56% |
| KNN(3k)-5-folds-mean | 90.65% | 99.66% | | 88.67% | 95.04% | 89.64% | 98.38% |
| LDA-5-folds-mean | 99.55% | 98.26% | | 62.94% | 98.91% | 77.10% | 99.86% |
| NBC-5-folds-mean | 79.69% | 98.68% | | 65.37% | 84.69% | 65.41% | 36.97% |
| RF-5-folds-mean | 94.27% | 99.72% | | 90.75% | 96.95% | 92.47% | 99.80% |
| SVM-L-5-folds-std | 1.80% | 0.07% | | 2.31% | 0.96% | 1.64% | 0.03% |
| SVM-RBF-5-folds-std | 1.89% | 0.07% | | 1.91% | 0.99% | 1.67% | 0.57% |
| KNN(3k)-5-folds-std | 1.56% | 0.04% | | 1.17% | 0.81% | 0.77% | 0.52% |
| LDA-5-folds-std | 0.32% | 0.20% | | 2.31% | 0.19% | 1.75% | 0.06% |
| NBC-5-folds-std | 38.52% | 0.67% | | 3.31% | 28.98% | 26.09% | 16.38% |
| RF-5-folds-std | 1.62% | 0.06% | | 1.69% | 0.85% | 1.46% | 0.18% |

|  | **AID 938 – Random Undersampling** | | | |  |  |  |
| --- | --- | --- | --- | --- | --- | --- | --- |
| Classifier | Sensitivity | Specificity | Precision | G-Mean | | F1-Measure | ROC-AUC |
| SVM-L-5-folds-mean | 99.38% | 98.65% | 68.61% | 99.02% | | 81.15% | 99.72% |
| SVM-RBF-5-folds-mean | 99.55% | 98.63% | 68.16% | 99.09% | | 80.91% | 99.72% |
| KNN(3k)-5-folds-mean | 99.72% | 98.66% | 68.74% | 99.19% | | 81.36% | 99.52% |
| LDA-5-folds-mean | 99.89% | 98.03% | 59.92% | 98.95% | | 74.90% | 99.41% |
| NBC-5-folds-mean | 98.34% | 98.54% | 66.62% | 98.44% | | 79.42% | 99.16% |
| RF-5-folds-mean | 99.83% | 98.44% | 65.50% | 99.13% | | 79.07% | 99.81% |
| SVM-L-5-folds-std | 0.24% | 0.20% | 3.26% | 0.19% | | 2.31% | 0.12% |
| SVM-RBF-5-folds-std | 0.24% | 0.10% | 1.84% | 0.16% | | 1.36% | 0.08% |
| KNN(3k)-5-folds-std | 0.33% | 0.13% | 2.37% | 0.13% | | 1.59% | 0.11% |
| LDA-5-folds-std | 0.15% | 0.14% | 1.75% | 0.11% | | 1.38% | 0.13% |
| NBC-5-folds-std | 0.68% | 0.11% | 1.89% | 0.38% | | 1.45% | 0.21% |
| RF-5-folds-std | 0.15% | 0.17% | 2.94% | 0.16% | | 2.18% | 0.05% |

|  | **AID 938 – SMOTE(50%)** | | |  |  |  |  |
| --- | --- | --- | --- | --- | --- | --- | --- |
| Classifier | Sensitivity | Specificity | Precision | | G-Mean | F1-Measure | ROC-AUC |
| SVM-L-5-folds-mean | 90.74% | 99.68% | 89.42% | | 95.11% | 90.06% | 99.88% |
| SVM-RBF-5-folds-mean | 95.05% | 99.56% | 86.44% | | 97.27% | 90.53% | 98.90% |
| KNN(3k)-5-folds-mean | 94.98% | 99.54% | 85.92% | | 97.23% | 90.21% | 98.85% |
| LDA-5-folds-mean | 99.55% | 98.29% | 63.31% | | 98.92% | 77.39% | 99.87% |
| NBC-5-folds-mean | 75.74% | 98.85% | 65.23% | | 80.71% | 63.37% | 30.58% |
| RF-5-folds-mean | 95.06% | 99.69% | 90.00% | | 97.34% | 92.45% | 99.83% |
| SVM-L-5-folds-std | 1.70% | 0.07% | 2.15% | | 0.89% | 1.46% | 0.03% |
| SVM-RBF-5-folds-std | 2.03% | 0.07% | 2.04% | | 1.06% | 1.76% | 0.50% |
| KNN(3k)-5-folds-std | 1.28% | 0.07% | 1.92% | | 0.65% | 1.20% | 0.35% |
| LDA-5-folds-std | 0.32% | 0.17% | 2.03% | | 0.19% | 1.54% | 0.04% |
| NBC-5-folds-std | 40.35% | 0.61% | 2.03% | | 34.52% | 31.62% | 12.41% |
| RF-5-folds-std | 1.71% | 0.05% | 1.21% | | 0.89% | 1.31% | 0.13% |

|  | **AID 938 – MWMOTE** | |  |  |  |  |  |
| --- | --- | --- | --- | --- | --- | --- | --- |
| Classifier | Sensitivity | Specificity | | Precision | G-Mean | F1-Measure | ROC-AUC |
| SVM-L-5-folds-mean | 77.11% | 99.45% | | 64.58% | 78.27% | NaN | 2.67% |
| SVM-RBF-5-folds-mean | 99.10% | 98.99% | | 74.45% | 99.05% | 85.02% | 0.22% |
| KNN(3k)-5-folds-mean | 97.93% | 99.08% | | 75.77% | 98.50% | 85.43% | 99.21% |
| LDA-5-folds-mean | 99.61% | 98.42% | | 65.11% | 99.01% | 78.69% | 99.53% |
| NBC-5-folds-mean | 93.46% | 98.57% | | 65.94% | 95.97% | 77.31% | 96.91% |
| RF-5-folds-mean | 98.61% | 99.21% | | 78.66% | 98.91% | 87.49% | 99.90% |
| SVM-L-5-folds-std | 43.11% | 0.31% | | 36.14% | 43.76% | NaN | 5.57% |
| SVM-RBF-5-folds-std | 0.58% | 0.09% | | 1.61% | 0.27% | 1.00% | 0.15% |
| KNN(3k)-5-folds-std | 0.94% | 0.07% | | 1.59% | 0.48% | 1.20% | 0.24% |
| LDA-5-folds-std | 0.31% | 0.24% | | 3.94% | 0.11% | 2.84% | 0.28% |
| NBC-5-folds-std | 3.48% | 0.09% | | 1.97% | 1.79% | 2.38% | 2.18% |
| RF-5-folds-std | 0.75% | 0.13% | | 2.64% | 0.40% | 1.74% | 0.02% |

|  | **AID 938 – GSVM-RU** | |  |  |  |  |  |
| --- | --- | --- | --- | --- | --- | --- | --- |
| Classifier | Sensitivity | Specificity | | Precision | G-Mean | F1-Measure | ROC-AUC |
| SVM-L-5-folds-mean | 99.67% | 98.79% | | 70.87% | 99.23% | 82.82% | 99.77% |
| SVM-RBF-5-folds-mean | 99.50% | 1.62% | | 2.90% | 12.34% | 5.64% | 98.78% |
| KNN(3k)-5-folds-mean | 99.39% | 97.12% | | 52.16% | 98.25% | 67.78% | 98.70% |
| LDA-5-folds-mean | 99.33% | 82.39% | | 45.63% | 88.06% | 58.31% | 91.36% |
| NBC-5-folds-mean | 97.38% | 92.01% | | 50.64% | 94.54% | 60.47% | 95.75% |
| RF-5-folds-mean | 99.72% | 93.08% | | 48.67% | 96.23% | 61.55% | 99.64% |
| SVM-L-5-folds-std | 0.45% | 0.14% | | 2.27% | 0.20% | 1.45% | 0.02% |
| SVM-RBF-5-folds-std | 0.45% | 0.90% | | 0.09% | 3.20% | 0.16% | 0.42% |
| KNN(3k)-5-folds-std | 0.53% | 1.04% | | 12.08% | 0.30% | 9.32% | 0.19% |
| LDA-5-folds-std | 0.31% | 33.80% | | 25.70% | 23.12% | 30.02% | 17.98% |
| NBC-5-folds-std | 0.74% | 9.67% | | 33.79% | 4.86% | 33.36% | 4.90% |
| RF-5-folds-std | 0.33% | 9.60% | | 26.29% | 5.12% | 27.22% | 0.14% |

|  | **AID 938 – DRAMOTE** | |  |  |  |  |  |
| --- | --- | --- | --- | --- | --- | --- | --- |
| Classifier | Sensitivity | Specificity | | Precision | G-Mean | F1-Measure | ROC-AUC |
| SVM-L-5-folds-mean | 89.34% | 99.71% | | 90.00% | 94.37% | 89.64% | 99.87% |
| SVM-RBF-5-folds-mean | 95.00% | 99.58% | | 87.00% | 97.00% | 90.55% | 98.75% |
| KNN(3k)-5-folds-mean | 92.44% | 99.60% | | 87.24% | 95.95% | 89.74% | 98.57% |
| LDA-5-folds-mean | 99.60% | 98.33% | | 64.00% | 98.94% | 78.00% | 99.87% |
| NBC-5-folds-mean | 77.00% | 98.83% | | 67.10% | 82.62% | 65.00% | 30.74% |
| RF-5-folds-mean | 95.00% | 99.72% | | 90.78% | 97.10% | 93.00% | 99.85% |
| SVM-L-5-folds-std | 2.40% | 0.06% | | 2.00% | 1.29% | 2.05% | 0.04% |
| SVM-RBF-5-folds-std | 1.90% | 0.07% | | 1.87% | 0.99% | 1.64% | 0.41% |
| KNN(3k)-5-folds-std | 1.71% | 0.06% | | 1.40% | 0.87% | 0.84% | 0.53% |
| LDA-5-folds-std | 0.32% | 0.20% | | 2.24% | 0.22% | 1.73% | 0.05% |
| NBC-5-folds-std | 38.64% | 0.61% | | 2.79% | 30.63% | 28.42% | 12.86% |
| RF-5-folds-std | 1.65% | 0.06% | | 1.61% | 0.86% | 1.48% | 0.09% |

|  | **AID 743042 – No preprocessing** | |  |  |  |  |  |
| --- | --- | --- | --- | --- | --- | --- | --- |
| Classifier | Sensitivity | Specificity | | Precision | G-Mean | F1-Measure | ROC-AUC |
| SVM-L-5-folds-mean | 28.79% | 96.35% | | 40.76% | 52.37% | 33.54% | 23.27% |
| SVM-RBF-5-folds-mean | 0.00% | 100.00% | | 0.00% | 0.00% | 0.00% | 23.53% |
| KNN(3k)-5-folds-mean | 16.25% | 97.12% | | 33.03% | 39.48% | 21.68% | 66.49% |
| LDA-5-folds-mean | 32.02% | 96.49% | | 44.45% | 55.42% | 37.10% | 81.52% |
| NBC-5-folds-mean | 49.97% | 76.11% | | 15.48% | 61.63% | 23.62% | 69.63% |
| RF-5-folds-mean | 15.62% | 99.56% | | 75.50% | 39.30% | 25.83% | 86.62% |
| SVM-L-5-folds-std | 6.59% | 0.63% | | 6.53% | 6.15% | 6.08% | 3.01% |
| SVM-RBF-5-folds-std | 0.00% | 0.00% | | 0.00% | 0.00% | 0.00% | 2.49% |
| KNN(3k)-5-folds-std | 3.91% | 0.51% | | 5.49% | 4.77% | 4.46% | 1.90% |
| LDA-5-folds-std | 5.19% | 0.55% | | 5.06% | 4.62% | 4.84% | 1.77% |
| NBC-5-folds-std | 3.67% | 1.08% | | 0.75% | 2.19% | 1.20% | 1.47% |
| RF-5-folds-std | 2.92% | 0.12% | | 6.81% | 3.69% | 4.30% | 2.16% |

|  | **AID 743042 – Random Undersampling** | | | |  |  |  |
| --- | --- | --- | --- | --- | --- | --- | --- |
| Classifier | Sensitivity | Specificity | Precision | G-Mean | | F1-Measure | ROC-AUC |
| SVM-L-5-folds-mean | 70.73% | 71.62% | 17.93% | 71.13% | | 28.60% | 76.01% |
| SVM-RBF-5-folds-mean | 76.96% | 62.45% | 15.23% | 69.28% | | 25.43% | 77.12% |
| KNN(3k)-5-folds-mean | 71.54% | 64.54% | 15.02% | 67.93% | | 24.82% | 73.27% |
| LDA-5-folds-mean | 71.82% | 75.27% | 20.29% | 73.49% | | 31.62% | 80.36% |
| NBC-5-folds-mean | 57.81% | 69.32% | 14.16% | 63.21% | | 22.74% | 67.98% |
| RF-5-folds-mean | 79.15% | 73.38% | 20.67% | 76.20% | | 32.78% | 83.85% |
| SVM-L-5-folds-std | 4.76% | 1.27% | 1.34% | 2.39% | | 2.07% | 3.00% |
| SVM-RBF-5-folds-std | 4.32% | 2.43% | 0.65% | 1.95% | | 1.06% | 1.32% |
| KNN(3k)-5-folds-std | 2.20% | 1.42% | 0.59% | 0.91% | | 0.74% | 1.41% |
| LDA-5-folds-std | 5.06% | 0.97% | 1.56% | 2.60% | | 2.23% | 2.15% |
| NBC-5-folds-std | 6.29% | 2.38% | 1.09% | 3.54% | | 1.84% | 2.47% |
| RF-5-folds-std | 6.27% | 5.79% | 4.22% | 5.53% | | 5.02% | 3.45% |

|  | **AID 743042 – SMOTE(200%)** | | |  |  |  |  |
| --- | --- | --- | --- | --- | --- | --- | --- |
| Classifier | Sensitivity | Specificity | Precision | | G-Mean | F1-Measure | ROC-AUC |
| SVM-L-5-folds-mean | 40.66% | 92.45% | 32.12% | | 61.10% | 35.73% | 75.42% |
| SVM-RBF-5-folds-mean | 8.20% | 99.26% | 48.69% | | 28.34% | 13.98% | 78.07% |
| KNN(3k)-5-folds-mean | 36.62% | 87.90% | 20.98% | | 56.72% | 26.66% | 72.69% |
| LDA-5-folds-mean | 42.03% | 92.06% | 31.68% | | 62.09% | 36.04% | 79.97% |
| NBC-5-folds-mean | 52.84% | 72.45% | 14.35% | | 61.78% | 22.55% | 68.31% |
| RF-5-folds-mean | 19.91% | 99.40% | 74.10% | | 44.34% | 31.33% | 85.93% |
| SVM-L-5-folds-std | 7.24% | 1.10% | 5.22% | | 5.41% | 5.53% | 2.73% |
| SVM-RBF-5-folds-std | 2.07% | 0.09% | 3.09% | | 3.65% | 3.17% | 1.73% |
| KNN(3k)-5-folds-std | 1.98% | 0.59% | 1.59% | | 1.51% | 1.69% | 1.85% |
| LDA-5-folds-std | 5.24% | 0.81% | 3.12% | | 3.69% | 3.48% | 1.24% |
| NBC-5-folds-std | 5.73% | 1.32% | 0.81% | | 3.14% | 1.48% | 1.00% |
| RF-5-folds-std | 3.60% | 0.11% | 5.99% | | 4.00% | 4.94% | 1.42% |

|  | **AID 743042 – MWMOTE** | | |  |  |  |  |
| --- | --- | --- | --- | --- | --- | --- | --- |
| Classifier | Sensitivity | Specificity | Precision | | G-Mean | F1-Measure | ROC-AUC |
| SVM-L-5-folds-mean | 44.30% | 91.94% | 32.51% | | 63.66% | 37.42% | 23.76% |
| SVM-RBF-5-folds-mean | 15.20% | 98.27% | 43.67% | | 38.60% | 22.53% | 21.23% |
| KNN(3k)-5-folds-mean | 35.50% | 89.47% | 22.82% | | 56.34% | 27.76% | 66.29% |
| LDA-5-folds-mean | 45.26% | 92.94% | 35.92% | | 64.72% | 39.99% | 81.02% |
| NBC-5-folds-mean | 52.63% | 75.76% | 15.96% | | 63.08% | 24.48% | 69.85% |
| RF-5-folds-mean | 20.27% | 99.17% | 68.38% | | 44.74% | 31.18% | 86.75% |
| SVM-L-5-folds-std | 6.70% | 0.81% | 4.35% | | 4.83% | 4.93% | 3.65% |
| SVM-RBF-5-folds-std | 1.56% | 0.29% | 6.01% | | 2.01% | 2.43% | 1.41% |
| KNN(3k)-5-folds-std | 1.98% | 0.69% | 1.27% | | 1.51% | 1.29% | 1.71% |
| LDA-5-folds-std | 6.45% | 0.67% | 3.40% | | 4.60% | 4.38% | 2.25% |
| NBC-5-folds-std | 5.13% | 1.16% | 0.92% | | 2.82% | 1.54% | 2.78% |
| RF-5-folds-std | 1.91% | 0.09% | 0.51% | | 1.02% | 1.15% | 0.44% |

|  | **AID 743042 – GSVM-RU** | | |  |  |  |  |
| --- | --- | --- | --- | --- | --- | --- | --- |
| Classifier | Sensitivity | Specificity | Precision | | G-Mean | F1-Measure | ROC-AUC |
| SVM-L-5-folds-mean | 97.44% | 31.57% | 11.11% | | 55.27% | 19.95% | 76.26% |
| SVM-RBF-5-folds-mean | 97.17% | 26.84% | 10.44% | | 51.00% | 18.84% | 73.96% |
| KNN(3k)-5-folds-mean | 95.19% | 33.87% | 11.21% | | 56.72% | 20.06% | 69.74% |
| LDA-5-folds-mean | 93.61% | 37.57% | 11.68% | | 59.01% | 20.76% | 77.12% |
| NBC-5-folds-mean | 77.85% | 49.29% | 11.91% | | 61.83% | 20.65% | 66.10% |
| RF-5-folds-mean | 97.98% | 24.97% | 10.28% | | 49.28% | 18.61% | 78.97% |
| SVM-L-5-folds-std | 2.28% | 5.41% | 0.21% | | 4.11% | 0.34% | 3.57% |
| SVM-RBF-5-folds-std | 1.75% | 3.06% | 0.57% | | 3.03% | 0.95% | 3.21% |
| KNN(3k)-5-folds-std | 1.25% | 3.57% | 0.51% | | 3.14% | 0.83% | 2.35% |
| LDA-5-folds-std | 2.45% | 7.77% | 0.59% | | 5.44% | 0.90% | 3.04% |
| NBC-5-folds-std | 3.99% | 5.50% | 0.81% | | 3.41% | 1.32% | 2.17% |
| RF-5-folds-std | 1.46% | 4.86% | 0.42% | | 4.62% | 0.70% | 2.39% |

|  | **AID 743042 – DRAMOTE** | | |  |  |  |  |
| --- | --- | --- | --- | --- | --- | --- | --- |
| Classifier | Sensitivity | Specificity | Precision | | G-Mean | F1-Measure | ROC-AUC |
| SVM-L-5-folds-mean | 42.45% | 92.36% | 32.73% | | 62.44% | 36.87% | 76.52% |
| SVM-RBF-5-folds-mean | 15.47% | 98.65% | 50.01% | | 38.95% | 23.61% | 77.65% |
| KNN(3k)-5-folds-mean | 43.59% | 87.10% | 22.94% | | 61.55% | 30.03% | 70.49% |
| LDA-5-folds-mean | 39.75% | 93.92% | 36.45% | | 60.97% | 37.92% | 81.13% |
| NBC-5-folds-mean | 50.79% | 76.78% | 16.03% | | 62.32% | 24.35% | 68.43% |
| RF-5-folds-mean | 20.26% | 99.39% | 73.96% | | 44.78% | 31.79% | 85.94% |
| SVM-L-5-folds-std | 6.92% | 0.73% | 4.36% | | 5.12% | 4.97% | 3.60% |
| SVM-RBF-5-folds-std | 2.67% | 0.20% | 8.76% | | 3.35% | 3.99% | 2.20% |
| KNN(3k)-5-folds-std | 4.71% | 1.29% | 2.97% | | 3.20% | 3.56% | 2.13% |
| LDA-5-folds-std | 5.46% | 0.75% | 4.05% | | 4.19% | 4.09% | 2.66% |
| NBC-5-folds-std | 7.31% | 1.48% | 1.44% | | 4.43% | 2.46% | 1.49% |
| RF-5-folds-std | 3.07% | 0.12% | 5.66% | | 3.31% | 4.11% | 0.92% |

|  | **AID 743288 – No preprocessing** | |  |  |  |  |  |
| --- | --- | --- | --- | --- | --- | --- | --- |
| Classifier | Sensitivity | Specificity | | Precision | G-Mean | F1-Measure | ROC-AUC |
| SVM-L-5-folds-mean | 32.37% | 97.59% | | 33.93% | 55.67% | 32.93% | 35.33% |
| SVM-RBF-5-folds-mean | 0.00% | 100.00% | | 0.00% | 0.00% | 0.00% | 36.94% |
| KNN(3k)-5-folds-mean | 9.69% | 99.24% | | 38.19% | 27.46% | 0.00% | 62.25% |
| LDA-5-folds-mean | 23.57% | 98.16% | | 31.81% | 47.13% | 26.57% | 81.89% |
| NBC-5-folds-mean | 58.37% | 82.46% | | 11.32% | 69.12% | 18.93% | 76.01% |
| RF-5-folds-mean | 8.86% | 99.86% | | 75.00% | 28.59% | 15.26% | 85.53% |
| SVM-L-5-folds-std | 10.60% | 0.53% | | 7.06% | 8.77% | 8.27% | 23.08% |
| SVM-RBF-5-folds-std | 0.00% | 0.00% | | 0.00% | 0.00% | 0.00% | 33.93% |
| KNN(3k)-5-folds-std | 6.33% | 0.51% | | 29.39% | 16.20% | 0.00% | 4.49% |
| LDA-5-folds-std | 11.54% | 0.35% | | 5.80% | 10.58% | 9.08% | 4.21% |
| NBC-5-folds-std | 10.99% | 1.83% | | 1.75% | 7.17% | 2.96% | 7.45% |
| RF-5-folds-std | 6.28% | 0.13% | | 25.00% | 9.13% | 9.26% | 4.32% |

|  | **AID 743288 – Random Undersampling** | | | |  |  |  |
| --- | --- | --- | --- | --- | --- | --- | --- |
| Classifier | Sensitivity | Specificity | Precision | G-Mean | | F1-Measure | ROC-AUC |
| SVM-L-5-folds-mean | 71.50% | 72.96% | 9.48% | 72.09% | | 16.70% | 77.06% |
| SVM-RBF-5-folds-mean | 70.19% | 68.21% | 7.84% | 68.24% | | 14.08% | 75.27% |
| KNN(3k)-5-folds-mean | 70.33% | 67.61% | 7.68% | 68.74% | | 13.83% | 73.18% |
| LDA-5-folds-mean | 69.66% | 74.89% | 9.82% | 71.87% | | 17.19% | 79.24% |
| NBC-5-folds-mean | 56.88% | 70.78% | 7.09% | 63.20% | | 12.58% | 68.79% |
| RF-5-folds-mean | 69.97% | 70.34% | 8.38% | 69.88% | | 14.97% | 76.52% |
| SVM-L-5-folds-std | 10.79% | 3.20% | 2.82% | 6.68% | | 4.58% | 8.01% |
| SVM-RBF-5-folds-std | 21.38% | 7.20% | 1.95% | 10.67% | | 3.54% | 7.82% |
| KNN(3k)-5-folds-std | 12.26% | 2.57% | 0.85% | 6.12% | | 1.56% | 7.20% |
| LDA-5-folds-std | 14.27% | 2.76% | 2.92% | 7.74% | | 4.85% | 7.46% |
| NBC-5-folds-std | 12.71% | 4.00% | 1.99% | 8.50% | | 3.39% | 10.00% |
| RF-5-folds-std | 10.71% | 4.26% | 1.29% | 4.60% | | 2.29% | 5.63% |

|  | **AID 743288 – SMOTE(200%)** | | |  |  |  |  |
| --- | --- | --- | --- | --- | --- | --- | --- |
| Classifier | Sensitivity | Specificity | Precision | | G-Mean | F1-Measure | ROC-AUC |
| SVM-L-5-folds-mean | 38.05% | 94.85% | 20.98% | | 58.50% | 26.72% | 70.62% |
| SVM-RBF-5-folds-mean | 2.58% | 99.57% | 10.00% | | 10.13% | NaN | 79.37% |
| KNN(3k)-5-folds-mean | 15.13% | 96.60% | 15.61% | | 33.85% | NaN | 65.76% |
| LDA-5-folds-mean | 36.70% | 96.93% | 30.88% | | 58.39% | 32.68% | 80.81% |
| NBC-5-folds-mean | 49.79% | 84.96% | 11.12% | | 64.49% | 18.13% | 71.07% |
| RF-5-folds-mean | 12.20% | 99.81% | 73.33% | | 34.46% | 20.72% | 84.65% |
| SVM-L-5-folds-std | 18.50% | 0.70% | 6.53% | | 15.03% | 10.05% | 6.00% |
| SVM-RBF-5-folds-std | 3.54% | 0.26% | 13.69% | | 13.87% | NaN | 8.38% |
| KNN(3k)-5-folds-std | 10.32% | 1.16% | 11.70% | | 20.04% | NaN | 5.56% |
| LDA-5-folds-std | 15.45% | 0.95% | 7.92% | | 13.31% | 10.08% | 4.06% |
| NBC-5-folds-std | 13.39% | 1.77% | 1.68% | | 8.97% | 3.16% | 7.83% |
| RF-5-folds-std | 4.78% | 0.20% | 25.00% | | 6.59% | 7.15% | 5.26% |

|  | **AID 743288 – MWMOTE** | | |  |  |  |  |
| --- | --- | --- | --- | --- | --- | --- | --- |
| Classifier | Sensitivity | Specificity | Precision | | G-Mean | F1-Measure | ROC-AUC |
| SVM-L-5-folds-mean | 35.79% | 97.68% | 36.78% | | 58.53% | 35.87% | 25.20% |
| SVM-RBF-5-folds-mean | 2.33% | 99.81% | NaN | | 9.62% | NaN | 19.66% |
| KNN(3k)-5-folds-mean | 18.06% | 95.93% | 14.74% | | 41.22% | 16.18% | 62.03% |
| LDA-5-folds-mean | 30.00% | 97.92% | 35.91% | | 53.15% | 32.19% | 81.95% |
| NBC-5-folds-mean | 48.28% | 87.37% | 12.67% | | 63.91% | 19.98% | 77.27% |
| RF-5-folds-mean | 8.36% | 99.72% | 65.00% | | 28.66% | 14.54% | 82.24% |
| SVM-L-5-folds-std | 11.17% | 0.39% | 4.89% | | 9.20% | 7.44% | 7.12% |
| SVM-RBF-5-folds-std | 3.25% | 0.20% | NaN | | 13.23% | NaN | 6.81% |
| KNN(3k)-5-folds-std | 5.52% | 0.69% | 4.54% | | 6.44% | 4.90% | 4.28% |
| LDA-5-folds-std | 11.69% | 0.65% | 13.82% | | 11.89% | 11.72% | 4.11% |
| NBC-5-folds-std | 18.79% | 2.51% | 3.43% | | 12.35% | 5.77% | 8.88% |
| RF-5-folds-std | 3.50% | 0.20% | 27.64% | | 5.56% | 5.88% | 3.20% |

|  | **AID 743288 – GSVM-RU** | | |  |  |  |  |
| --- | --- | --- | --- | --- | --- | --- | --- |
| Classifier | Sensitivity | Specificity | Precision | | G-Mean | F1-Measure | ROC-AUC |
| SVM-L-5-folds-mean | 91.82% | 38.46% | 5.48% | | 58.84% | 10.34% | 77.71% |
| SVM-RBF-5-folds-mean | 92.31% | 44.32% | 6.03% | | 63.83% | 11.32% | 71.99% |
| KNN(3k)-5-folds-mean | 87.40% | 47.03% | 5.97% | | 63.96% | 11.18% | 71.63% |
| LDA-5-folds-mean | 79.46% | 48.84% | 5.75% | | 61.29% | 10.71% | 74.19% |
| NBC-5-folds-mean | 77.15% | 55.08% | 6.19% | | 64.93% | 11.45% | 67.95% |
| RF-5-folds-mean | 89.81% | 35.13% | 5.13% | | 54.85% | 9.69% | 74.75% |
| SVM-L-5-folds-std | 9.02% | 9.97% | 0.51% | | 7.45% | 0.91% | 8.21% |
| SVM-RBF-5-folds-std | 8.48% | 7.75% | 0.45% | | 7.53% | 0.82% | 7.68% |
| KNN(3k)-5-folds-std | 10.10% | 5.53% | 0.44% | | 5.60% | 0.80% | 6.73% |
| LDA-5-folds-std | 16.28% | 11.90% | 1.17% | | 7.51% | 2.16% | 8.24% |
| NBC-5-folds-std | 14.52% | 1.78% | 1.00% | | 5.70% | 1.86% | 4.28% |
| RF-5-folds-std | 11.14% | 13.25% | 0.48% | | 8.61% | 0.89% | 4.61% |

|  | **AID 743288 – DRAMOTE** | | |  |  |  |  |
| --- | --- | --- | --- | --- | --- | --- | --- |
| Classifier | Sensitivity | Specificity | Precision | | G-Mean | F1-Measure | ROC-AUC |
| SVM-L-5-folds-mean | 30.52% | 97.26% | 30.39% | | 54.27% | 30.20% | 73.59% |
| SVM-RBF-5-folds-mean | 3.92% | 99.67% | NaN | | 12.26% | NaN | 77.12% |
| KNN(3k)-5-folds-mean | 31.19% | 94.89% | 19.26% | | 54.10% | 23.72% | 66.22% |
| LDA-5-folds-mean | 28.48% | 98.16% | 38.33% | | 51.84% | 31.86% | 82.55% |
| NBC-5-folds-mean | 45.01% | 89.55% | 14.10% | | 63.06% | 21.38% | 74.76% |
| RF-5-folds-mean | 14.71% | 99.81% | 76.67% | | 38.14% | 24.44% | 83.30% |
| SVM-L-5-folds-std | 5.79% | 0.59% | 6.68% | | 5.25% | 5.42% | 7.28% |
| SVM-RBF-5-folds-std | 5.92% | 0.36% | NaN | | 17.27% | NaN | 10.27% |
| KNN(3k)-5-folds-std | 7.71% | 1.12% | 4.29% | | 6.77% | 5.11% | 9.92% |
| LDA-5-folds-std | 11.71% | 0.73% | 16.02% | | 11.60% | 11.44% | 3.83% |
| NBC-5-folds-std | 11.21% | 1.46% | 2.22% | | 8.11% | 3.66% | 6.13% |
| RF-5-folds-std | 8.02% | 0.11% | 14.97% | | 3.60% | 2.83% | 5.34% |
