# Supplementary material for: Mining Chemical Activity Status from High-Throughput Screening Assays
Source: PLoS One. 2015 Dec 14;10(12):e0144426. doi: 10.1371/journal.pone.0144426 (PMC4682830; doi:10.1371/journal.pone.0144426)
Supplement: S1 Text — The file also includes most of the features we selected after applying variable selection over the originals set of generated features. (DOCX) [file pone.0144426.s004.docx]

# **Mining chemical activity status in high-throughput screening assays**

*Othman Soufan^1^, Wail Ba-alawi^1^, Moataz Afeef^1^, Magbubah Essack^1^****,*** *Valentin Rodionov^2^,* *Panos Kalnis^3^ and Vladimir B. Bajic^1,*^*

^1^King Abdullah University of Science and Technology (KAUST), Computational Bioscience Research Center (CBRC), Thuwal 23955-6900, Saudi Arabia. ^2^King Abdullah University of Science and Technology (KAUST), KAUST Catalysis Center (KCC), Thuwal 23955-6900, Saudi Arabia. ^3^King Abdullah University of Science and Technology (KAUST), Infocloud Group, Computer, Electrical and Mathematical Sciences and Engineering Division (CEMSE), Thuwal 23955-6900, Saudi Arabia.

# **Supporting Information Text 1**

**A summary description of features generated for chemical compounds. The file also includes most of the features we selected after applying variable selection over the originals set of generated features.**

**1**. **Feature Generation**

Using OpenBabel, we extracted the following groups of features from SMILES strings obtained from PubChem:

• **Group 1: SMARTS patterns (476 features)**

SMARTS patterns specified in the file patterns.txt provided by OpenBabel and available from our download link below.

Some examples are:

• [#6,#1]C([#6,#1])([OH])([OH]) 17 carbonyl hydrate

• [#6,#1]C([#6,#1])([OH])(O[#6]) 18 hemiacetal

• [#6,#1]C([#6,#1])(O[#6])(O[#6]) 19 acetal

• [#6,#1]C([#6,#1])(N([#6,#1])[#6,#1])(O[#6]) 20 hemiaminal

• [#6,#1]C([#6,#1])(N([#6,#1])[#6,#1])(N([#6,#1])[#6,#1]) 21 aminal

• [#6,#1]C([#6,#1])(N([#6,#1])[#6,#1])([S][#6]) 22 thiohemiaminal

• **Group 2: SMARTS patterns (307 features)**

SMARTS patterns specified in the file SMARTS_InteLigand.txt provided by OpenBabel and available from our download link below.

Some examples are:

• Diaryl ether: [c][OX2][c]

• Alkylarylthioether: [SX2](c)[CX4;!$(C([OX2])[O,S,#7,#15,F,Cl,Br,I])]

• Diaryl thioether: [c][SX2][c]

• **Group 3: Spectrophore descriptor (48 features)**

Spectrophore descriptors (developed by Silicos NV) are one-dimensional descriptors that allow for a simplified accounting for the shape and surface properties of molecules. We used the Spectrophore code as provided by OpenBabel..

In addition to OpenBabel, we have used the RDKit shemoinformatics toolkit. The following groups of features were generated using it:

• **Group 1: Topological Fingerprints (1024 features)**

• **Group 2: MACCS Keys (166 features) (SMARTS-based implementation).**

• **Group 3: Chemical Descriptors (37 features), including but not limited to:**

• Molecular weight

• Number of H-bond acceptors and donors

• LogP

• Heavy atom count

• -NH and -OH count

• Number of valence electrons

• Number of rotatable bonds

Finally, we used the **PubChem 881 dictionary fingerprint** features. Finally, we ended up with a feature vector of **2940** features for every molecule. While some features obtained in this fashion are certainly duplicated, or have a strong correlation, a good feature selection algorithm would exclude any redundant or irrelevant information.

# **2. Feature Selection: The list of features we selected out of the original generated ones.**

PubChem FingerPrint

1. '>= 8 H'
2. '>= 32 H'
3. '>= 1 B'
4. '>= 32 C'
5. '>= 16 O'
6. '>= 2 F'
7. '>= 1 Na'
8. '>= 2 Na'
9. '>= 1 P'
10. '>= 4 P'
11. '>= 8 S'
12. '>= 2 Cl'
13. '>= 4 Cl'
14. '>= 2 Br'
15. '>= 4 Br'
16. '>= 1 I'
17. '>= 2 I'
18. '>= 1 Al'
19. '>= 1 Ca'
20. '>= 1 Sc'
21. '>= 1 Ni'
22. '>= 1 Se'
23. '>= 1 Sr'
24. '>= 1 Y'
25. '>= 1 In'
26. '>= 1 Sb'
27. '>= 1 Te'
28. '>= 1 Lu'
29. '>= 1 Ta'
30. '>= 1 W'
31. '>= 1 Ir'
32. '>= 1 Au'
33. '>= 1 Pb'
34. '>= 1 Eu'
35. '>= 1 any ring size 3'
36. '>= 1 saturated or aromatic carbon-only ring size 3'
37. '>= 1 saturated or aromatic heteroatom-containing ring size 3'
38. '>= 1 unsaturated non-aromatic nitrogen-containing ring size 3'
39. '>= 2 saturated or aromatic heteroatom-containing ring size 3'
40. '>= 2 saturated or aromatic heteroatom-containing ring size 4'
41. '>= 2 unsaturated non-aromatic nitrogen-containing ring size 4'
42. '>= 2 unsaturated non-aromatic heteroatom-containing ring size 4'
43. '>= 1 saturated or aromatic nitrogen-containing ring size 5'
44. '>= 1 unsaturated non-aromatic nitrogen-containing ring size 5'
45. '>= 1 unsaturated non-aromatic heteroatom-containing ring size 5'
46. '>= 2 saturated or aromatic carbon-only ring size 5'
47. '>= 2 unsaturated non-aromatic carbon-only ring size 5'
48. '>= 3 unsaturated non-aromatic carbon-only ring size 5'
49. '>= 4 saturated or aromatic nitrogen-containing ring size 5'
50. '>= 4 saturated or aromatic heteroatom-containing ring size 5'
51. '>= 4 unsaturated non-aromatic carbon-only ring size 5'
52. '>= 4 unsaturated non-aromatic heteroatom-containing ring size 5'
53. '>= 5 any ring size 5'
54. '>= 5 unsaturated non-aromatic carbon-only ring size 5'
55. '>= 5 unsaturated non-aromatic heteroatom-containing ring size 5'
56. '>= 1 any ring size 6'
57. '>= 2 any ring size 6'
58. '>= 2 saturated or aromatic nitrogen-containing ring size 6'
59. '>= 2 unsaturated non-aromatic carbon-only ring size 6'
60. '>= 3 saturated or aromatic carbon-only ring size 6'
61. '>= 3 unsaturated non-aromatic carbon-only ring size 6'
62. '>= 3 unsaturated non-aromatic nitrogen-containing ring size 6'
63. '>= 4 unsaturated non-aromatic heteroatom-containing ring size 6'
64. '>= 5 any ring size 6'
65. '>= 5 saturated or aromatic carbon-only ring size 6'
66. '>= 5 saturated or aromatic nitrogen-containing ring size 6'
67. '>= 5 saturated or aromatic heteroatom-containing ring size 6'
68. '>= 5 unsaturated non-aromatic heteroatom-containing ring size 6'
69. '>= 1 any ring size 7'
70. '>= 1 saturated or aromatic nitrogen-containing ring size 7'
71. '>= 1 saturated or aromatic heteroatom-containing ring size 7'
72. '>= 1 saturated or aromatic carbon-only ring size 8'
73. '>= 1 unsaturated non-aromatic carbon-only ring size 8'
74. '>= 1 unsaturated non-aromatic heteroatom-containing ring size 8'
75. '>= 1 any ring size 9'
76. '>= 1 saturated or aromatic heteroatom-containing ring size 9'
77. '>= 1 unsaturated non-aromatic nitrogen-containing ring size 9'
78. 'Li-S'
79. 'Li-Cl'
80. 'B-H'
81. 'B-C'
82. 'B-N'
83. 'B-F'
84. 'B-P'
85. 'B-Cl'
86. 'B-Br'
87. 'C-C'
88. 'C-S'
89. 'C-Br'
90. 'N-F'
91. 'N-Si'
92. 'N-P'
93. 'N-Br'
94. 'O-Mg'
95. 'O-P'
96. 'Al-H'
97. 'As-H'
98. 'C(~Br)(~H)'
99. 'C(~C)(~C)(~C)(~O)'
100. 'C(~C)(~Cl)'
101. 'C(~C)(~H)'
102. 'C(~C)(~H)(~S)'
103. 'C(~C)(~Si)'
104. 'C(~C)(:N)'
105. 'C(~C)(:N)(:N)'
106. 'C(~Cl)(~Cl)'
107. 'C(~Cl)(~H)'
108. 'C(~Cl)(:C)'
109. 'C(~F)(~F)'
110. 'C(~F)(:C)'
111. 'C(~H)(~S)'
112. 'C(~H)(:C)'
113. 'C(~H)(:N)'
114. 'C(~H)(~H)(~H)'
115. 'C(~N)(~N)'
116. 'C(~N)(:C)'
117. 'C(~N)(:C)(:N)'
118. 'C(:C)(:C)(:N)'
119. 'C(:C)(:N)(:N)'
120. 'C(:N)(:N)'
121. 'N(~C)(~H)'
122. 'N(~C)(~H)(~N)'
123. 'N(~C)(~O)'
124. 'N(~H)(:C)'
125. 'N(:C)(:C)'
126. 'O(~C)(~P)'
127. 'C#N'
128. 'N=O'
129. 'C(-C)(-C)(=C)'
130. 'C(-C)(-H)(=C)'
131. 'C(-C)(-N)(=O)'
132. 'C(-C)(-O)(=O)'
133. 'C(-C)(=C)'
134. 'C(-O)(=O)'
135. 'N(-C)(=O)'
136. 'P(-O)(=O)'
137. 'S(-O)(=O)'
138. 'N#C-C=C'
139. 'C=N-N-C'
140. 'C:C-C=C'
141. 'S:C:C:C'
142. 'C:N:C-C'
143. 'S-C:N:C'
144. 'S:C:C:N'
145. 'N-N-C:C'
146. 'S-C-S-C'
147. 'C:N-C:C'
148. 'O=C-C:C'
149. 'N-C=C-[#1]'
150. 'S=C-N-[#1]'
151. 'S:C:C-[#1]'
152. 'C=C-C:C'
153. 'C-N-N-[#1]'
154. '[As]-C:C-[#1]'
155. '[#1]-N-C-[#1]'
156. 'N:C-C:C'
157. 'O=C-C-C'
158. 'N=C-C-[#1]'
159. 'Cl-C-C-O'
160. 'C=C-C-C'
161. 'N-C-N-C'
162. 'O-C-O-C'
163. 'O-C-C:C-C'
164. 'N=C-C:C-[#1]'
165. 'C-C-C-C-C'
166. 'Cl-C:C-O-C'
167. 'Cl-C-C-C-C'
168. 'N-C-C-C-C'
169. 'Cl-C:C:C-C'
170. 'C-C-C-O-[#1]'
171. 'C:C-C-C:C'
172. 'O-C-C=C-C'
173. 'S-C:C:C-N'
174. 'O-C:C-O-[#1]'
175. '[#1]-C-C-N-[#1]'
176. 'O=C-N-C-C'
177. 'O=N-C:C-N'
178. 'O-C:C:C-N'
179. 'C-C=C-C-C'
180. 'O-C-O-C-C'
181. 'Br-C-C-C:C'
182. 'C-C=C-C:C'
183. 'C-C-C=C-C'
184. 'O=C-C-C-C-C'
185. 'O=C-C-C-C-N'
186. 'O=C-C-C-C=O'
187. 'O-C-C-C-C-C-C'
188. 'O-C-C-C-C-C-O'
189. 'O=C-C-C-C-C-O'
190. 'O=C-C-C-C-C=O'
191. 'C-C-C-C-C-C-C-C'
192. 'C-C-C-C-C-C(C)-C'
193. 'O-C-C-C-C-C-C-C'
194. 'O-C-C-C-C-C(C)-C'
195. 'O-C-C-C-C-C-O-C'
196. 'O-C-C-C-C-C-N-C'
197. 'O-C-C-C-C-C(N)-C'
198. 'O=C-C-C-C-C(=O)-C'
199. 'Cc1ccc(S)cc1'
200. 'Cc1ccc(N)cc1'
201. 'Cc1ccc(Cl)cc1'
202. 'Cc1ccc(Br)cc1'
203. 'Sc1ccc(Cl)cc1'
204. 'Nc1ccc(N)cc1'
205. 'Cc1cc(S)ccc1'
206. 'Cc1cc(N)ccc1'
207. 'Cc1cc(Cl)ccc1'
208. 'Oc1cc(N)ccc1'
209. 'Sc1cc(N)ccc1'
210. 'Sc1cc(Cl)ccc1'
211. 'Clc1cc(Br)ccc1'
212. 'Cc1c(N)cccc1'
213. 'Oc1c(S)cccc1'
214. 'Oc1c(Cl)cccc1'
215. 'Sc1c(Cl)cccc1'
216. 'CC1CCC(Br)CC1'
217. 'SC1CCC(N)CC1'
218. 'SC1CCC(Cl)CC1'
219. 'ClC1CCC(Br)CC1'
220. 'OC1CC(N)CCC1'
221. 'SC1CC(S)CCC1'
222. 'SC1CC(Br)CCC1'
223. 'NC1CC(Cl)CCC1'
224. 'CC1C(Cl)CCCC1'
225. 'OC1C(O)CCCC1'
226. 'OC1C(S)CCCC1'
227. 'NC1C(N)CCCC1'
228. 'NC1C(Br)CCCC1'
229. 'ClC1C(Br)CCCC1'
230. 'CC1CC(Cl)CC1'
231. 'OC1CC(S)CC1'
232. 'SC1CC(Cl)CC1'
233. 'NC1CC(N)CC1'
234. 'BrC1CC(Br)CC1'
235. 'CC1C(C)CCC1'
236. 'OC1C(Br)CCC1'
237. 'ClC1C(Cl)CCC1'

'BrC1C(Br)CCC1'

OpenBabel SMART Patterns (FP3)

1. anion
2. aldehyde or ketone
3. oxime
4. thiohemiaminal
5. enol
6. enol ether
7. primary alcohol
8. tertiary alcohol
9. aminoalcohol
10. ether
11. dialkyl ether
12. alkylaryl ether
13. peroxide
14. aryl
15. HBD
16. Ring
17. nitrile
18. urea

OpenBabel SMART Patterns (FP4)

1. 'Quaternary_carbon: [CX4]([#6])([#6])([#6])[#6]'
2. 'Alkene: [CX3;$([H2]),$([H1][#6]),$(C([#6])[#6])]=[CX3;$([H2]),$([H1][#6]),$(C([#6])[#6])] '
3. 'Allene: [CX3]=[CX2]=[CX3]'
4. 'Alkylfluoride: [FX1][CX4]'
5. 'Alkylbromide: [BrX1][CX4]'
6. 'Dialkylether: [OX2]([CX4;!$(C([OX2])[O,S,#7,#15,F,Cl,Br,I])])[CX4;!$(C([OX2])[O,S,#7,#15])]'
7. 'Dialkylthioether: [SX2]([CX4;!$(C([OX2])[O,S,#7,#15,F,Cl,Br,I])])[CX4;!$(C([OX2])[O,S,#7,#15])]'
8. 'Alkylarylether: [OX2](c)[CX4;!$(C([OX2])[O,S,#7,#15,F,Cl,Br,I])]'
9. 'Diarylether: [c][OX2][c]'
10. 'Oxonium: [O+;!$([O]~[!#6]);!$([S]*~[#7,#8,#15,#16])]'
11. 'Primary_arom_amine: [NX3H2+0,NX4H3+]c'
12. 'Tertiary_mixed_amine: [NX3H0+0,NX4H1+;$([N]([c])([C])[#6]);!$([N]*~[#7,#8,#15,#16])]'
13. 'Ammonium: [N+;!$([N]~[!#6]);!$(N=*);!$([N]*~[#7,#8,#15,#16])]'
14. 'Alkylthiol: [SX2H][CX4;!$(C([SX2H])~[O,S,#7,#15])]'
15. 'Alkylarylthioether: [SX2](c)[CX4;!$(C([SX2])[O,S,#7,#15])]'
16. 'Peroxo: [OX2D2][OX2D2]'
17. 'Thioacetal: [SX2]([#6;!$(C=[O,S,N])])[CX4;!$(C(S)(S)[!#6])][SX2][#6;!$(C=[O,S,N])]'
18. 'Acetal_like: [NX3v3,SX2,OX2;!$(*C=[#7,#8,#15,#16])][CX4;!$(C([N,S,O])([N,S,O])[!#6])][FX1,ClX1,BrX1,IX1,NX3v3,SX2,OX2;!$(*C=[#7,#8,#15,#16])]'
19. 'Hetero_methylen_ester_and_similar: [NX3v3,SX2,OX2;$(**=[#7,#8,#15,#16])][CX4;!$(C([N,S,O])([N,S,O])[!#6])][FX1,ClX1,BrX1,IX1,NX3v3,SX2,OX2;!$(*C=[#7,#8,#15,#16])]'
20. 'Chloroalkene: [ClX1][CX3]=[CX3]'
21. 'Fluoroalkene: [FX1][CX3]=[CX3]'
22. 'Enolester: [OX2]([CX3]=[OX1])[#6X3;$([#6][#6]),$([H1])]=[#6X3;!$(C[OX2H])]'
23. 'Acylchloride: [CX3;$([R0][#6]),$([H1R0])](=[OX1])[ClX1] '
24. 'Acylbromide: [CX3;$([R0][#6]),$([H1R0])](=[OX1])[BrX1] '
25. 'Carbothioic_acid: [CX3;!R;$([C][#6]),$([CH]);$([C](=[OX1])[$([SX2H]),$([SX1-])]),$([C](=[SX1])[$([OX2H]),$([OX1-])])]'
26. 'Carbothioic_S_ester: [CX3;$([R0][#6]),$([H1R0])](=[OX1])[SX2][#6;!$(C=[O,N,S])]'
27. 'Carbodithioic_acid: [CX3;!R;$([C][#6]),$([CH]);$([C](=[SX1])[SX2H])]'
28. 'Tertiary_amide: [CX3;$([R0][#6]),$([H1R0])](=[OX1])[#7X3H0]([#6;!$(C=[O,N,S])])[#6;!$(C=[O,N,S])]'
29. 'Lactam: [#6R][#6X3R](=[OX1])[#7X3;$([H1][#6;!$(C=[O,N,S])]),$([H0]([#6;!$(C=[O,N,S])])[#6;!$(C=[O,N,S])])]'
30. 'N_hetero_imide: [#6X3;$([H0][#6]),$([H1])](=[OX1])[#7X3H0]([!#6])[#6X3;$([H0][#6]),$([H1])](=[OX1])'
31. 'Imide_acidic: [#6X3;$([H0][#6]),$([H1])](=[OX1])[#7X3H1][#6X3;$([H0][#6]),$([H1])](=[OX1])'
32. 'Oximester: [#6X3;$([H0][#6]),$([H1])](=[OX1])[#8X2][#7X2]=,:[#6X3;$([H0]([#6])[#6]),$([H1][#6]),$([H2])]'
33. 'Amidine: [NX3;!$(NC=[O,S])][CX3;$([CH]),$([C][#6])]=[NX2;!$(NC=[O,S])]'
34. 'Hydroxamic_acid_ester: [CX3;$([H0][#6]),$([H1])](=[OX1])[#7X3;$([H1]),$([H0][#6;!$(C=[O,N,S])])][OX2][#6;!$(C=[O,N,S])]'
35. 'Imidoylhalide_cyclic: [#6R][#6X3R](=,:[#7X2;$([H1]),$([H0][#6;!$(C=[O,N,S])])])[FX1,ClX1,BrX1,IX1]'
36. 'Alpha_aminoacid: [NX3,NX4+;!$([N]~[!#6]);!$([N]*~[#7,#8,#15,#16])][C][CX3](=[OX1])[OX2H,OX1-]'
37. 'Peptide_N_term: [NX3,NX4+;!$([N]~[!#6]);!$([N]*~[#7,#8,#15,#16])][C][CX3](=[OX1])[NX3;$([N][C][CX3](=[OX1])[NX3,OX2,OX1-])]'
38. 'Carbonic_acid_dieester: [#6;!$(C=[O,N,S])][#8X2][#6X3](=[OX1])[#8X2][#6;!$(C=[O,N,S])]'
39. 'Carbonic_acid_esterhalide: [#6;!$(C=[O,N,S])][OX2;!R][CX3](=[OX1])[OX2][FX1,ClX1,BrX1,IX1]'
40. 'Thiourea: [#7X3;!$([#7][!#6])][#6X3](=[SX1])[#7X3;!$([#7][!#6])]'
41. 'Urethan: [#7X3][#6](=[OX1])[#8X2][#6]'
42. 'Biuret: [#7X3][#6](=[OX1])[#7X3][#6](=[OX1])[#7X3]'
43. 'Thiocarbazide: [#7X3][#7X3][#6X3]([#7X3][#7X3])=[SX1]'
44. 'Thiocarbazone: [#7X2](=[#6])[#7X3][#6X3]([#7X3][#7X3])=[SX1]'
45. 'Oxoarene: [c]=[OX1]'
46. 'Heteroaromatic: [a;!c]'
47. 'Nitrite: [NX2](=[OX1])[O;$([X2]),$([X1-])]'
48. 'Azide: [NX1]~[NX2]~[NX2,NX1]'
49. 'Diazo: [$([#6]=[NX2+]=[NX1-]),$([#6-]-[NX2+]#[NX1])] '
50. 'Nitrosamide: [NX2](=[OX1])N-*=O'
51. 'N-Oxide: [$([#7+][OX1-]),$([#7v5]=[OX1]);!$([#7](~[O])~[O]);!$([#7]=[#7])] '
52. 'Hydrazine: [NX3;$([H2]),$([H1][#6]),$([H0]([#6])[#6]);!$(NC=[O,N,S])][NX3;$([H2]),$([H1][#6]),$([H0]([#6])[#6]);!$(NC=[O,N,S])]'
53. 'Hydroxylamine: [NX3;$([H2]),$([H1][#6]),$([H0]([#6])[#6]);!$(NC=[O,N,S])][OX2;$([H1]),$(O[#6;!$(C=[N,O,S])])]'
54. 'Sulfon: [$([SX4](=[OX1])(=[OX1])([#6])[#6]),$([SX4+2]([OX1-])([OX1-])([#6])[#6])]'
55. 'Sulfuric_monoamide: [SX4](=[OX1])(=[OX1])([#7X3;$([H2]),$([H1][#6;!$(C=[O,N,S])]),$([#7]([#6;!$(C=[O,N,S])])[#6;!$(C=[O,N,S])])])[$([OX2H]),$([OX1-])]'
56. 'Sulfuric_derivative: [SX4D4](=[!#6])(=[!#6])([!#6])[!#6]'
57. 'Sulfonic_ester: [SX4;$([H1]),$([H0][#6])](=[OX1])(=[OX1])[OX2][#6;!$(C=[O,N,S])]'
58. 'Sulfenic_amide: [SX2;$([H1]),$([H0][#6])][#7X3;$([H2]),$([H1][#6;!$(C=[O,N,S])]),$([#7]([#6;!$(C=[O,N,S])])[#6;!$(C=[O,N,S])])]'
59. 'Sulfenic_halide: [SX2;$([H1]),$([H0][#6])][FX1,ClX1,BrX1,IX1]'
60. 'Phosphonic_monoamide: [PX4;$([H1]),$([H0][#6])](=[OX1])([$([OX2H]),$([OX1-])])[#7X3;$([H2]),$([H1][#6;!$(C=[O,N,S])]),$([#7]([#6;!$(C=[O,N,S])])[#6;!$(C=[O,N,S])])]'
61. 'Phosphonic_diamide: [PX4;$([H1]),$([H0][#6])](=[OX1])([#7X3;$([H2]),$([H1][#6;!$(C=[O,N,S])]),$([#7]([#6;!$(C=[O,N,S])])[#6;!$(C=[O,N,S])])])[#7X3;$([H2]),$([H1][#6;!$(C=[O,N,S])]),$([#7]([#6;!$(C=[O,N,S])])[#6;!$(C=[O,N,S])])]'
62. 'Phosphonic_esteramide: [PX4;$([H1]),$([H0][#6])](=[OX1])([OX2][#6;!$(C=[O,N,S])])[#7X3;$([H2]),$([H1][#6;!$(C=[O,N,S])]),$([#7]([#6;!$(C=[O,N,S])])[#6;!$(C=[O,N,S])])]'
63. 'Phosphoric_acid: [PX4D4](=[OX1])([$([OX2H]),$([OX1-])])([$([OX2H]),$([OX1-])])[$([OX2H]),$([OX1-])]'
64. 'Phosphoric_monoester: [PX4D4](=[OX1])([$([OX2H]),$([OX1-])])([$([OX2H]),$([OX1-])])[OX2][#6;!$(C=[O,N,S])]'
65. 'Phosphoric_triamide: [PX4D4](=[OX1])([#7X3;$([H2]),$([H1][#6;!$(C=[O,N,S])]),$([#7]([#6;!$(C=[O,N,S])])[#6;!$(C=[O,N,S])])])([#7X3;$([H2]),$([H1][#6;!$(C=[O,N,S])]),$([#7]([#6;!$(C=[O,N,S])])[#6;!$(C=[O,N,S])])])[#7X3;$([H2]),$([H1][#6;!$(C=[O,N,S])]),$([#7]([#6;!$(C=[O,N,S])])[#6;!$(C=[O,N,S])])]'
66. 'Phosphoric_diestermonoamide: [PX4D4](=[OX1])([OX2][#6;!$(C=[O,N,S])])([OX2][#6;!$(C=[O,N,S])])[#7X3;$([H2]),$([H1][#6;!$(C=[O,N,S])]),$([#7]([#6;!$(C=[O,N,S])])[#6;!$(C=[O,N,S])])]'
67. 'Phosphinic_amide: [PX4;$([H2]),$([H1][#6]),$([H0]([#6])[#6])](=[OX1])[#7X3;$([H2]),$([H1][#6;!$(C=[O,N,S])]),$([#7]([#6;!$(C=[O,N,S])])[#6;!$(C=[O,N,S])])]'
68. 'Phosphinic_acid_derivative: [PX4;$([H2]),$([H1][#6]),$([H0]([#6])[#6])](=[!#6])[!#6]'
69. 'Phosphonous_monoester: [PX3;$([H1]),$([H0][#6])]([$([OX2H]),$([OX1-])])[OX2][#6;!$(C=[O,N,S])]'
70. 'Phosphinous_ester: [PX3;$([H2]),$([H1][#6]),$([H0]([#6])[#6])][OX2][#6;!$(C=[O,N,S])]'
71. 'Phosphinous_amide: [PX3;$([H2]),$([H1][#6]),$([H0]([#6])[#6])][#7X3;$([H2]),$([H1][#6;!$(C=[O,N,S])]),$([#7]([#6;!$(C=[O,N,S])])[#6;!$(C=[O,N,S])])]'
72. 'Quart_silane: [SiX4]([#6])([#6])([#6])[#6]'
73. 'Silylmonohalide: [SiX4]([FX1,ClX1,BrX1,IX1])([#6])([#6])[#6]'
74. 'Het_trialkylsilane: [SiX4]([!#6])([#6])([#6])[#6]'
75. 'Dihet_dialkylsilane: [SiX4]([!#6])([!#6])([#6])[#6]'
76. 'Trialkylborane: [BX3]([#6])([#6])[#6] '
77. 'Borohydride: [BH1,BH2,BH3,BH4]'
78. 'Sugar_pattern_1: [OX2;$([r5]1@C@C@C(O)@C1),$([r6]1@C@C@C(O)@C(O)@C1)]'
79. 'Sugar_pattern_2: [OX2;$([r5]1@C(!@[OX2,NX3,SX2,FX1,ClX1,BrX1,IX1])@C@C@C1),$([r6]1@C(!@[OX2,NX3,SX2,FX1,ClX1,BrX1,IX1])@C@C@C@C1)]'
80. 'Sugar_pattern_2_reducing: [OX2;$([r5]1@C(!@[OX2H1])@C@C@C1),$([r6]1@C(!@[OX2H1])@C@C@C@C1)]'
81. 'Conjugated_tripple_bond: *#*[*]=,#,:[*]'
82. 'Cis_double_bond: */[D2]=[D2]\*'
83. 'Trans_double_bond: */[D2]=[D2]/*'
84. 'Halogen_on_hetero: [FX1,ClX1,BrX1,IX1][!#6]'
85. 'C_ONS_bond: [#6]~[#7,#8,#16]'
86. 'Anion: [-1,-2,-3,-4,-5,-6,-7]'
87. 'Kation: [+1,+2,+3,+4,+5,+6,+7]'
88. 'Rotatable_bond: [!$(*#*)&!D1]-!@[!$(*#*)&!D1]'

RDKit MACCS Keys:

1. '1:(''?'',0), # ISOTOPE'
2. ' 5:(''[Sc,Ti,Y,Zr,Hf]'',0), # Group IIIB,IVB (Sc...) '
3. ' 7:(''[V,Cr,Mn,Nb,Mo,Tc,Ta,W,Re]'',0), # Group VB,VIB,VIIB'
4. ' 8:(''[!#6;!#1]1~*~*~*~1'',0), # QAAA@1'
5. ' 10:(''[Be,Mg,Ca,Sr,Ba,Ra]'',0), # Group IIa (Alkaline earth)'
6. ' 13:(''[#8]~[#7](~[#6])~[#6]'',0), # ON(C)C'
7. ' 20:(''[Si]'',0), #Si'
8. ' 29:(''[#15]'',0),# P'
9. ' 33:(''[#7]~[#16]'',0), # NS'
10. ' 34:(''[CH2]=*'',0), # CH2=A'
11. ' 36:(''[#16R]'',0), # S Heterocycle'
12. ' 38:(''[#7]~[#6](~[#6])~[#7]'',0), # NC(C)N'
13. ' 39:(''[#8]~[#16](~[#8])~[#8]'',0), # OS(O)O'
14. ' 40:(''[#16]-[#8]'',0), # S-O'
15. ' 44:(''?'',0), # OTHER'
16. ' 46:(''Br'',0), # BR'
17. ' 52:(''[#7]~[#7]'',0), # NN'
18. ' 55:(''[#8]~[#16]~[#8]'',0), #OSO'
19. ' 61:(''*~[#16](~*)~*'',0), # AS(A)A'
20. ' 63:(''[#7]=[#8]'',0), # N=O'
21. ' 68:(''[!#6;!#1;!H0]~[!#6;!#1;!H0]'',0), # QHQH (&...) SPEC Incomplete'
22. ' 72:(''[#8]~*~*~[#8]'',0), # OAAO'
23. ' 73:(''[#16]=*'',0), # S=A'
24. ' 80:(''[#7]~*~*~*~[#7]'',0), # NAAAN'
25. ' 83:(''[!#6;!#1]1~*~*~*~*~1'',0), # QAAAA@1'
26. ' 92:(''[#8]~[#6](~[#7])~[#6]'',0), # OC(N)C'
27. ' 93:(''[!#6;!#1]~[CH3]'',0), # QCH3'
28. ' 101:(''[$([R]@1@[R]@[R]@[R]@[R]@[R]@[R]@[R]1),$([R]@1@[R]@[R]@[R]@[R]@[R]@[R]@[R]@[R]1),$([R]@1@[R]@[R]@[R]@[R]@[R]@[R]@[R]@[R]@[R]1),$([R]@1@[R]@[R]@[R]@[R]@[R]@[R]@[R]@[R]@[R]@[R]1),$([R]@1@[R]@[R]@[R]@[R]@[R]@[R]@[R]@[R]@[R]@[R]@[R]1),$([R]@1@[R]@[R]@[R]@[R]@[R]@[R]@[R]@[R]@[R]@[R]@[R]@[R]1),$([R]@1@[R]@[R]@[R]@[R]@[R]@[R]@[R]@[R]@[R]@[R]@[R]@[R]@[R]1)]'',0), # 8M Ring or larger. This only handles up to ring sizes of 14'
29. ' 104:(''[!#6;!#1;!H0]~*~[CH2]~*'',0), # QHACH2A'
30. ' 107:(''[F,Cl,Br,I]~*(~*)~*'',0), # XA(A)A'
31. ' 109:(''*~[CH2]~[#8]'',0), # ACH2O'
32. ' 111:(''[#7]~*~[CH2]~*'',0), # NACH2A'
33. ' 112:(''*~*(~*)(~*)~*'',0), # AA(A)(A)A'
34. ' 119:(''[#7]=*'',0), # N=A'
35. ' 126:(''*!@[#8]!@*'',0), # A!O!A'
36. ' 134:(''[F,Cl,Br,I]'',0), # X (HALOGEN)'
37. ' 135:(''[#7]!:*:*'',0), # Nnot%A%A'
38. ' 140:(''[#8]'',3), # O > 3 (&...) Spec Incomplete'
39. ' 143:(''*@*!@[#8]'',0), # A$A!O'
40. ' 148:(''*~[!#6;!#1](~*)~*'',0), # AQ(A)A'
41. ' 149:(''[C;H3,H4]'',1), # CH3 > 1'
42. ' 150:(''*!@*@*!@*'',0), # A!A$A!A'
43. ' 152:(''[#8]~[#6](~[#6])~[#6]'',0), # OC(C)C'
44. ' 154:(''[#6]=[#8]'',0), # C=O'
45. ' 157:(''[#6]-[#8]'',0), # C-O'
46. ' 158:(''[#6]-[#7]'',0), # C-N'
47. ' 161:(''[#7]'',0), # N'
48. ' 162:(''a'',0), # Aromatic'
49. ' 163:(''*1~*~*~*~*~*~1'',0), # 6M Ring'
50. ' 164:(''[#8]'',0), # O'

Spectrophore features:

1. Spectrophore values calculated from the atomic partial charge (features: 1, 3, 5)
2. Spectrophore values calculated from the atomic lipophilicity properties (features: 13, 16, 19, 20, 23, 24)
3. Spectrophore values calculated from the atomic electrophilicity properties (feature 39)

Chemical descriptors:

1. MolLogP
2. Chi1
3. Chi4n
4. NumHDonors
5. NumValenceElectrons
6. SMR_VSA10
7. SlogP_VSA12
8. VSA_EState10

The remaining features are the ones based on the topological fingerprint of a molecule. The numbers of these features are hashed and cannot be easily decoded to bring their corresponding subgraph. Thus, the indices of the remaining features can be obtained by contacting the authors and better not to be listed here.
