# Supplementary material for: Mining Chemical Activity Status from High-Throughput Screening Assays
Source: PLoS One. 2015 Dec 14;10(12):e0144426. doi: 10.1371/journal.pone.0144426 (PMC4682830; doi:10.1371/journal.pone.0144426)
Supplement: S2 Text — (DOCX) [file pone.0144426.s005.docx]

# Mining chemical activity status in high-throughput screening assays

*Othman Soufan^1^, Wail Ba-alawi^1^, Moataz Afeef^1^, Magbubah Essack^1^****,*** *Valentin Rodionov^2^,* *Panos Kalnis^3^ and Vladimir B. Bajic^1,*^*

^1^King Abdullah University of Science and Technology (KAUST), Computational Bioscience Research Center (CBRC), Thuwal 23955-6900, Saudi Arabia. ^2^King Abdullah University of Science and Technology (KAUST), KAUST Catalysis Center (KCC), Thuwal 23955-6900, Saudi Arabia. ^3^King Abdullah University of Science and Technology (KAUST), Infocloud Group, Computer, Electrical and Mathematical Sciences and Engineering Division (CEMSE), Thuwal 23955-6900, Saudi Arabia.

# Supporting Information Text 2

**An analysis of the effect of feature selection results on classification performance.**

# Effect of Feature Selection on Prediction

DWFS tool for feature selection (FS) (1), selected a subset of 821 features out of 2,940 that were initially extracted based on chemical, topological and other 2D and 3D descriptors of chemicals to describe the compounds. We compared this feature subset with the standard 881 features from PubChem fingerprint (2). GSVM-RU is a data pre-processing method that removes through different iterations un-informative samples from the majority negative class. Since each round in GSVM-RU works with a different IR, we use it to compare the performance of the two different feature subsets (i.e. our proposed features and PubChem fingerprint features) under different levels of IR. This may help in drawing an insight about the more relevant features for the prediction system. In the study by Li et al.(3), a linear kernel SVM was used for finding the negative support vectors in GSVM-RU. Here, we additionally consider a polynomial kernel to compare the feature subsets. In Fig 1, a comparison between the proposed feature subset and the PubChem features using a GSVM-RU with a linear SVM kernel (GSVM-L) and degree 3 polynomial kernel (GSVM-P) is given. For the linear kernel (shown as filled circles), both feature subsets converge almost to the same GMean of sensitivity and specificity from early iteration steps starting from the 30^th^ iteration. GMean was used as part of the GSVM-RU algorithm.(3) Interestingly, for GSVM-P (shown as filled triangles), the performance is noticeably worse if the PubChem features are used, resulting in zero GMean of sensitivity and specificity for the initial 75 iterations. However, when features obtained by our feature selection method is used, a much higher GMean with maximum at about 85% was obtained with GSVM-P. Such dramatic difference is suspected to be the result of two factors: the nature of features and the set of selected features. All the features from PubChem are encoded as binary, which under certain conditions, as illustrated in Fig 1, may not be helpful for a classifier. Nevertheless, in our proposed subset of features, there is a set of 18 continuous features. From 881 features from PubChem, 238 features were only selected during the feature selection. In S1 Text, we provide more details about the generated features and the list of features we selected for the study.


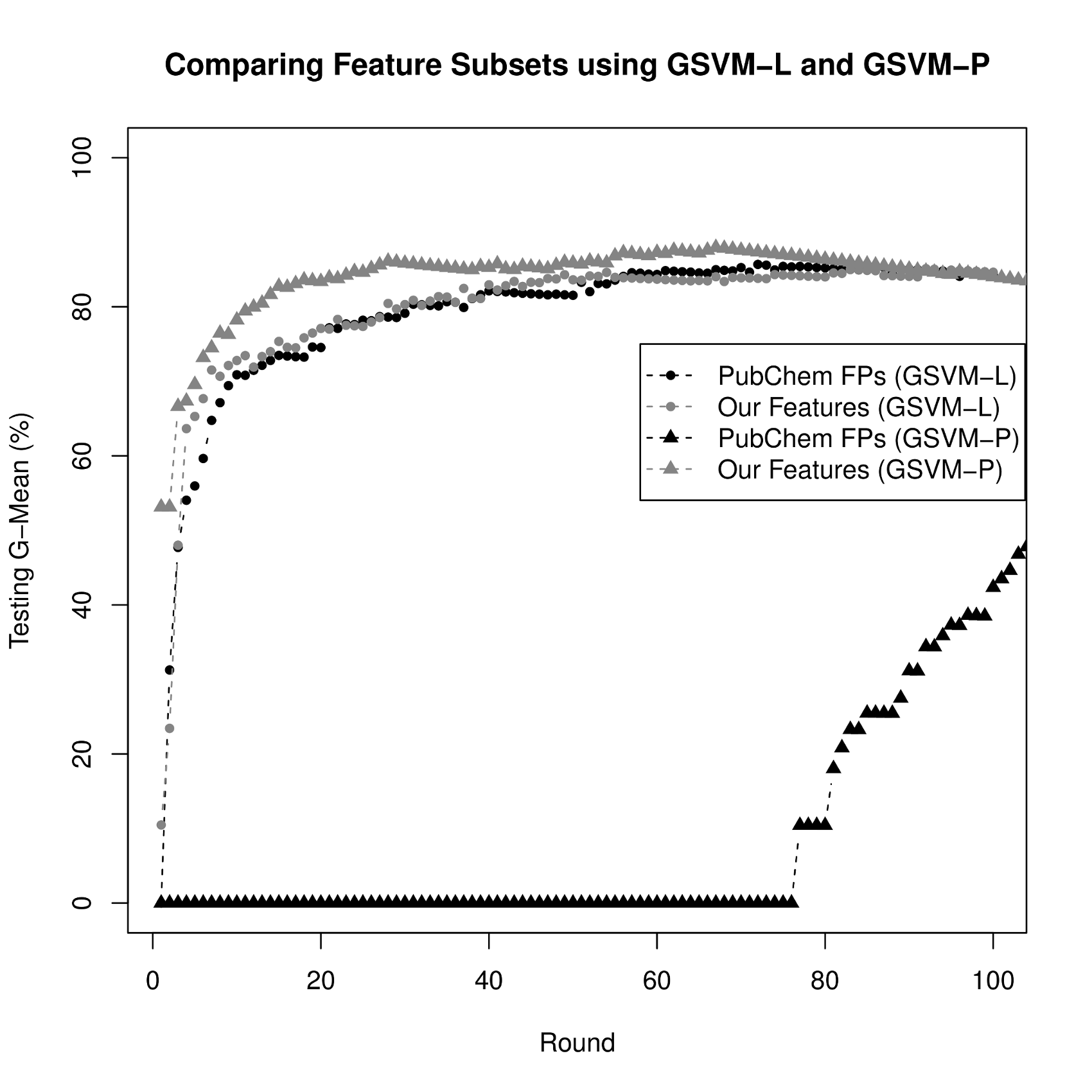


Fig 1: Comparing two different feature subsets using GSVM-RU over 20% testing data from BenchSet. We use BenchSet dataset since it has the largest majority class which allows us to evaluate performance of features over more iterations (100 steps in this case). Gray color represents the performance of our proposed feature subset. Black color is used for the standard 881 PubChem features. G-mean is used to evaluate performance of each feature subset for two reasons; 1) GSVM-RU used G-mean to optimize selection of data and 2) for this part of comparison it gives a sufficient indication of difference in performance under an imbalanced distribution.

1. Soufan O, Kleftogiannis D, Kalnis P, Bajic VB. DWFS: A Wrapper Feature Selection Tool Based on a Parallel Genetic Algorithm. PloS one. 2015;10(2):e0117988.

2. PubChem. PubChem Substructure Fingerprint 2009 [cited 2013 2/25/2013]. Available from: <ftp://ftp.ncbi.nlm.nih.gov/pubchem/specifications/pubchem_fingerprints.txt>.

3. Li Q, Wang Y, Bryant SH. A novel method for mining highly imbalanced high-throughput screening data in PubChem. Bioinformatics. 2009;25(24):3310-6.
