# Supplementary material for: Mining Chemical Activity Status from High-Throughput Screening Assays
Source: PLoS One. 2015 Dec 14;10(12):e0144426. doi: 10.1371/journal.pone.0144426 (PMC4682830; doi:10.1371/journal.pone.0144426)
Supplement: S3 Text — The file includes also all information about DRAMOTE and its procedure. (DOCX) [file pone.0144426.s006.docx]

# **Mining chemical activity status in high-throughput screening assays**

*Othman Soufan^1^, Wail Ba-alawi^1^, Moataz Afeef^1^, Magbubah Essack^1^****,*** *Valentin Rodionov^2^,**Panos Kalnis^3^ and Vladimir B. Bajic^1,*^*

^1^King Abdullah University of Science and Technology (KAUST), Computational Bioscience Research Center (CBRC), Thuwal 23955-6900, Saudi Arabia. ^2^King Abdullah University of Science and Technology (KAUST), KAUST Catalysis Center (KCC), Thuwal 23955-6900, Saudi Arabia. ^3^King Abdullah University of Science and Technology (KAUST), Infocloud Group, Computer, Electrical and Mathematical Sciences and Engineering Division (CEMSE), Thuwal 23955-6900, Saudi Arabia.

# **Supporting Information Text 3**

### **Details about the existing state-of-the-art solutions used in the study and their input parameters. The file includes also all information about DRAMOTE and its procedure.**

### **Existing methods**

RU randomly selects a subset of the majority class instances and removes them in order to achieve better class balance. In our experiments, we apply RU so that both of the classes have exactly the same number of instances. This solution, in addition for being fast and straightforward, holds the advantage of reducing the size of the data set and this leads to faster classifier training. SMOTE is another data-based method based on the idea of generating random points on the line between two nearest neighbor points (i.e. interpolation) in the minority class (1). This, in turn, fills the gaps in the minority class and is expected to enhance the generalization ability of the classifier. SMOTE with equal setting for perfect balance between the highly imbalanced classes in a dataset would enlarge the size of minority to match the majority and thus, almost double the size of the dataset. To avoid increasing size of a dataset this much, we synthetically generate 200% of the minority class as advised in (2) for all synthetic based approaches including SMOTE, MWMOTE and DRAMOTE. A different dynamic method based on the output of a classifier that removes instances and balances the classes is GSVM-RU. Through generating sets of negative and positive support vectors called granules, GSVM-RU removes unnecessary negative instances and iteratively enhances classification. It is based on the assumption that under extreme class imbalance, the ideal linear classifier should be pushed towards the negative class (3, 4). Selective under-sampling is applied by removing only the negative support vectors. Thus, the IR is enhanced slightly and the process is repeated several times. This method was applied previously to BenchSet and results were reported in (5). Another very recent method that addresses the need to generate informative samples since existing over-sampling methods may generate the wrong synthetic minority samples in some scenarios is MWMOTE (2). This method, carefully analyzes the region of minority samples including the nearest-neighbor decision boundary and assigns a weight of importance for the minority samples. These weights are, in turn, used to guide the generation of new informative samples. Supporting Information S2 provides details about the selected percentages of RU and over-sampling for each dataset.

### **The Active Learning Approach**

Active Learning (AL) is based on the idea of allowing the classifier to choose its training data ^(6)^. AL is an attractive approach when labeling samples is costly, and, thus, there is a preference to select only samples that the model needs to know about. The approach, generally, is based on some query strategy framework and a selection criterion to sample the most relevant instance from a pool of input samples ^(6)^. The concept of AL can be easily extended as a solution to the class imbalance problem but, unfortunately, a small number of studies have addressed this ^(7),(8, 9)^. In this work, we used ideas from AL to query importance of training samples based on their contribution to number of false positive predictions of a particular classifier and then, apply interpolation for oversampling based on this feedback information.

### **DRAMOTE: our proposed solution**

Our variant of minority oversampling techniques is motivated by ideas from active learning (AL) . The method is based on establishing a feedback loop with the classifier to highlight points contributing most to its precision (other performance metrics can be used). DRAMOTE starts by:

1/ Training a classifier on a sample set of points from the training data and examine the prediction scores as well as the prediction labels produced by the classifier.

2/ Then, DRAMOTE assigns weights to the training minority class samples according to the overall precision performance of a classifier (i.e. global view; Equation (9)) and its performance in the surrounding region of these minority samples (i.e. local view; of Equation (8)). In Equation (8), is an indicator function that assigns +1 if sample is correctly classified and -1 otherwise. accounts for the classifier behavior in the surrounding region of sample and it is based on exploiting the probability output scores for sample and its $k$neighbors. Overall, Equation (8) assigns higher weights for those misclassified samples that contribute more towards precision.

3/ Step 2 is repeated for several time steps $t$ such that in every step a different sample for training is generated to draw a better picture of how the minority samples are contributing to precision under different circumstances.

4/ After learning weights (averaged over the $t$steps) of the minority class samples indicating how informative they are for precision estimation, we build a random sampling distribution using these weights for choosing the minority samples. In other words, samples affecting the precision highly are sampled more frequently for synthetically generating new effective samples.

$w_{it}=e^{(-I_{t}(x_{i})\times p_{t}(x_{i},k)\times\alpha_{t})}$ (1)

$\alpha_{t}=\frac{1}{2}\times ln(\frac{{precision}_{t}}{2-{precision}_{t}})$ (2)

5/ In the fifth phase, DRAMOTE generates the synthetic samples based on interpolation between the sampled minority points from step 2 and another informative nearest neighbor. DRAMOTE, rather than interpolating with a random nearest neighbor as done by other methods like SMOTE, synthetically generates the samples in a direction that shall not cause an overlap with the majority samples. This direction is chosen based on selecting a nearest neighbor point assigned a lower weight by Equation (8). This point that we call precision safe nearest neighbor in Equation (10), will give support for while not harming the classifier performance in the surrounding region of .

$synthetic\left( x_{i} \right)=x_{i}+rand(0..1)\times(precSafeNN\left( x_{i} \right)-x_{i})$ (3)

Algorithm DRAMOTE+( D, L, C, N, k, r )

Input: Training data D; Training Labels L; Classifier C; Amount of synthetic samples N%; Number of nearest neighbors k; Number of nearest neighbors for evaluating performance in a surrounding region r

Output: (N/100) * (T) synthetic minority class samples; T is the number of minority class samples

weights[]: array for importance weights of minority samples

SMOTE(D, L, N, k)

//Evaluate weights for every sample

1. for $f\leftarrow3:6$

2. //Evaluate performance for each sample under different conditions by means of

3. //different cross-validation splits of the training data.

4. c = cross-validation-partition(size(data, 1), ‘kfold’, $f$);

5. for $i\leftarrow1:f$

6. [predicted-probabilities, predicted-Labels] = C(D, L, c, $i$);//Train over i-th fold 7. in this c-partition

8. //weightupdate uses equations (1) & (2) in manuscript

9. weights[$i$-set] = weights[$i$-set] + weightupdate(D, L, predicted-probabilities, predicted-Labels, r);

10. endfor

11. endfor

12. //Normalize weights

13. weights[] = weights[]./$f$;

14. //Build a random distribution based on weights

15. R = randsample(1:T, N, true, weights);

16. //Synthetically generate new samples based on weight of importance and direction

17. Smin[]; //synthetic minority samples set

18. for $i\leftarrow1:N$

19. x = pos(R(i), :); //pos is the positive samples in D

20. [IDX] = knnsearch(pos, x, 'k', k);

21. nn_weights = weights(IDX);

22. [nn_idx] = min(nn_weights);

23. y = pos(IDX(nn_i), :); //choose a sample that is not in a dangerous zone to interpolate with

24. s = x + rand(1)*(y-x);

25. Smin = [Smin; s];

26. endfor

|  |  | (4) |
| --- | --- | --- |

In Equation (1), is a score at current step $t$ indicating to which extent the surrounding $k$points are affecting minority sample . In the previous steps, the Euclidian distance served as the basis for querying the "surrounding" points or the nearest neighbors. Nevertheless, two points that are close in the Euclidean space might not be close in the mapping space of a classifier. For example, an SVM-classifier with an RBF kernel can shrink the distances between samples and change the "surrounding" information and in such case, the Euclidean distance is not representative. So, there is a dilemma of whether the "surrounding" points in the Euclidean space are the same as "surrounding" points a classifier looks at. The cause of this problem is that we assume we do not know the actual shape of a decision boundary. We can, however, account implicitly for this by considering the probabilities of the classifier to assign these points to the same class. In simpler words, the difference between classifier’s output probabilities assigned for a sample and its nearest neighbor, helps in indicating how closely they are with regard to the decision boundary. For example, two points that are close in the Euclidean space but has very different output probabilities are probably not close to each other with regard to the actual decision boundary. The intuition is that if a sample and its nearest neighbor from the training set, have high probability values highlighting that the classifier is really confident they are of different classes, then, less appreciation is given for the Euclidean distance in this case (i.e. even if they were really close but the decision boundary is saying no by means of probability scores, we should ignore them as being neighbors). Equation (1) reflects this by multiplying by Equation (1), overall, ranks points and assigns them weights of importance as follows:

1. Positive (or minority) samples that are misclassified as negatives (i.e. false positives) are ranked highest.
2. Minority samples that are correctly classified but their local surrounding region is a region where the classifier is not performing very well, have higher ranking than other correctly classified region.
3. Minority samples that are misclassified but at a stage where the classifier is performing very well in terms of precision, will maintain lower ranking of importance to the case when the classifier is performing poor in terms of precision.

### **Methods’ parameters selection**

It should be noted that for any particular selection of parameters, all preprocessing methods use the same setup wherever possible to avoid any potential bias. In addition, selection of RU and SMOTE percentages was **not** based on fine tuning any single method. We synthetically generate 200% of the minority class as advised in (2). If the performance for all synthetic based method was very poor, then another percentage was examined different from 200%. Usually, 400%.

If we had a large datasets with more than 10,000 samples, we randomly under sample the majority samples in the training folds to 10,000 and use all the available majority points, otherwise. Based on initial experiments with 10,000 reflected good performance levels and helped in speeding up the extensive comparison we had. Only for AID 938, we used the full dataset to be able to get better indications as AID 938 was our case study.

Overall, the selection of these parameters is very difficult especially when dealing with larger datasets. Thus, in this study we resort to a previously recommended setup, for example as in (2) to serve as the default setup. Only in few cases, we examine different parameters, when performance scores were very poor.

Details about selection of parameters can be found in the following table for each dataset:

| Dataset | SMOTE% | RU Size | Notes |
| --- | --- | --- | --- |
| BenchSet | 400 | 10,000 | 200% was leading to low performance for all synthetic based method and thus, 400% was used. |
| AID 596 | 200 | 10,000 |  |
| AID 618 | 400 | 10,000 | 200% was leading to low performance for all synthetic based method and thus, 400% was used. |
| AID 644 | 200 | --- |  |
| AID 886 | 200 | 10,000 |  |
| AID 899 | 200 | --- |  |
| AID 938 | 50 | --- | Increasing 200% to 400% did not improve performance of all examined synthetic based methods. Thus, we used 50% as one of the choices recommended in (1). |
| AID 743042 | 200 | --- |  |
| AID 743288 | 200 | --- |  |

References

1. Chawla NV, Bowyer KW, Hall LO, Kegelmeyer WP. SMOTE: synthetic minority over-sampling technique. arXiv preprint arXiv:11061813. 2011.

2. Barua S, Islam M, Yao X, Murase K. MWMOTE--Majority Weighted Minority Oversampling Technique for Imbalanced Data Set Learning. Knowledge and Data Engineering, IEEE Transactions on. 2014;26(2):405-25.

3. Tang Y, Zhang Y-Q, Chawla NV, Krasser S. SVMs modeling for highly imbalanced classification. Systems, Man, and Cybernetics, Part B: Cybernetics, IEEE Transactions on. 2009;39(1):281-8.

4. Tang Y, Zhang Y-Q, editors. Granular SVM with repetitive undersampling for highly imbalanced protein homology prediction. Granular Computing, 2006 IEEE International Conference on; 2006: IEEE.

5. Li Q, Wang Y, Bryant SH. A novel method for mining highly imbalanced high-throughput screening data in PubChem. Bioinformatics. 2009;25(24):3310-6.

6. Settles B. Active learning literature survey. University of Wisconsin, Madison. 2010.

7. Ertekin S, Huang J, Bottou L, Giles L, editors. Learning on the border: active learning in imbalanced data classification. Proceedings of the sixteenth ACM conference on Conference on information and knowledge management; 2007: ACM.

8. Li S, Ju S, Zhou G, Li X, editors. Active learning for imbalanced sentiment classification. Proceedings of the 2012 Joint Conference on Empirical Methods in Natural Language Processing and Computational Natural Language Learning; 2012: Association for Computational Linguistics.

9. Tomanek K, Hahn U, editors. Reducing class imbalance during active learning for named entity annotation. Proceedings of the fifth international conference on Knowledge capture; 2009: ACM.
