# Supplementary material for: Mining Chemical Activity Status from High-Throughput Screening Assays
Source: PLoS One. 2015 Dec 14;10(12):e0144426. doi: 10.1371/journal.pone.0144426 (PMC4682830; doi:10.1371/journal.pone.0144426)
Supplement: S4 Text — (DOCX) [file pone.0144426.s007.docx]

# **Mining chemical activity status in high-throughput screening assays**

*Othman Soufan^1^, Wail Ba-alawi^1^, Moataz Afeef^1^, Magbubah Essack^1^****,*** *Valentin Rodionov^2^,* *Panos Kalnis^3^ and Vladimir B. Bajic^1,*^*

^1^King Abdullah University of Science and Technology (KAUST), Computational Bioscience Research Center (CBRC), Thuwal 23955-6900, Saudi Arabia. ^2^King Abdullah University of Science and Technology (KAUST), KAUST Catalysis Center (KCC), Thuwal 23955-6900, Saudi Arabia. ^3^King Abdullah University of Science and Technology (KAUST), Infocloud Group, Computer, Electrical and Mathematical Sciences and Engineering Division (CEMSE), Thuwal 23955-6900, Saudi Arabia.

# **Supporting Information Text 4**

**Detailed docking scores including the set of random selected drugs and description of the docking procedure**

For setting up the docking simulations, we need to specify the grid parameters. We use CASTp [1] over the TSHR protein to identify a potential set of activation sites in order to center the grid. Having determined the grid parameters, we follow a docking setup similar to the one in [2] where two sets of docking experiments including actual drug (acting as a reference for evaluation) and proposed drug sets are carried over. AutoDock Vina [3] is then used to dock our top 10 predictions over AID 938 dataset and to dock the experimental actual top 10 interactions reported in PubChem database for AID 938. In addition, docking is carried over 10 randomly selected drugs from the approved list of DrugBank [4]. Providing docking results for these three sets gives a better clue of how good are our proposed set based on referencing it to the other two sets. The random seed of AutoDock Vina was fixed for all docking experiments and for the other parameters, we use the default ones. Finally, we report the best docking results in terms of free binding energy and RMSD values of each docked drug.

The following table provides the individual binding free energy as well as RMSD scores for all top ranked predictions using DRAMOTE ensemble of six classifiers.

| **Actual Top Interactions from PubChem** | | | **Predicted Top Interactions using DRAMOTE** | | | **RANDOM Selected Drugs** | | |
| --- | --- | --- | --- | --- | --- | --- | --- | --- |
| **PubChem ID (activity score)** | **Binding Energy (kcal/mol)** | **RMSD** | **DrugBank ID** | **Binding Energy (kcal/mol)** | **RMSD** | **DrugBank ID** | **Binding Energy (kcal/mol)** | **RMSD** |
| CID 3246906 (99) | -5.1 | 2.125 | DB00405 | -5.8 | 1.118 | DB00132 | -3 | 6.891 |
| CID 6604888 (94) | -6.1 | 1.976 | DB00748 | -5.2 | 1.887 | DB00185 | -5.4 | 2.023 |
| CID 2868392 (93) | -7 | 1.776 | DB00889 | -6.9 | 2.361 | DB00307 | -6.5 | 3.803 |
| CID 6604115 (92) | -6.1 | 1.325 | DB00904 | -7.1 | 2.034 | DB00496 | -9.1 | 3.742 |
| CID 6604280 (99) | -5.2 | 1.648 | DB00962 | -6.3 | 2.144 | DB00561 | -7.7 | 2.385 |
| CID 5280360 (99) | -5 | 1.915 | DB01261 | -7 | 2.389 | DB00650 | -7.2 | 1.693 |
| CID 3034756 (98) | -5.4 | 2.33 | DB01342 | -6.3 | 1.099 | DB01014 | -6.5 | 3.075 |
| CID 443372 (92) | -4.8 | 1.167 | DB01349 | -9.1 | 1.781 | DB01133 | -6.5 | 4.846 |
| CID 6604815 (94) | -9.1 | 1.825 | DB06267 | -7.1 | 1.905 | DB01194 | -6.1 | 1.573 |
| CID 6604901 (92) | -5.9 | 2.12 | DB06439 | -9.9 | 1.972 | DB01421 | -7.7 | 4.421 |
| Average | **-5.97** | **1.8207** | Average | **-7.07** | **1.869** | Average | -6.57 | 3.4452 |

References

1. Dundas J, Ouyang Z, Tseng J, Binkowski A, Turpaz Y, Liang J: **CASTp: computed atlas of surface topography of proteins with structural and topographical mapping of functionally annotated residues**. *Nucleic acids research* 2006, **34**(suppl 2):W116-W118.

2. Engin HB, Keskin O, Nussinov R, Gursoy A: **A strategy based on protein–protein interface motifs may help in identifying drug off-targets**. *Journal of chemical information and modeling* 2012, **52**(8):2273-2286.

3. Trott O, Olson AJ: **AutoDock Vina: improving the speed and accuracy of docking with a new scoring function, efficient optimization, and multithreading**. *Journal of computational chemistry* 2010, **31**(2):455-461.

4. Wishart DS, Knox C, Guo AC, Shrivastava S, Hassanali M, Stothard P, Chang Z, Woolsey J: **DrugBank: a comprehensive resource for in silico drug discovery and exploration**. *Nucleic acids research* 2006, **34**(suppl 1):D668-D672.
