# Supplementary material for: Mining Chemical Activity Status from High-Throughput Screening Assays
Source: PLoS One. 2015 Dec 14;10(12):e0144426. doi: 10.1371/journal.pone.0144426 (PMC4682830; doi:10.1371/journal.pone.0144426)
Supplement: S5 Text — (DOCX) [file pone.0144426.s008.docx]

# **Mining chemical activity status in high-throughput screening assays**

*Othman Soufan^1^, Wail Ba-alawi^1^, Moataz Afeef^1^, Magbubah Essack^1^****,*** *Valentin Rodionov^2^,* *Panos Kalnis^3^ and Vladimir B. Bajic^1,*^*

^1^King Abdullah University of Science and Technology (KAUST), Computational Bioscience Research Center (CBRC), Thuwal 23955-6900, Saudi Arabia. ^2^King Abdullah University of Science and Technology (KAUST), KAUST Catalysis Center (KCC), Thuwal 23955-6900, Saudi Arabia. ^3^King Abdullah University of Science and Technology (KAUST), Infocloud Group, Computer, Electrical and Mathematical Sciences and Engineering Division (CEMSE), Thuwal 23955-6900, Saudi Arabia.

# **Supporting Information Text 5**

**Extended literature review of the top predicted FDA drugs for the TSHR in humans**

A literature review of our top predictions points out that **Ondansetron** (our top ranked prediction) **and Granisetron** (seventh ranked prediction) are serotonin 5-HT_3_ receptor antagonist used to prevent nausea caused by chemotherapy, radiation therapy and surgery (1-3). Drugs similar to these drugs include dolasetron (Anzemet), or palonosetron (Aloxi). Ondansetron has been shown to affect both peripheral and central nerves by: 1/ Ondansetron reducing the activity of the vagus nerves that activates the vomiting center in the medulla oblongata (4), and 2/ blocking serotonin receptors in the chemoreceptor trigger zone (5). It is not administered to patients for vomiting caused by motion sickness (6) and it has been demonstrated that this drug has no effect on dopamine receptors and muscarinic receptors (7, 8). However, its effect on TSH receptors has not been investigated. Nonetheless, eHealthMe published a review of side effects of 2’835 patients taking Ondansetron collated from the FDA and social media (9). In eight of these patients increased blood thyroid stimulating hormone was detected (9). Moreover, studies have demonstrated that a relationship between thyroid hormone and the brain serotonin (5-HT) system exists, that is, hypothyroid patients have shown a reduced 5-HT responsiveness that is reversible with thyroid replacement therapy (10). However, more studies are needed to clarify the mechanism that modulated this relation.

**Zaleplon,** our second ranked prediction, is marketed under brand names such as Sonata, Starnoc and Andante. It is a nonbenzodiazepine sedative-hypnotic drug used for the treatment of insomnia (11, 12). This nonbenzodiazepine has a similar chemical structure to the benzodiazepines. Drugs similar to Zaleplon include Zolpidem, Zopiclone and Eszopiclone all of which induce hypnotic effects as specific agonists at the benzodiazepine GABA_A_ α_1_ sub-receptor sites (13, 14). Zaleplon also binds with lower affinity to the GABA_A_ subsites α_2_ and α_3_ inducing anxiolytic and muscle relaxant effects and to the α_5_ subsite inducing negligible anticonvulsant effects (15, 16). A link between sleep and thyroid function has been established in rat based laboratory experiments (17, 18), nonetheless Zoleplon’s effect on the TSH receptors have not been studied. Sleep deprivation in these rats showed signs similar to hyperthyroidism and reduced circulating concentrations of total and free triiodothyronine (T_3_) and thyroxine (T_4_) without an increase in TSH (17, 19). While acute sleep loss in humans was reported to be associated with increased T_3_, thyroxine T_4_, and TSH (20, 21), contradictory to normal human sleep patterns that show acute inhibition of TSH secretion overnight (20).

**Dexbrompheniramine** (fourth ranked prediction) is usually used as a combination medicine with pseudoephedrine to treat symptoms of allergies and the common cold (22, 23). While **Tyloxapol** (sixth ranked prediction) is a polymer of the alkyl aryl polyether alcohol type, used as a surfactant to aid liquefaction and removal of mucus and pus from bronchopulmonary secretions (24, 25). **Carbinoxamine** (ninth ranked prediction), is a antihistamine and anticholinergic agent used to treat allergic rhinitis, allergic conjunctivitis, vasomotor rhinitis, dermatographism, amelioration (severe allergic reaction to blood plasma), anaphylactic reactions (used in conjunction with epinephrine), and allergic skin reactions due to angioedema or urticarial (26). It is a histamine antagonist, specifically acting as an H1-antagonist that is combined with pseudoephedrine, paracetamol, aspirin, and codeine. Literature does not provide any evidence of a link between Dexbrompheniramine or Tyloxapol or Carbinoxamine with TSHR or significant changes in the thyroid stimulating hormone concentrations in patients treated with these drugs.

**Sitagliptin** (fifth ranked prediction) is an antidiabetic drug of the dipeptidyl peptidase-4 (DPP-4) inhibitor class. It is used alone or in combination with antihyperglycemic agents such as metformin or thiazolidinedione for treatment of diabetes mellitus type 2 (27). On September 25, 2009, Sitagliptin marketed under the names Januvia or Janumet, revised there warning label as advised by the FDA to include, ‘Januvia or Janumet--a combination drug comprised of Januvia and Metformin--regarding Januvia side effects, it now includes Januvia pancreatic cancer and Januvia thyroid cancer.’ (28)

**Udenafil** (tenth ranked prediction) is proposed to be a phosphodiesterase type-5 (PDE 5) inhibitor (29) used to treat erectile dysfunction (ED) (30)**.** PDE5 is well-recognized as a regulator for smooth muscle contraction in penis and lung (31). There are approximately 11 families of PDE that catalyze the hydrolysis of *cAMP* and *cGMP* (29, 32, 33). Since the assay (an assay that detects agonist of the TSHR) used in this study is a cell-based assay that uses the premise that stimulation of *cAMP* production causes the CNG to open and subsequent membrane depolarization to occur. Udenafils’ activity as a PDE5 inhibitor may be partly indirect. However, Udenafil binding to the TSHR has not been experimentally validated as yet. Moreover, Slag and colleagues (34) reported that 10% of 401 men with ED had primary hypogonadism, 9% had secondary hypogonadism, 5% had hypothyroidism, 1% had hyperthyroidism and 4% had hyperprolactinemia.

In summary, we present in this study a novel approach to effectively screen *in silico* thousands of chemical compounds in a HTS setup. However, unlike many machine learning approaches, the DRAMOTE method establishes a feedback loop with the underlying model allowing for selection of more informative training samples. Importantly, DRAMOTE enhances precision for screening compounds while preserving sensitivity levels comparable to other existing methods. Our case study also provides a plausible list of TSHR agonists. However, if we consider Sitagliptin inducing thyroid cancer as a side-effect and Tasosartan being withdrawn from trials for inducing liver toxicity, the top ranked predictions should not only be considered for drug repositioning but also as warnings of drug-targets interactions that may be producing undesirable side-effects for existing drugs.

References

1. Billio A, Morello E, Clarke MJ. Serotonin receptor antagonists for highly emetogenic chemotherapy in adults. The Cochrane Library. 2010.

2. FDA Approves Sancuso: Drugs.com; 2008 [cited 2015 2/11/2015]. Available from: <http://www.drugs.com/newdrugs/fda-approves-sancuso-first-only-patch-preventing-nausea-vomiting-cancer-patients-undergoing-1119.html>.

3. Evans MC, Kaplan S. One Year Post-Pediatric Exclusivity Post-marketing Adverse Event Review: Drug Use Data Zofran (ondansetron HCl), Memorandum. In: Services DoHaH, editor.: U.S. Food and Drug Administration; 2006.

4. Gan TJ. Selective serotonin 5-HT3 receptor antagonists for postoperative nausea and vomiting. CNS drugs. 2005;19(3):225-38.

5. Kamm M. The complexity of drug development for irritable bowel syndrome. Alimentary pharmacology & therapeutics. 2002;16(3):343-51.

6. Muth E, Elkins A. 27 High dose ondansetron is not effective for reducing motion sickness in highly susceptible subjects. Neurogastroenterology & Motility. 2006;18(6):488-.

7. Wishart DS, Tzur D, Knox C, Eisner R, Guo AC, Young N, et al. HMDB: the human metabolome database. Nucleic acids research. 2007;35(suppl 1):D521-D6.

8. Tramèr MR, Reynolds D, Moore RA, McQuay HJ. Efficacy, dose-response, and safety of ondansetron in prevention of postoperative nausea and vomiting: a quantitative systematic review of randomized placebo-controlled trials. Anesthesiology. 1997;87(6):1277-89.

9. Review: could Ondansetron cause Blood thyroid stimulating hormone increased? ehealthme.com: eHealthMe; 2010 [cited 2015 2/11/2015]. Available from: <http://www.ehealthme.com/ds/ondansetron/blood+thyroid+stimulating+hormone+increased>.

10. Bauer M, Heinz A, Whybrow P. Thyroid hormones, serotonin and mood: of synergy and significance in the adult brain. Molecular psychiatry. 2001;7(2):140-56.

11. Huedo-Medina TB, Kirsch I, Middlemass J, Klonizakis M, Siriwardena AN. Effectiveness of non-benzodiazepine hypnotics in treatment of adult insomnia: meta-analysis of data submitted to the Food and Drug Administration. BMJ: British Medical Journal. 2012;345.

12. Elie R, Rüther E, Farr I, Emilien G, Salinas E. Sleep latency is shortened during 4 weeks of treatment with zaleplon, a novel nonbenzodiazepine hypnotic. Zaleplon Clinical Study Group. The Journal of clinical psychiatry. 1999;60(8):536-44.

13. Atack J. Development of Subtype-Selective GABAA Receptor Compounds for the Treatment of Anxiety, Sleep Disorders and Epilepsy. In: Monti JM, Pandi-Perumal SR, Möhler H, editors. GABA and Sleep: Springer Basel; 2010. p. 25-72.

14. McKernan R, Rosahl T, Reynolds D, Sur C, Wafford K, Atack J, et al. Sedative but not anxiolytic properties of benzodiazepines are mediated by the GABAA receptor α1 subtype. Nature neuroscience. 2000;3(6):587-92.

15. Rudolph U, Möhler H. Analysis of GABAA receptor function and dissection of the pharmacology of benzodiazepines and general anesthetics through mouse genetics. Annu Rev Pharmacol Toxicol. 2004;44:475-98.

16. Nutt DJ, Stahl SM. Searching for perfect sleep: the continuing evolution of GABAA receptor modulators as hypnotics. Journal of Psychopharmacology. 2010;24(11):1601-12.

17. Everson CA, Reed H. Pituitary and peripheral thyroid hormone responses to thyrotropin-releasing hormone during sustained sleep deprivation in freely moving rats. Endocrinology. 1995;136(4):1426-34.

18. BALZANO S, BERGMANN BM, GILLILAND MA, SILVA JE, RECHTSCHAFFEN A, REFETOFF S. Effect of Total Sleep Deprivation on 5′-Deiodinase Activity of Rat Brown Adipose Tissue*. Endocrinology. 1990;127(2):882-90.

19. Everson CA, Nowak TS. Hypothalamic thyrotropin-releasing hormone mRNA responses to hypothyroxinemia induced by sleep deprivation. American Journal of Physiology-Endocrinology and Metabolism. 2002;283(1):E85-E93.

20. Kuhs H, Färber D, Tölle R. Serum prolactin, growth hormone, total corticoids, thyroid hormones and thyrotropine during serial therapeutic sleep deprivation. Biological psychiatry. 1996;39(10):857-64.

21. Brabant G, Prank K, Ranft U, Schuermeyer T, Wagner T, Hauser H, et al. Physiological Regulation of Circadian and Pulsatile Thyrotropin Secretion in Normal Man and Woman*. The Journal of Clinical Endocrinology & Metabolism. 1990;70(2):403-9.

22. Löfkvist T, Svensson G. A comparative evaluation of oral decongestants in the treatment of vasomotor rhinitis. Journal of International Medical Research. 1978;6(1):56-60.

23. Payne JW. Drixoral: Why the Allergy Medicine Isn't Available, and What to Use Instead. U.S. News and World Report; 2009.

24. Rubin BK, Ramirez O, King M. Mucus rheology and transport in neonatal respiratory distress syndrome and the effect of surfactant therapy. CHEST Journal. 1992;101(4):1080-5.

25. Sehgal S, Ewing C, Richards T, Taeusch H. Modified bovine surfactant (Survanta) versus a protein-free surfactant (Exosurf) in the treatment of respiratory distress syndrome in preterm infants: a pilot study. Journal of the National Medical Association. 1994;86(1):46.

26. FDA Approves New Drug Application for Carbinoxamine: lgmpharma.com; 2013 [cited 2015 2/11/2015]. Available from: <http://www.lgmpharma.com/blog/fda-approves-new-drug-application-for-carbinoxamine/>.

27. Sitagliptin for Type 2 Diabetes. Available from: <http://www.nps.org.au/consumers/publications/medicine_update/issues/sitagliptin>.

28. Januvia Pancreatic Cancer, Januvia Thyroid Cancer.

29. Sandner P, Hütter J, Tinel H, Ziegelbauer K, Bischoff E. PDE5 inhibitors beyond erectile dysfunction. International journal of impotence research. 2007;19(6):533-43.

30. Kang SG, Kim JJ. Udenafil: efficacy and tolerability in the management of erectile dysfunction. Therapeutic advances in urology. 2012:1756287212470019.

31. Bender AT, Beavo JA. Cyclic nucleotide phosphodiesterases: molecular regulation to clinical use. Pharmacological reviews. 2006;58(3):488-520.

32. Glossmann H, Petrischor G, Bartsch G. Molecular mechanisms of the effects of sildenafil (VIAGRA< sup>®</sup>). Experimental gerontology. 1999;34(3):305-18.

33. Corbin JD, Francis SH. Cyclic GMP phosphodiesterase-5: target of sildenafil. Journal of Biological Chemistry. 1999;274(20):13729-32.

34. Slag MF, Morley JE, Elson MK, Trence DL, Nelson CJ, Nelson AE, et al. Impotence in medical clinic outpatients. Jama. 1983;249(13):1736-40.

35. Marchais-Oberwinkler S, Henn C, Möller G, Klein T, Negri M, Oster A, et al. 17β-Hydroxysteroid dehydrogenases (17β-HSDs) as therapeutic targets: protein structures, functions, and recent progress in inhibitor development. The Journal of steroid biochemistry and molecular biology. 2011;125(1):66-82.
