# Supplementary material for: Mining Chemical Activity Status from High-Throughput Screening Assays
Source: PLoS One. 2015 Dec 14;10(12):e0144426. doi: 10.1371/journal.pone.0144426 (PMC4682830; doi:10.1371/journal.pone.0144426)
Supplement: S6 Text — (DOCX) [file pone.0144426.s009.docx]

# **Mining chemical activity status in high-throughput screening assays**

*Othman Soufan^1^, Wail Ba-alawi^1^, Moataz Afeef^1^, Magbubah Essack^1^****,*** *Valentin Rodionov^2^,* *Panos Kalnis^3^ and Vladimir B. Bajic^1,*^*

^1^King Abdullah University of Science and Technology (KAUST), Computational Bioscience Research Center (CBRC), Thuwal 23955-6900, Saudi Arabia. ^2^King Abdullah University of Science and Technology (KAUST), KAUST Catalysis Center (KCC), Thuwal 23955-6900, Saudi Arabia. ^3^King Abdullah University of Science and Technology (KAUST), Infocloud Group, Computer, Electrical and Mathematical Sciences and Engineering Division (CEMSE), Thuwal 23955-6900, Saudi Arabia.

**Supporting Information Text 6**

**Another top ranked prediction list using DRAMOTE for potential drugs interacting with 17β-HSD10 protein’s target in humans**

In order to show that DRAMOTE can be used to suggest potential drugs for diseases different from those related to TSHR, AID 886 dataset was chosen to find another list of top 10 ranked interacting drugs. The following table highlights the top ranked predictions by DRAMOTE for 17beta-Hydroxysteroid Dehydrogenase Type 10 (17β-HSD10) protein target. Monitoring of 17β-HSD10 and its complex, has been found to, represent a potential target for Alzheimer disease diagnostics (1).

Table 1: Top 10 ranked predictions by DRAMOTE for BioAssay 886 with 17beta-Hydroxysteroid Dehydrogenase Type 10 protein target

| Rank | DrugBank ID | Drug Name | DrugBank Description | Ensemble System Score |
| --- | --- | --- | --- | --- |
| 1 | DB00962 | Zaleplon | Zaleplon is a sedative/hypnotic, mainly used for insomnia. | 0.950 |
| 2 | DB00438 | Ceftazidime | Semisynthetic, broad-spectrum antibacterial used especially for Pseudomonas and other gram-negative infections in debilitated patients. | 0.942 |
| 3 | DB01326 | Cefamandole | Cefamandole (INN, also known as cephamandole) is a broad-spectrum cephalosporin antibiotic. | 0.941 |
| 4 | DB04570 | Latamoxef | It has been proposed especially for the meningitides because it passes the blood-brain barrier and for anaerobic infections. | 0.940 |
| 5 | DB06605 | Apixaban | Apixaban is an oral, direct, and highly selective factor Xa (FXa) inhibitor (of both free and prothrombinase-bound FXa independently of antithrombin III) for the prevention and treatment of thromboembolic diseases. | 0.937 |
| 6 | DB01030 | Topotecan | An antineoplastic agent used to treat ovarian cancer. It works by inhibiting DNA topoisomerases, type I. | 0.936 |
| 7 | DB01416 | Cefpodoxime | It is commonly used to treat acute otitis media, pharyngitis, and sinusitis. | 0.921 |
| 8 | DB00923 | Ceforanide | Ceforanide is a second-generation parenteral cephalosporin antibiotic. | 0.920 |
| 9 | DB01415 | Ceftibuten | Cefalexin is used to treat acute bacterial exacerbations of chronic bronchitis (ABECB), acute bacterial otitis media, pharyngitis, and tonsilitis. | 0.919 |
| 10 | DB01076 | Atorvastatin | It is used for lowering cholesterol. | 0.916 |

# **References**

1. Marchais-Oberwinkler S, Henn C, Möller G, Klein T, Negri M, Oster A, et al. 17β-Hydroxysteroid dehydrogenases (17β-HSDs) as therapeutic targets: protein structures, functions, and recent progress in inhibitor development. The Journal of steroid biochemistry and molecular biology. 2011;125(1):66-82.
